# Time Series Transcriptomic Analysis by RNA Sequencing Reveals a Key Role of PI3K in Sepsis-Induced Myocardial Injury in Mice

**DOI:** 10.3389/fphys.2022.903164

**Published:** 2022-06-01

**Authors:** Xiao Yan, Yun-Long Zhang, Xiao Han, Pang-Bo Li, Shu-Bin Guo, Hui-Hua Li

**Affiliations:** ^1^ Emergency Medicine Clinical Research Center, Beijing Chao-Yang Hospital, Capital Medical University, Beijing Key Laboratory of Cardiopulmonary Cerebral Resuscitation, Beijing, China; ^2^ School of Public Health, Hangzhou Normal University, Hangzhou, China

**Keywords:** cecal ligation and puncture, septic cardiomyopathy, RNA sequencing, time series gene expression profiling, PIK3R5

## Abstract

Septic cardiomyopathy is the main complication and cause of death of severe sepsis with limited therapeutic strategy. However, the molecular mechanism of sepsis-induced cardiac injury remains unclear. The present study was designed to investigate differentially expressed genes (DEGs) involved in the pathogenesis of septic cardiomyopathy induced by cecal ligation and puncture (CLP) in mice. Male C57BL/6J mice (8–10 weeks old) were subjected to CLP with 21-gauge needles for 24, 48, and 72 h. Myocardial function was assessed by echocardiography. The pathological changes of the heart were evaluated by hematoxylin and eosin as well as immunohistochemical staining. Time series RNA sequencing was utilized to investigate the gene expression profiles. CLP surgery resulted in a significant decrease of animal survival rate and left ventricle contractile function, and an increase in cardiac dilation and infiltration of proinflammatory cells including Mac-2^+^ macrophages in a time-dependent manner. RNA sequencing identified 5,607 DEGs in septic myocardium at 24, 48, and 72 h after CLP operation. Moreover, gene ontology analysis revealed that these DEGs were mainly associated with the biological processes, including cell adhesion, immune system process, inflammatory response, and positive regulation of cell migration. KEGG pathway enrichment analysis indicated that *Staphylococcus aureus* infection, osteoclast differentiation, leishmaniasis, and ECM-receptor interaction were significantly altered in septic hearts. Notably, Pik3r1 and Pik3r5 were localized in the center of the gene co-expression network, and were markedly upregulated in CLP-induced septic myocardium. Further, blocking PI3Kγ by the specific inhibitor CZC24832 significantly protected against sepsis-induced cardiac impairment. The present study uncovers the gene expression signatures of CLP-induced myocardial injury and sheds light on the role of Pik3r5 in septic cardiomyopathy.

## Introduction

Sepsis is defined as a life-threatening organ dysfunction secondary to severe infection and represents the major cause of death, accounting for approximately 19.7% of the global death (Singer et al., 2016; Rudd et al., 2020). The imbalance in the host immune system response, including cytokine storm and immune paralysis caused by apoptosis, suppression, and exhaustion of immune cells, subsequently induces cell dysfunction and organ failure, especially in the cardiovascular system (Singer et al., 2016; van der Poll et al., 2017). Sepsis-induced acute myocardial injury is a complex pathophysiological process characterized by the relative imbalanced distribution of blood volume caused by peripheral vasodilation and insufficient cardiac output (Beesley et al., 2018). Animal and human studies have demonstrated that cardiac dysfunction may serve as a major complication and cause of death of sepsis and septic shock (Merx and Weber, 2007).

Accumulating evidence have revealed that multiple cellular and molecular regulators participate in the development of septic cardiomyopathy, including pro-inflammatory factors, cardiomyocyte calcium handling, oxidative stress, mitochondrial dysfunction, and apoptosis, which ultimately result in cardiac dysfunction and adverse remodeling (Kawai and Akira, 2010; Liu et al., 2017). Activation of NLRP3 inflammasome plays an important role in the inflammation, apoptosis, and pyroptosis in septic myocardium (Li N. et al., 2019). In addition, autophagy exhibits dynamic changes in the development of sepsis-induced cardiac dysfunction (Sun et al., 2018). Several signaling pathways are involved in the pathogenesis of sepsis-induced myocardial injury, such as PI3K/AKT, MAPKs, TLR4/MyD88/NF-κB, and gp130/JAK/STAT (Kawai and Akira, 2010; Clere-Jehl et al., 2020). Moreover, the transcriptomic analysis of peripheral blood leucocytes from patients with sepsis identifies several key regulatory genetic variants, which are involved in the hypoxic response, the switch to glycolysis, mediators of endotoxin tolerance, T-cell activation, and viral defense, which may participate in the pathogenesis of sepsis (Davenport et al., 2016). However, the precise mechanism underlying sepsis-induced myocardial injury remains unknown.

In the present study, we performed time-series RNA sequencing to evaluate the gene expression profiles of the heart during cecal ligation and puncture (CLP)-induced sepsis, and attempted to understand the transcriptomic patterns in the initiation and development of septic cardiomyopathy. Our results revealed that 5,607 DEGs were identified in septic myocardium at 24, 48, and 72 h after CLP operation, and were mainly associated with cell adhesion, immune system process, and inflammatory response. We also demonstrated that PI3K subunits particularly Pik3r5 (Phosphoinositide-3-Kinase Regulatory Subunit 5, also known as p101) were localized in the center of the gene co-expression network, and played a critical role in CLP-induced septic myocardium.

## Materials and Methods

### Animal Model and Treatment

Wild-type (WT) male C57BL/6J mice (8–10 weeks old, n = 6 or 10 per group) were subjected to cecal ligation and puncture (CLP) and the observations were made 24, 48, and 72 h post-CLP (Rittirsch et al., 2009). In brief, mice were anesthetized with ketamine (0.2 g/kg, i.p.) and xylazine (0.01 g/kg, i.p.). After adequate exposure of the abdominal cavity, the cecum was exteriorized and ligated with nonabsorbable 4–0 silk distal to the ileocecal valve. A 21-gauge needle was used to puncture the distal end of the cecum. The peritoneum was closed, and mice were resuscitated by subcutaneous injection of 1 ml prewarmed saline. Sham-operated mice underwent the same surgical procedure but without CLP. The PI3Kα/δ/β inhibitor LY294002 (Selleck, Houston, TX, United States) was intraperitoneally administered (10 mg/kg/day per mouse) in mice beginning 1 day before CLP operation. Animals were orally gavaged with vehicle or the PI3Kγ specific inhibitor CZC24832 (Selleck, 10 mg/kg/day) 1 day before CLP surgery. The *in vivo* dosages were selected according to the previous studies (Bergamini et al., 2012; Fan et al., 2017). All mice were housed under a 12 h light-dark cycle in a pathogen-free barrier facility with free access to regular chow diet and water ad libitum.

All experimental procedures were approved by the Animal Care and Use Committee of Capital Medical University (AEEI-2020-155) and conformed to the Guide for the Care and Use of Laboratory Animals published by the United States National Institutes of Health.

### Echocardiography

Mice were anesthetized with 1.5% isoflurane and underwent 2-dimensional M-mode echocardiography at each time point after CLP surgery with a 30 MHz probe (Vevo 1100 system; VisualSonics, Toronto, ON, Canada). The measurements of the left ventricular anterior wall thickness (LVAW) at diastole and systole, posterior wall thickness (LVPW) at diastole and systole, inner diameter (LVID) at diastole and systole, fractional shortening (FS), and ejection fraction (EF) were performed using M-mode tracings. At least three consecutive cardiac cycles were recorded and averaged for each measurement (Wang et al., 2018; Yan et al., 2019).

### Histopathological Examination

Mice were sacrifices with an overdose of anesthesia (pentobarbitone sodium 100 mg/kg, i.v.). Heart samples were dissected, fixed in 4% paraformaldehyde and embedded in paraffin. The heart sections (5 μm in thickness) were stained with haematoxylin and eosin (H&E) according to the standard procedures (Wang et al., 2018; Yan et al., 2019). The immunohistochemical staining of heart sections was performed with anti-Mac-2 antibody (1:200; Abcam, MA, United States). The images were captured by Nikon Labophot 2 microscope (Nikon, Tokyo, Japan) and analyzed using ImageJ software (United States National Institutes of Health, Bethesda, MD).

### RNA Sequencing and Bioinformatics Analysis

Total RNA was extracted from freshly isolated hearts (n = 5 per group) using TRIzol (Invitrogen, Carlsbad, CA, United States) according to the manufacturer’s instructions. The mRNA was enriched, fragmented, and synthesized to the double-stranded cDNA, followed by quality control of the sample library using Agilent 2100 bioanalyzer and quantitative real-time PCR (qPCR). The sequencing was then performed using Illumina NovaSeq as previously described (Li Y. et al., 2019). The data of gene expression files are available at the Gene Expression Omnibus website under Accession No. GSE171546. For bioinformatics analysis, the differentially expressed genes (DEGs), gene ontology (GO), KEGG pathways, series test of clusters, and gene co-expression networks were utilized to enrich the dataset for genes associated with sepsis-induced myocardial injury as described previously (Zhang et al., 2018; Wu et al., 2019).

### Verification of RNA Sequencing Results by Quantitative Real-Time PCR Analysis

Total RNAs were isolated from fresh heart tissues with TRIzol reagent and reverse transcribed using a RT Enzyme Mix (Accurate Biotechnology Co., Ltd. Hunan, China) according to the manufacturer’s protocols. The mRNA expression levels of the selected genes including S100a9, Cxcr2, Cxcl1, Uchl1, Ace2, Itgb6, Lrg1, Hmgb2, Itgam, Icam1, Pik3r1, and Pik3r5 were gauged by an iCycler IQ system (Bio-Rad, Hercules, CA) as described previously (Yan et al., 2019; Yan et al., 2020). The transcript quantities were normalized to the value of an endogenous control (GAPDH). The primers were purchased from Sangon Biotech (Shanghai, China) and were listed in Supplementary Table S1.

### Western Blot Analysis

Proteins were isolated from snap-frozen heart samples using RIPA buffer containing protease inhibitors (Solarbio Science Technology Co., China). The protein lysates (40–50 μg) were separated by electrophoresis in 10% SDS-PAGE gels, transferred to polyvinylidene difluoride (PVDF) membranes, and then incubated with the primary antibodies against p-AKT (Ser473), AKT, p-p65 (Ser536), p65 (Cell Signaling Technologies, Boston, MA), and GAPDH (Proteintech Group Inc, Rosemont, IL). The horseradish peroxidase-conjugated secondary antibodies were purchased from Cell Signaling Technologies. The signal intensities of blots were evaluated by the ECL Plus chemiluminescent system (Bio-Rad, CA, United States) and were quantified by the NIH ImageJ software and normalized to GAPDH.

### Statistical Analysis

All data are presented as mean ± standard error of the mean (SEM). The normality test (Shapiro–Wilk) was performed to investigate whether the data were normally distributed. The Student *t*-test and one-way ANOVA were utilized to compare the significant difference in normal distribution. Mann–Whitney test was used when the data were not normally distributed. For the analysis of mortality rates after CLP operation, the Kaplan-Meier method was utilized. A *p* value of <0.05 was considered statistically significant.

## Results

### Cecal Ligation and Puncture-Induced Sepsis Increases Lethality and Cardiac Dysfunction in Mice

**FIGURE 1:**
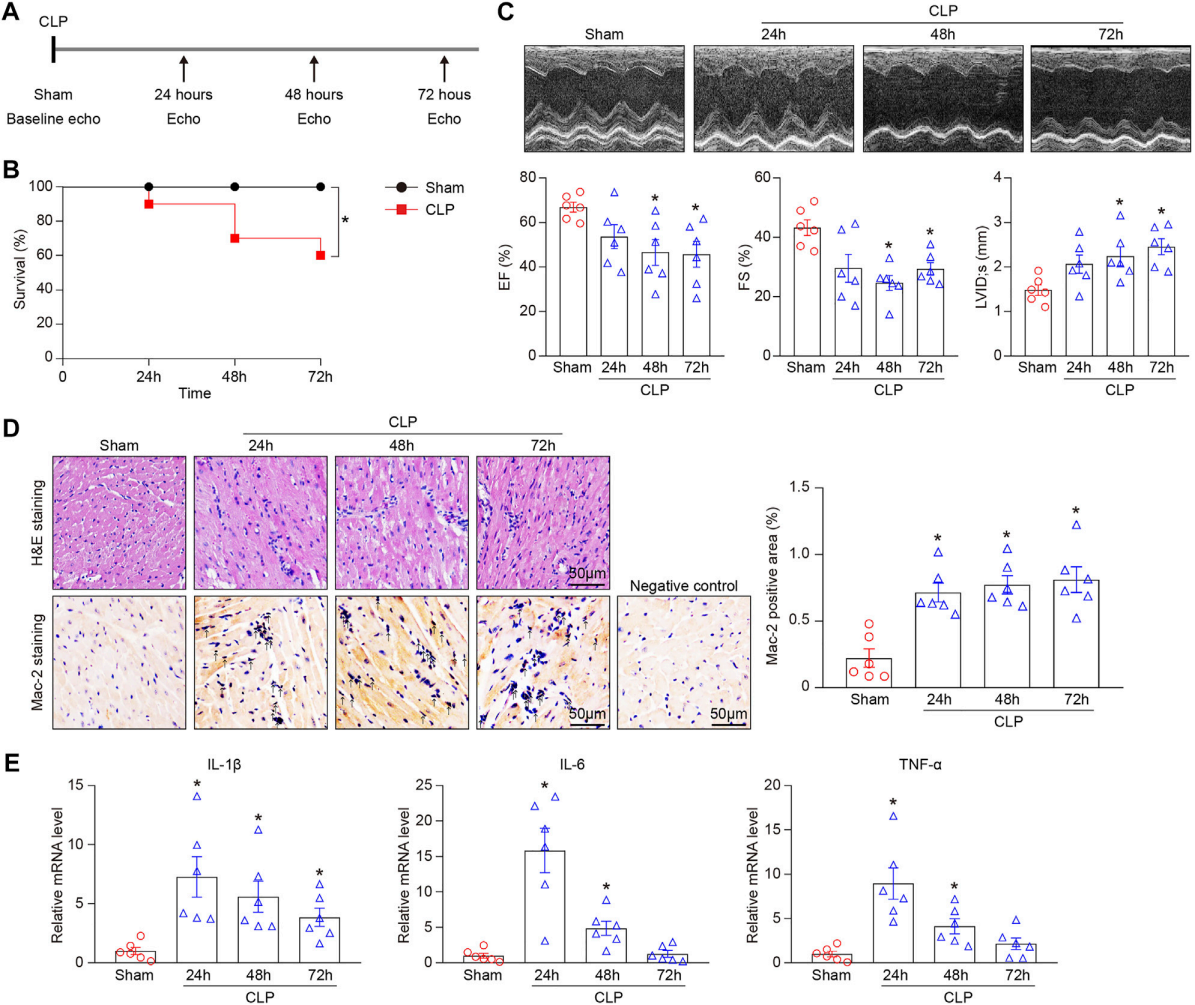
CLP-induced sepsis results in animal death and myocardial injury in mice. Mice were subjected to CLP for 24, 48, and 72 h. **(A)** Schematic diagram of the experimental design. **(B)** Kaplan-Meier survival analysis (n = 10 per group). **(C)** Representative M-mode echocardiographic images of left ventricular at 24, 48, and 72 h after CLP surgery (top). Quantification of EF%, FS%, and LVID;s (bottom, n = 6 per group). **(D)** Representative images of H&E staining (top) and Mac-2 immunohistochemical staining (bottom) of heart sections. Arrows indicate the Mac-2–positive cells. Scale bar: 50 μm. Quantification of Mac-2–positive cells (right, n = 6 per group). **(E)** qPCR analyses of the mRNA expression levels of IL-1β, IL-6, and TNF-α in septic myocardium (n = 6 per group). GAPDH as an internal control. The Kaplan-Meier method was used to analyze the mortality rates after CLP operation. The significance of difference between the means of groups was evaluated using One-way ANOVA following Newman-Keuls multiple comparison test. *p < 0.05 versus sham control.

To elucidate the role of sepsis-induced injury in the heart, WT mice were subjected to sham or CLP operation (21-gauge needle) and the observations were made at 24-, 48-, and 72-hours post-CLP (Figure 1A). Consistent with recent studies (Rittirsch et al., 2009; Zeng et al., 2017), we found that CLP-treated mice had a marked survival defect compared with sham controls (Figure 1B). Echocardiography revealed that CLP time-dependently decreased myocardial contractile function as indicated by left ventricular (LV) EF% and FS% (Figure 1C), and induced LV dilation as indicated by LV internal diameter at systole (LVID;s) (Figure 1C). We next examined the effect of CLP-induced sepsis on myocardial inflammation. H&E and immunohistochemical staining as well as qPCR analysis demonstrated that CLP treatment markedly promoted the accumulation of interstitial proinflammatory cells (as reflected by Mac-2–positive macrophages) and the mRNA expression of IL-1β, IL-6, and TNF-α. (Figures 1D,E).

### Analysis of Gene Expression, Ontology, and Pathways in Mouse Heart After Sepsis

**FIGURE 2:**
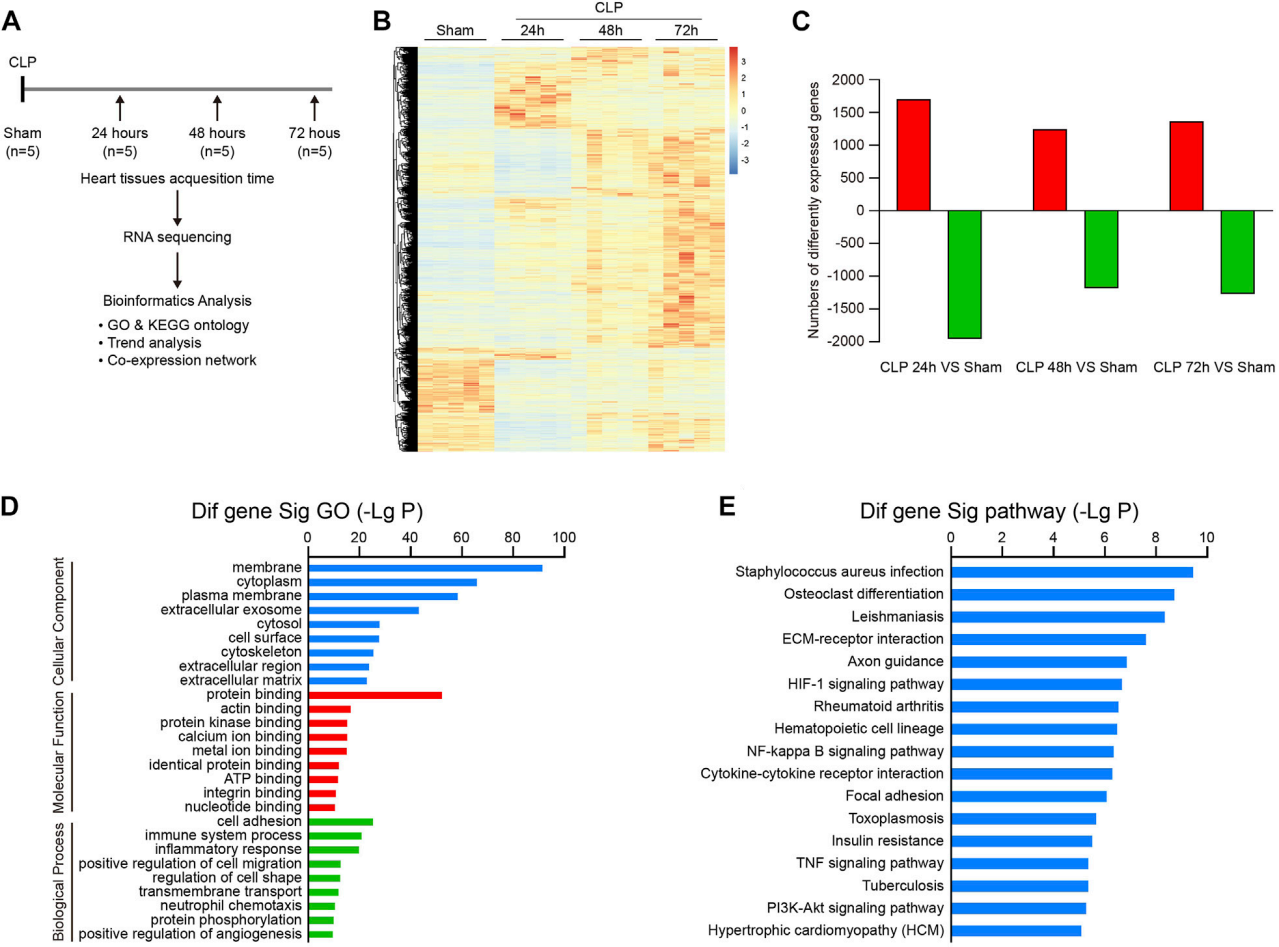
Analysis of gene ontology (GO) terms and KEGG pathways in septic hearts. **(A)** Schematic diagram of the experimental design for RNA sequencing analysis. **(B)** The heat map of differentially expressed genes (DEGs) between septic myocardium and sham control at 24, 48, and 72 h (n = 5 per group). The red or blue color represents upregulation or downregulation of genes, respectively. **(C)** Quantification of significantly upregulated or downregulated genes. **(D)** GO enrichment analysis of DEGs involved in cellular components, molecular functions, and biological processes in hearts after CLP operation. **(E)** KEGG pathways enrichment analysis of DEGs in septic hearts.

To understand the dynamic pathological alterations of sepsis-induced cardiac injury, we performed a time-series transcriptomic analysis (n = 5 per time point) in heart tissues from mice at 24, 48, and 72 h after CLP operation (Figure 2A). RNA sequencing analysis demonstrated that 5,607 differentially expressed genes (DEGs) were significantly changed in hearts after CLP treatment at least at one time point compared with sham controls (*p* < 0.05, FC > 2). Among them, 1,701, 1,242, and 1,365 genes were significantly upregulated, whereas 1,960, 1,181, and 1,271 genes were downregulated at 24, 48, and 72 h, respectively (Figures 2B,C).

We next performed GO enrichment and pathway enrichment analysis to identify biologically significant genes in mouse heart after CLP. GO analysis grouped 5,607 DEGs into 3 distinct categories: cellular component, molecular function, and biological process (Figure 2D). Among them, membrane, cytoplasm, and plasma membrane were the top 3 cellular component terms, whereas protein binding was the most significant term in the molecular function group (Figure 2D). Cell adhesion, immune system process, inflammatory response, positive regulation of cell migration, and regulation of cell shape represented the top five biological process terms (Figure 2D). Furthermore, KEGG pathway analysis showed that 103 pathways were markedly altered in hearts after CLP, including *staphylococcus aureus* infection, osteoclast differentiation, leishmaniasis, ECM-receptor interaction, and axon guidance (Figure 2E). Our data suggest that these GO terms and pathways may participate in the pathogenesis of sepsis-induced myocardial injury and dysfunction.

**FIGURE 3:**
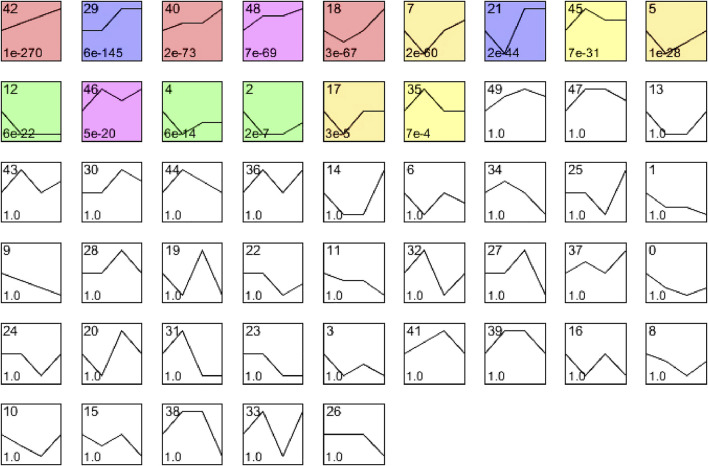
The cluster analysis of DEGs in septic myocardium. Mice were subjected to CLP for 24, 48, and 72 h (n = 5 per group). Based on temporal expression profile similarity, 5,607 DEGs were grouped into 50 gene expression profiles, of which 15 profiles (No. 42, 29, 40, 48, 18, 7, 21, 45, 5, 12, 46, 4, 2, 17, and 35) were significantly altered between septic heart and sham control (colored boxes).

**FIGURE 4:**
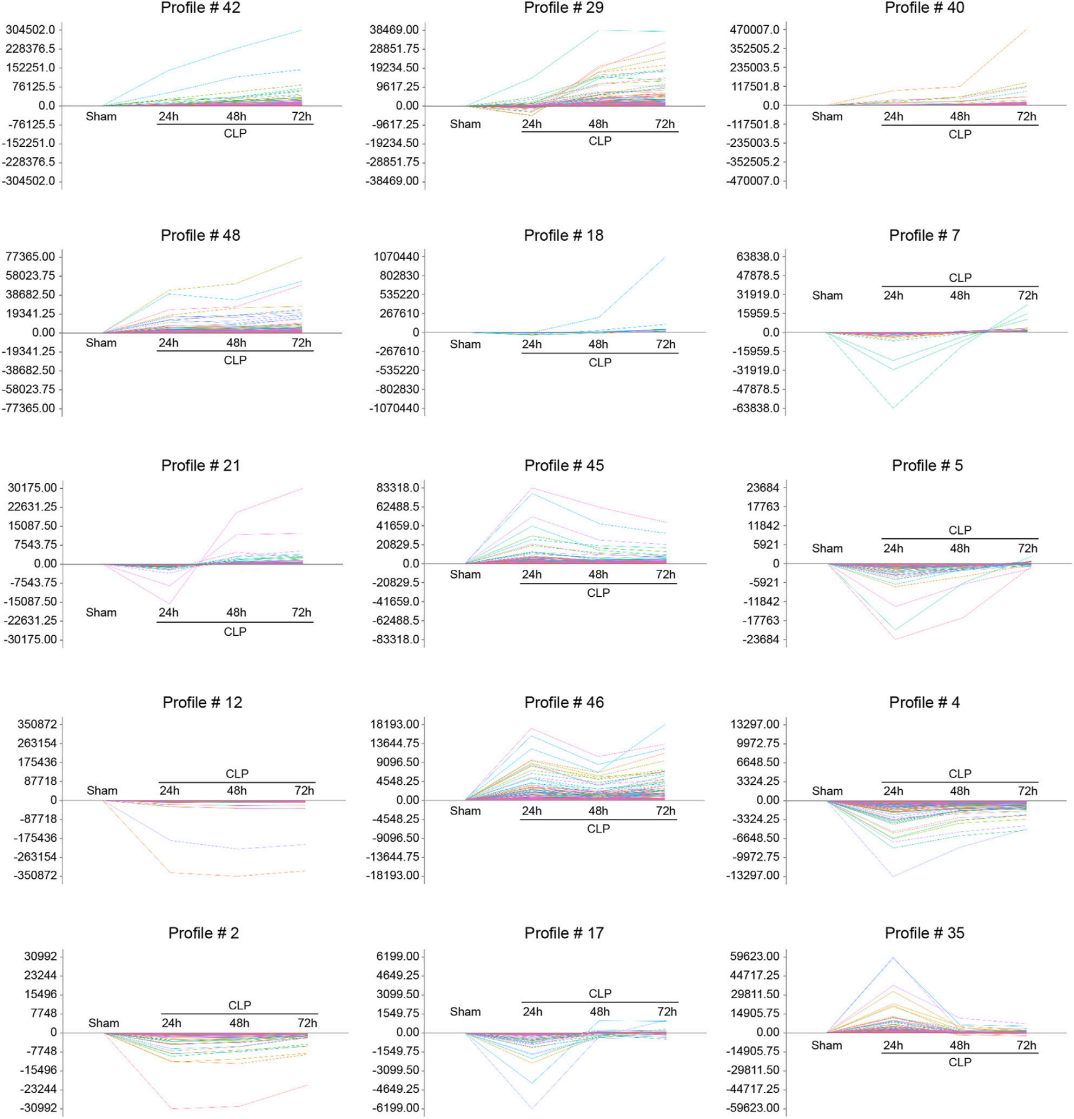
The time-series analysis of co-expressed DEGs during sepsis-induced cardiac injury. Mice were subjected to CLP for 24, 48, and 72 h (n = 5 per group). Gene expression trend analysis was utilized to identify the gene expression patterns of DEGs in 15 profiles (No. 42, 29, 40, 48, 18, 7, 21, 45, 5, 12, 46, 4, 2, 17, and 35) in the heart after CLP surgery at different time points. The vertical axis represents the gene expression levels, and the horizontal axis demonstrates the time points.

To evaluate the temporal expression patterns in hearts at 24, 48, and 72 h after CLP surgery, we use hierarchical cluster analysis based on STEM software and statistical methods to obtain trend models related to time patterns and gene groups closely related to trend models. A total of 5,605 DEGs were classified into 50 profiles, of which 15 profiles (No. 42, 29, 40, 48, 18, 7, 21, 45, 5, 12, 46, 4, 2, 17, and 35) containing 4,527 genes were significantly modified (Figure 3). Furthermore, the expression patterns of DEGs in profile No. 42, 29, 40, and 48 continually increased in a time-dependent manner after CLP operation. The expression of genes in profile NO. 46 increased with 2 peaks at 24 and 72 h. The expression of DEGs in profile NO. 45 and 35 increased with a peak at 24 h, and then decreased at 48 and 72 h. The expression levels of genes in profile No. 18, 17, 21, 5, 4, 2, and 17 were declined at 24 h, and gradually increased at 48 and 72 h. The expression of DEGs in profile No. 12 was continually declined at all time points after CLP (Figure 4).

### Verification of Gene Expression in the Heart During Sepsis

**FIGURE 5:**
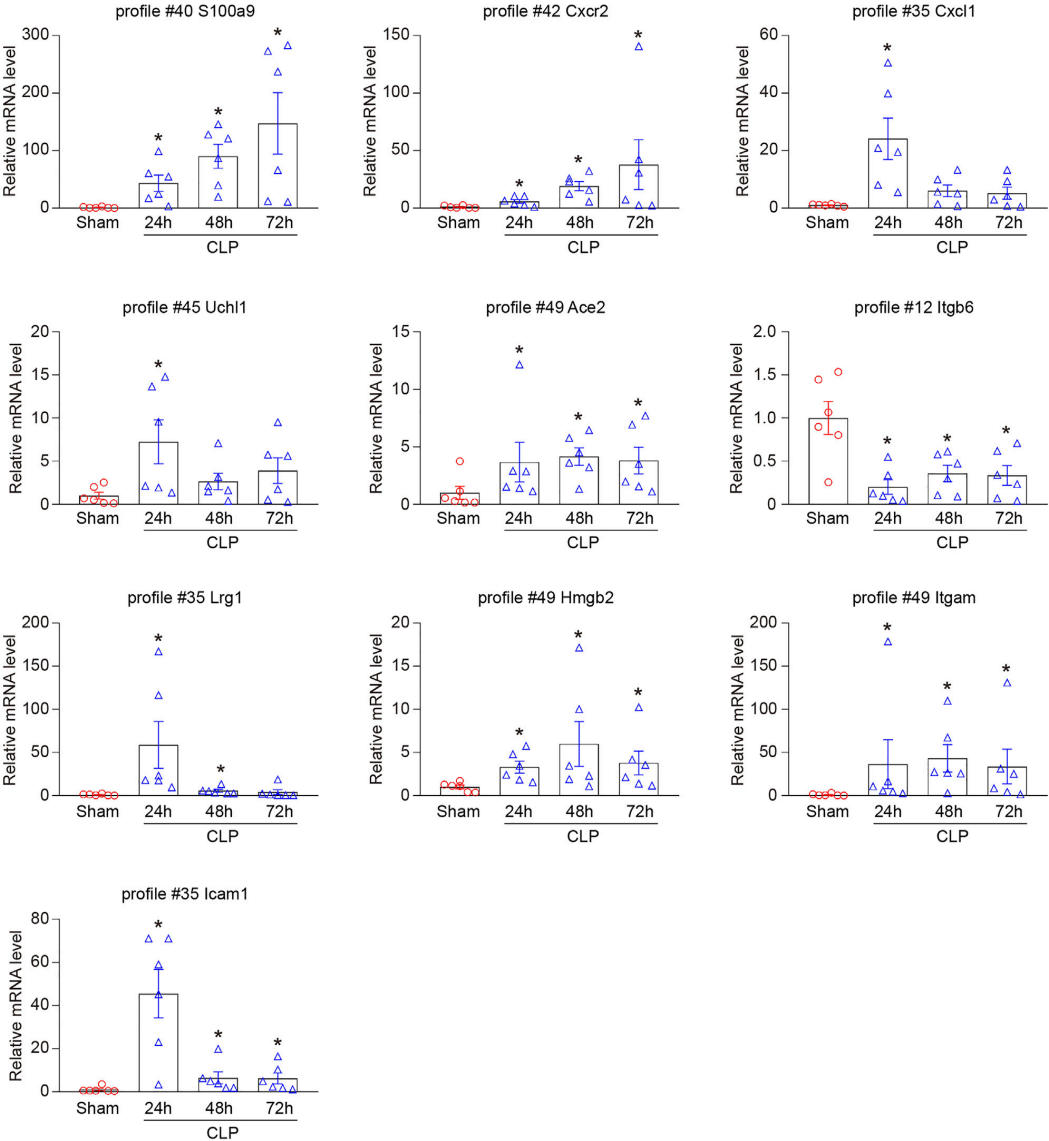
Verification of the selected DEGs in RNA sequencing results. qPCR analyses of the mRNA expression level of 10 DEGs in hearts after CLP operation at different time points (n = 6 per group), including S100a9 (profile #40), Cxcr2 (profile #42), Cxcl1 (profile #35), Uchl1 (profile #45), Ace2 (profile #49), Itgb6 (profile #12), Lrg1 (profile #35), Hmgb2 (profile #49), Itgam (profile #49), and Icam-1 (profile #35). GAPDH as an internal control. The significance of difference between the means of groups was evaluated using One-way ANOVA following Newman-Keuls multiple comparison test. *p < 0.05 versus sham control.

To validate the results from RNA sequencing analysis, we further performed qPCR to evaluate 10 genes randomly selected from significant and non-significant profiles, including S100a9 (profile #40), Cxcr2 (profile #42), Cxcl1 (profile #35), Uchl1 (profile #45), Ace2 (profile #49), Itgb6 (profile #12), Lrg1 (profile #35), Hmgb2 (profile #49), Itgam (profile #49), and Icam-1 (profile #35). As expected, the changes in mRNA expression of these genes were similar to the data from RNA sequencing analysis (Figure 5).

### Analysis of Gene Co-Expression Networks in Heart During Sepsis

**FIGURE 6:**
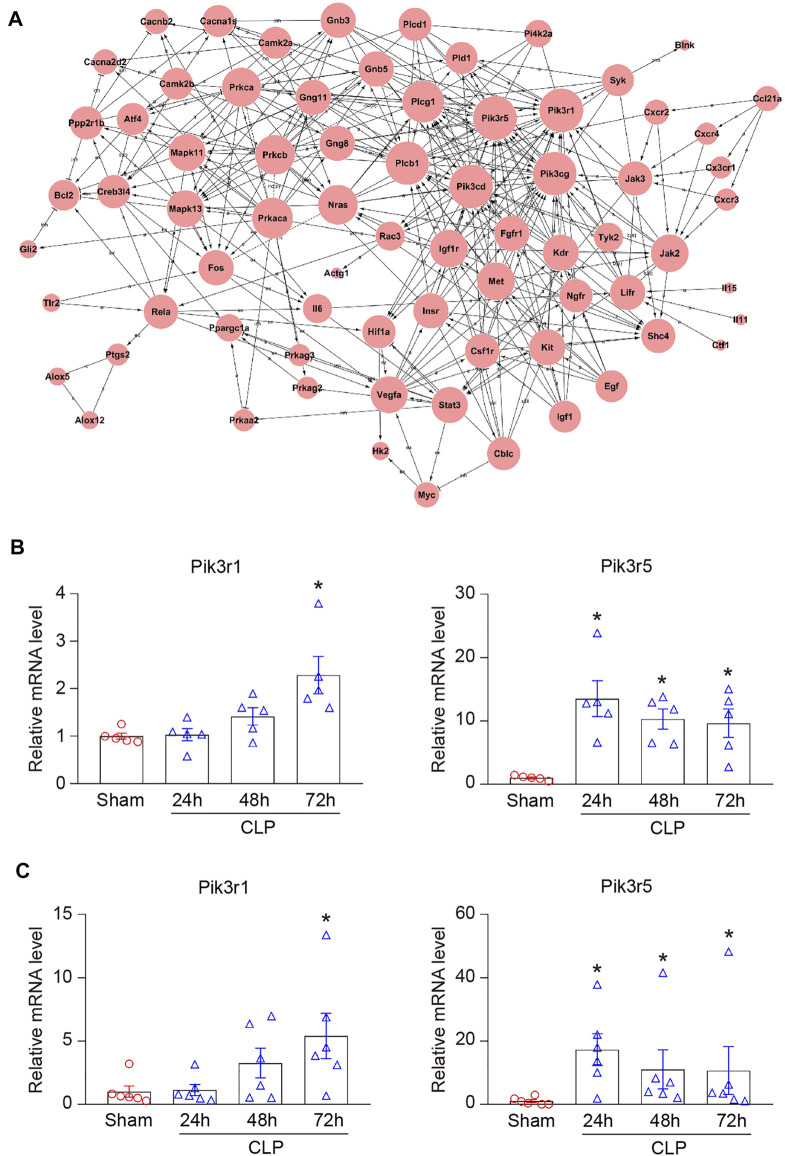
Analysis of gene co-expression network. **(A)** 76 genes selected from 15 significant profiles (No. 42, 29, 40, 48, 18, 7, 21, 45, 5, 12, 46, 4, 2, 17, and 35) were further analyzed using gene co-expression network with k-core algorithm. The cycle node depicts genes, and the edge between two nodes indicates the interaction between genes. **(B)** RNA sequencing analysis of phosphoinositide-3-kinase regulatory subunit 1 (Pik3r1) and Pik3r5 in the heart after CLP surgery at 24, 48, and 72 h (n = 5 per group). **(C)** qPCR analyses of the mRNA expression levels of Pik3r1 and Pik3r5 in septic myocardium (n = 6 per group). GAPDH as an internal control. The significance of difference between the means of groups was evaluated using One-way ANOVA following Newman-Keuls multiple comparison test. *p < 0.05 versus sham control.

To identify the genes that exert central roles in sepsis-induced cardiac injury, 76 candidate genes selected from 15 significant profiles were evaluated using a co-expression analysis. Based on a k-core algorithm including degree, k-core value, and betweenness centrality, we revealed that phosphoinositide-3-kinase regulatory subunit 1 (Pik3r1) and Pik3r5, which directly interact with 26 neighboring genes, possessed the highest degree and located at the center of the gene network (Figure 6A; Supplementary Table S2). Furthermore, our RNA sequencing results demonstrated that the mRNA levels of Pik3r1 and Pik3r5 were continuously upregulated in hearts during CLP operation (Figure 6B). We further performed qPCR analysis to verify the effect of CLP and lipopolysaccharide (LPS) on the expression of Pik3r1 and Pik3r5 *in vivo* and *in vitro*. Accordingly, the mRNA expression levels of Pik3r1 and Pik3r5 were increased in the heart after CLP surgery and in LPS-treated cardiomyocytes (Figure 6C; Supplementary Figure S1).

**FIGURE 7:**
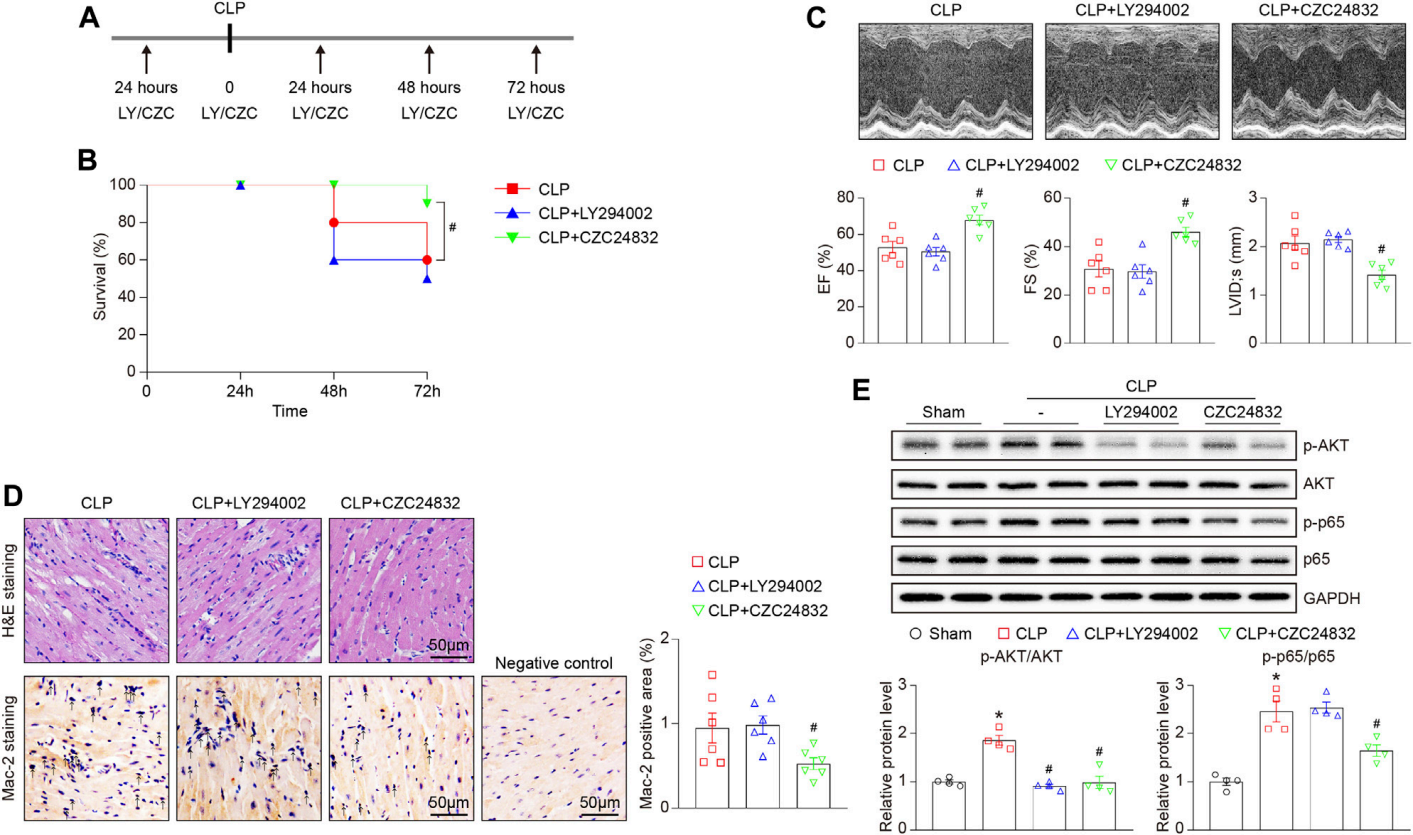
Effects of LY294002 and CZC24832 on CLP-induced septic cardiomyopathy. Mice were pretreated with LY294002 and CZC24832, and then subjected to CLP for 72 h. **(A)** Schematic diagram of the experimental design. **(B)** Kaplan–Meier survival analysis (n = 10 per group). **(C)** Representative M-mode echocardiographic images of left ventricular at 72 h after CLP surgery (top). Quantification of EF%, FS%, and LVID;s (bottom, n = 6 per group). **(D)** Representative images of H&E staining (top) and Mac-2 immunohistochemical staining (bottom) of heart sections. Arrows indicate the Mac-2–positive cells. Scale bar: 50 μm. Quantification of Mac-2–positive cells (right, n = 6 per group). **(E)** Immunoblotting analysis of the protein levels of p-AKT, AKT, p-p65, and p65 in the heart tissues (top). Quantification of the relative protein levels (bottom, n = 4). GAPDH as an internal control. After the normality test (Shapiro–Wilk), the student t-test was used to compare the significant difference between two groups in normal distribution, and the Mann–Whitney test was performed for the data that were not normally distributed. *p < 0.05 versus sham control, ^#^ *p* < 0.05 versus CLP.

To verify the role of Pik3r1 and Pik3r5 in CLP-induced septic cardiomyopathy, mice were treated with the PI3Kα/δ/β inhibitor LY294002 or the PI3Kγ specific inhibitor CZC24832, respectively (Figure 7A). After 72 h CLP surgery, administration of CZC24832 but not LY294002 significantly reduced animal lethality (Figure 7B), which was associated with improvement of cardiac dysfunction (as indicated by EF, FS, and LVID;s) and infiltration of pro-inflammatory cells (demonstrated by H&E and Mac-2 immunohistochemistry staining) (Figures 7C,D). Moreover, LY294002 and CZC24832 markedly downregulated p-AKT protein expression in septic myocardium (Figure 7E). However, treatment of CZC24832 but not LY294002 significantly attenuated CLP-induced NF-κB p65 activation in the heart (Figure 7E). Collectively, these data indicate that Pik3r5 may exert an important role in the regulation of septic cardiomyopathy.

## Discussion

In the present study, we used time series transcriptome analysis to identify the dynamic gene expression profiles, GO terms, and signaling pathways during the development of CLP-induced septic cardiomyopathy in mice. Our findings revealed that in the heart in response to 24, 48, and 72 h of CLP surgery, a total of 5,607 genes were differentially expressed, which were associated with the cell adhesion, immune system process, inflammatory response, *Staphylococcus aureus* infection, and osteoclast differentiation. The gene expression pattern analysis demonstrated that these DEGs were classified into 50 profiles, of which 15 profiles (4,527 genes) were significantly altered. Moreover, Pik3r5 was located in the center of the gene co-expression network, and may play an important role in the progression of sepsis-induced cardiac injury.

LV dilatation and decreased EF are characteristic changes in patients with septic cardiomyopathy (Pathan et al., 2004; Cecconi et al., 2018; Ehrman et al., 2018). Sepsis-induced myocardial injury is considered to be a complex pathophysiological process, including interstitial inflammatory infiltrate, mitochondrial dysfunction, apoptosis, reactive oxygen species (ROS), endothelial impairment, and renin-angiotensin system (RAS) (Liu et al., 2017; Beesley et al., 2018). Inflammatory response exerts a key role in the initiation and development of sepsis-induced cardiomyocyte dysfunction. In the early stage of sepsis, pathogen-associated molecular patterns (PAMPs) such as LPS or IFN-γ activate toll-Like receptors (TLRs) and downstream signaling pathways, induce the production of pro-inflammatory cytokines including tumor necrosis factor (TNF)-α, interleukin (IL)-1, and IL-6, and promotes myocardial inflammatory cell infiltration (Liu et al., 2017). In addition, myocardial injury leads to the release of a variety of damage-associated molecular patterns (DAMPs), including S100 protein, ROS, HMGB1, and heat shock proteins, which subsequently aggravate myocardial inflammation and dysfunction (Liu et al., 2017). Several studies have demonstrated that S100a8/a9 initiates LPS-induced cardiac dysfunction by the receptor for advanced glycation end products (Boyd et al., 2008). We and others have revealed that chemokine CXCL1 and its receptor CXCR2 exert a critical role in the accumulation of neutrophils, monocytes, and lymphocytes into the injured heart (Tarzami et al., 2003; Abu Nabah et al., 2007; Wang et al., 2018; Zhang et al., 2020). Intercellular adhesion molecule-1 (ICAM-1) is essential for the leukocyte-dependent myocardial dysfunction in LPS-treated mice (Davani et al., 2006). Furthermore, LPS stimulation enhanced Uchl1 expression in macrophages with a peak at 24 h, and then decreased at 48 h (Huang et al., 2022). Consistent with the recent findings, we found that CLP-induced sepsis led to reducing survival, left ventricular dilatation, cardiac dysfunction, and inflammatory cell infiltration in a time-dependent manner (Figures 1B–D). Accordingly, CLP operation resulted in significantly increased gene expression levels of S100a9, Cxcr2, Cxcl1, ICAM-1, and Uchl1 in hearts (Figure 5). Among them, the mRNA levels of Cxcl1, ICAM-1, and Uchl1 peaked at 24 h and then gradually declined at 48 and 72 h, suggesting that Cxcl1, ICAM-1, and Uchl1 may play an important role in the early stage of CLP-induced septic cardiomyopathy.

Previous studies have reported that several signaling pathways are involved in the pathogenesis of septic cardiomyopathy, including TLRs/MyD88/NF-κB, gp130/JAK/STAT, and PI3K/AKT (Kawai and Akira, 2010; An et al., 2016; Clere-Jehl et al., 2020). For example, it is well known that TLR4 and TLR2 play an important role in mediating the development in sepsis-induced myocardial injury (Spiller et al., 2008; Alves-Filho et al., 2009; Zou et al., 2010). On the contrary, TLR7 upregulates cAMP/PKA/PLN signaling and improves septic cardiomyopathy in LPS-treated mice (Saiyang et al., 2021). In addition to the classic TLRs/MyD88/NF-κB pathway, STING/IRF3/NLRP3 signaling is involved in the LPS-induced cardiac inflammation, apoptosis, and dysfunction (Li N. et al., 2019). However, the precise molecular mechanism of sepsis-induced myocardial impairment is not fully understood. An immune cell-specific study has found that MMP9, ARG1, CXCL3, SOCS3, CCR7, and IL-10 are differentially expressed in T cells and monocytes from sepsis patients compared with healthy individuals (Washburn et al., 2019). The microarray analysis has revealed that NF-κB, JAK/STAT, and MAPK/PI3K signaling pathways are significantly modified in LPS-treated murine myocardium, which contribute to the dysfunctional immune system and inflammation in sepsis-induced cardiac injury (Ashton et al., 2017). Moreover, transcriptomic studies have demonstrated that the gene expression profiles and biological pathways are dynamically altered in the early phrase of CLP (6–24 h) (Rumienczyk et al., 2021), and Wnt signaling is involved in LPS-induced myocardial injury (Chen et al., 2020). To further explore the gene expression profiles in septic cardiomyopathy, we performed time series RNA sequencing analysis in hearts after CLP operation. Among 5,607 DEGs, Lrg1, Ace2, Itgb6, Hmgb2, Pik3r5, Itgam, Plcb1, Jak2, Vegfa, and Hif1α were associated with the entire course of sepsis-induced myocardial injury, which have been reported previously (Kumagai et al., 2016; Hashida et al., 2017; Narula et al., 2020). For example, Lrg1 (leucine rich alpha-2-glycoprotein 1) plays a key role in TGF-β signaling and cardiac remodeling, and increased serum Lrg1 level is associated with sepsis (Kumagai et al., 2016; Hashida et al., 2017). In keeping with our data, the expression of Lrg1 was highest at 1 day after myocardial infarction and gradually decreased from 4 to 14 days, indicating that Lrg1 may serve as a potential early predictor of cardiovascular diseases such as septic cardiomyopathy (Kumagai et al., 2016). The plasma concentration of RAS regulator Ace2 (angiotensin-converting enzyme 2) is positively correlated with the risk of heart failure, myocardial infarction, and stroke (Narula et al., 2020). Importantly, RNA sequencing and qPCR analysis revealed that PI3K regulatory subunits Pik3r1 and Pik3r5 were markedly upregulated in septic heart at different time points (Figure 6). There are three major classes (I, II, and III) of PI3Ks. Among them, class IA PI3Ks are heterodimeric lipid kinases composed of a regulatory subunit (p85) and a catalytic subunit (p110α, p110β, or p110δ), which are encoded by Pik3ca, Pik3cb, and Pik3cd genes, respectively. Class IB PI3Ks comprise a regulatory p101 subunit and a catalytic p110γ subunit (encoded by Pik3cg) (Vanhaesebroeck et al., 2005). PI3Ks generate lipids that controls several signaling pathways (AKT/eNOS, NADPH oxidase, and TGF-β/Smad), which participate in cardiovascular diseases, including myocardial infarction and diabetes-induced cardiomyopathy (Kim et al., 2008; Pretorius et al., 2009; Lin et al., 2010). As the regulatory subunits, Pik3r1 encodes the p85 protein and binds to the catalytic subunits of Class IA PI3Ks (PI3Kα/δ/β), and Pik3r5 encodes the p101 protein that interacts with the catalytic subunits of Class IB PI3Ks (PI3Kγ) (Vanhaesebroeck et al., 2005). Several studies have shown that PI3K/AKT signaling is involved in LPS-induced inflammation and cardiac dysfunction (Zhang et al., 2007; Zhou et al., 2011; Li et al., 2012). However, the role of different PI3K isoforms in septic cardiomyopathy is unclear. Herein, we revealed that Pik3r1 and Pik3r5 were highly expressed in septic myocardium, and were located in the center of the gene co-expression network (Figure 6). Furthermore, inhibition of PI3Kγ by CZC24832 significantly ameliorated CLP-induced cardiac inflammation and NF-κB activation, whereas the PI3Kα/δ/β inhibitor LY294002 had no significant effect (Figure 7). Thus, our results suggest that Pik3r5 may serve as a key mediator in sepsis-induced myocardial injury.

The limitation of this study is that the precise mechanism of Pik3r1 and Pik3r5 in the pathophysiology of septic cardiomyopathy (such as inflammation, cardiomyocyte calcium handling, and oxidative stress) has not been fully elucidated. Thus, further studies are needed to extrapolate the findings to knockout or transgenic mice and other animal models of sepsis; to explore the molecular mechanism of Pik3r1 and Pik3r5 *in vitro*; and to clarify the cell type-specific mechanisms of septic cardiomyopathy by single-cell RNA sequencing analysis.

Here, using time series RNA sequencing analysis, we found that a total of 5,607 genes were differently expressed in CLP-induced septic myocardium at different time points, and these genes were associated with cell adhesion, immune system process, and inflammatory response. Moreover, we identified that Pik3r1 and Pik3r5 were at the center of the gene co-expression network, and inhibition of PI3Kγ activity had a cardioprotective role in CLP-induced sepsis.

## Data Availability

The datasets presented in this study can be found in online repositories. The names of the repository/repositories and accession number(s) can be found in the article/Supplementary Material.
