# Supplementary material for: Time Series Transcriptomic Analysis by RNA Sequencing Reveals a Key Role of PI3K in Sepsis-Induced Myocardial Injury in Mice
Source: Front Physiol. 2022 Jun 1;13:903164. doi: 10.3389/fphys.2022.903164 (PMC9198581; doi:10.3389/fphys.2022.903164)
Supplement: Supplementary file 1 [file Table1.DOCX]

**Supplementary Table 1**. Primer sequences for RT-PCR analysis

S100A9, S100 calcium binding protein A9; CXCR2, C-X-C motif chemokine receptor 2; CXCL1, C-X-C motif chemokine ligand 1; UCHL1, ubiquitin C-terminal hydrolase L1; ACE2, angiotensin I converting enzyme 2; ITGB6, integrin subunit beta 6; LRG1, leucine rich alpha-2-glycoprotein 1; HMGB2, high mobility group box 2; ITGAM, integrin subunit alpha M; ICAM1, intercellular adhesion molecule 1; PIK3R1, phosphoinositide-3-kinase regulatory subunit 1; PIK3R5, phosphoinositide-3-kinase regulatory subunit 5; GAPDH, glyceraldehyde 3-phosphate dehydrogenase.

| Gene | Forward primer (5’-3’) | Reverse primer (5’-3’) |
| --- | --- | --- |
| S100A9 | ATACTCTAGGAAGGAAGGACACC | TCCATGATGTCATTTATGAGGGC |
| CXCR2 | ATGCCCTCTATTCTGCCAGAT | GTGCTCCGGTTGTATAAGATGAC |
| CXCL1 | CTGGGATTCACCTCAAGAACATC | CAGGGTCAAGGCAAGCCTC |
| UCHL1 | GATGCTGAACAAAGTGTTGGC | GGAGTTTCCGATGGTCTGCTT |
| ACE2 | TCCAGACTCCGATCATCAAGC | GCTCATGGTGTTCAGAATTGTGT |
| ITGB6 | ATGGGGATTGAGCTGGTCTG | GACAGGTGGGTGAAATTCTCC |
| LRG1 | TTGGCAGCATCAAGGAAGC | CAGATGGACAGTGTCGGCA |
| HMGB2 | GCTCGTTATGACAGGGAGATG | TTGCCCTTGGCACGGTATG |
| ITGAM | CCATGACCTTCCAAGAGAATGC | ACCGGCTTGTGCTGTAGTC |
| ICAM1 | GTGATGCTCAGGTATCCATCCA | CACAGTTCTCAAAGCACAGCG |
| PIK3R1 | ACACCACGGTTTGGACTATGG | GGCTACAGTAGTGGGCTTGG |
| PIK3R5 | TGCTCTGGAGCGATGCTTG | ACCTCTTGGGTCTTTTGTAGGA |
| GAPDH | GGTTGTCTCCTGCGACTTCA | GGTGGTCCAGGGTTTCTTACTC |
