# Supplementary material for: Time Series Transcriptomic Analysis by RNA Sequencing Reveals a Key Role of PI3K in Sepsis-Induced Myocardial Injury in Mice
Source: Front Physiol. 2022 Jun 1;13:903164. doi: 10.3389/fphys.2022.903164 (PMC9198581; doi:10.3389/fphys.2022.903164)
Supplement: Supplementary file 2 [file DataSheet3.DOCX]

**Supplementary Figure Legend**

**Supplementary Figure 1** Effect of LPS stimulation on gene expression of Pik3r1 and Pik3r5 *in vitro*. qPCR analyses of the mRNA expression levels of Pik3r1 and Pik3r5 in neonatal rat cardiac myocytes (NRCMs) treated with lipopolysaccharide (LPS, 1 μg/mL) or vehicle (PBS) for 6 hours (n = 6). After the normality test (Shapiro-Wilk), the student t test was used to compare the significant difference between two groups in normal distribution, and the Mann-Whitney test was utilized for the data that were not normally distributed. **P* < 0.05 versus Control.
