# Supplementary figures and images for: Time Series Transcriptomic Analysis by RNA Sequencing Reveals a Key Role of PI3K in Sepsis-Induced Myocardial Injury in Mice
Source: Front Physiol. 2022 Jun 1;13:903164. doi: 10.3389/fphys.2022.903164 (PMC9198581; doi:10.3389/fphys.2022.903164)

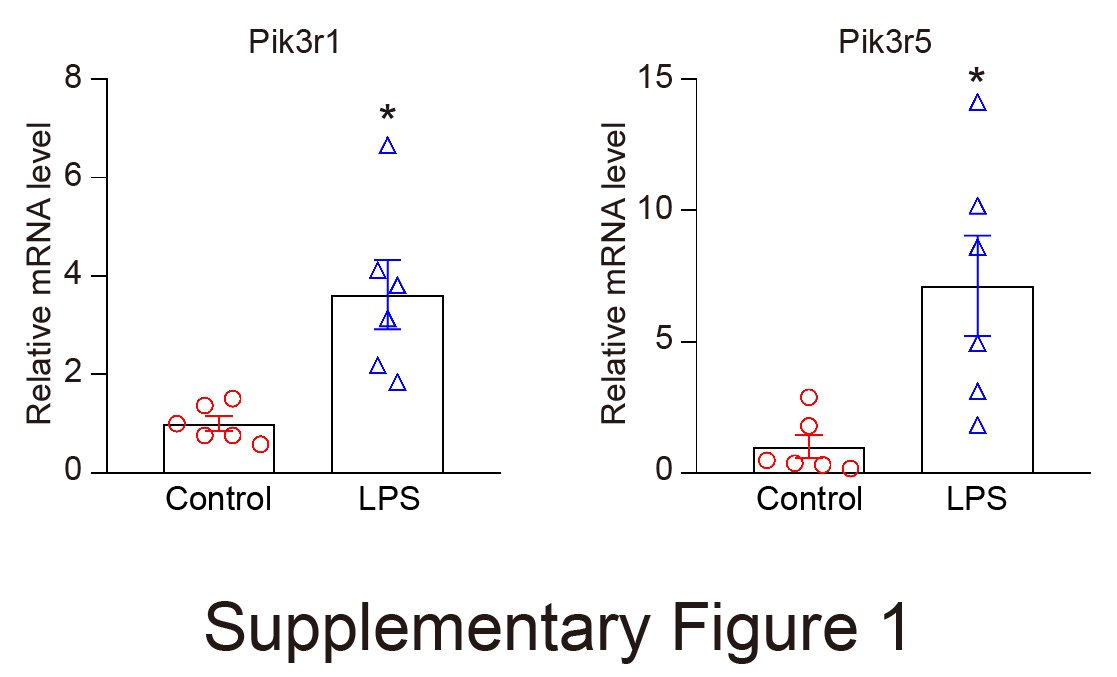

Supplement: Supplementary file 3 [file Image1.JPEG]

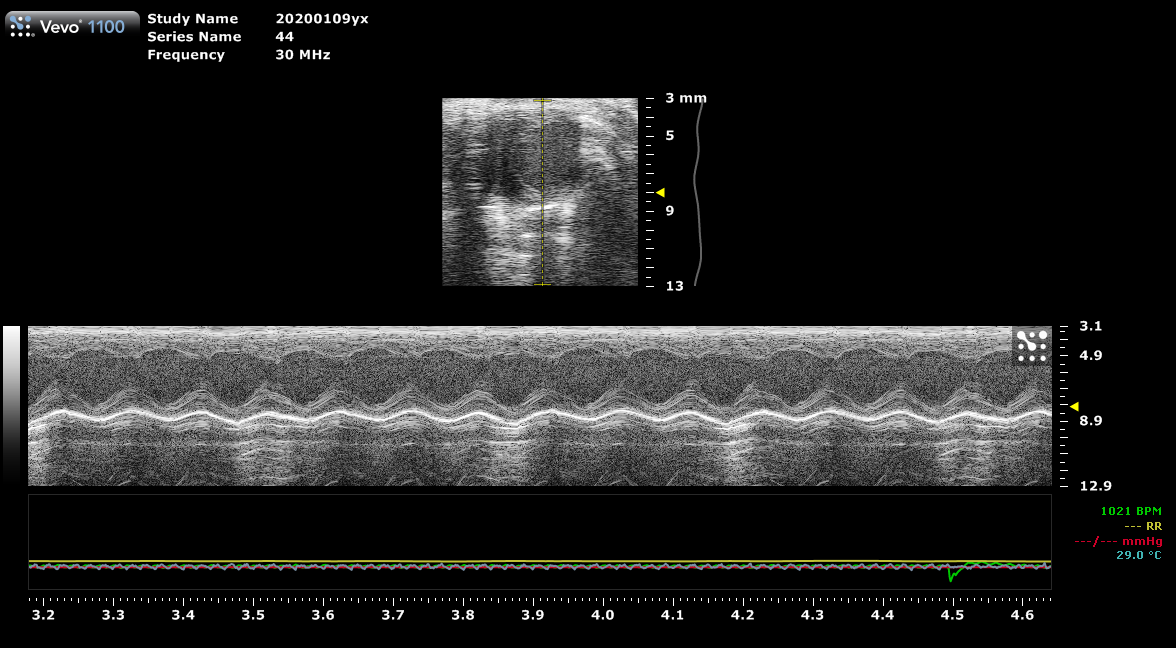

Supplement: Supplementary file 4 [file DataSheet1.zip › Data Sheet 1/Figure 1/Figure 1C/CLP 24h original.tif]

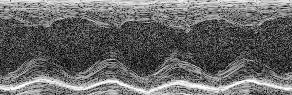

Supplement: Supplementary file 4 [file DataSheet1.zip › Data Sheet 1/Figure 1/Figure 1C/CLP 24h.jpg]

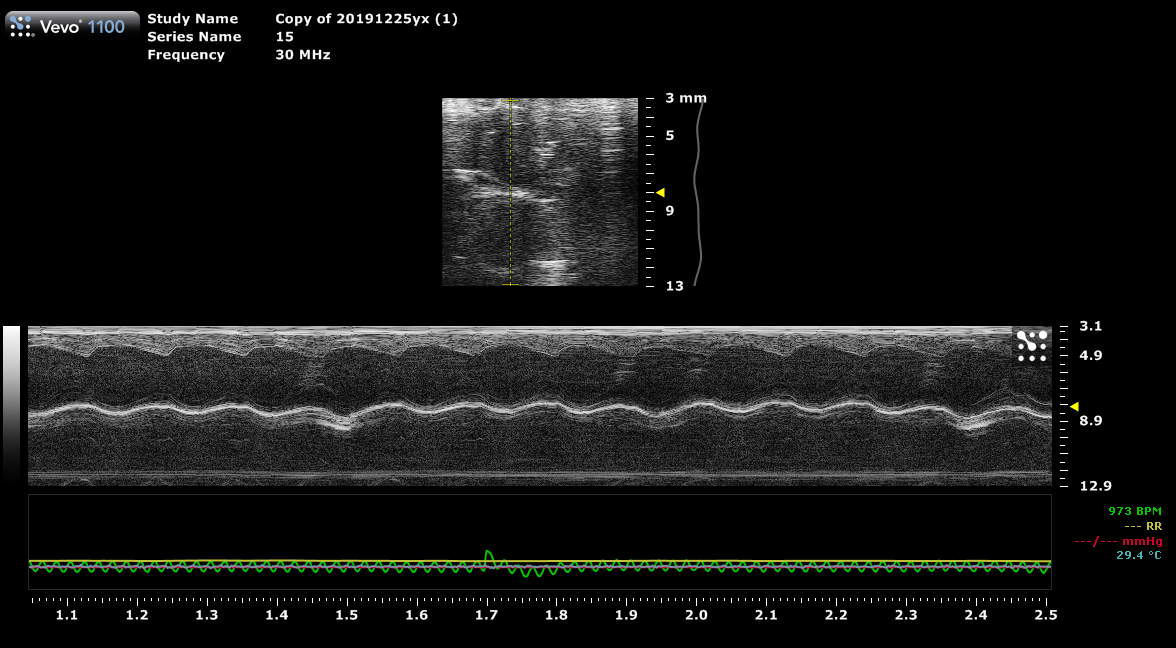

Supplement: Supplementary file 4 [file DataSheet1.zip › Data Sheet 1/Figure 1/Figure 1C/CLP 48h original.tif]

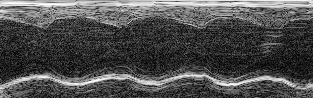

Supplement: Supplementary file 4 [file DataSheet1.zip › Data Sheet 1/Figure 1/Figure 1C/CLP 48h.jpg]

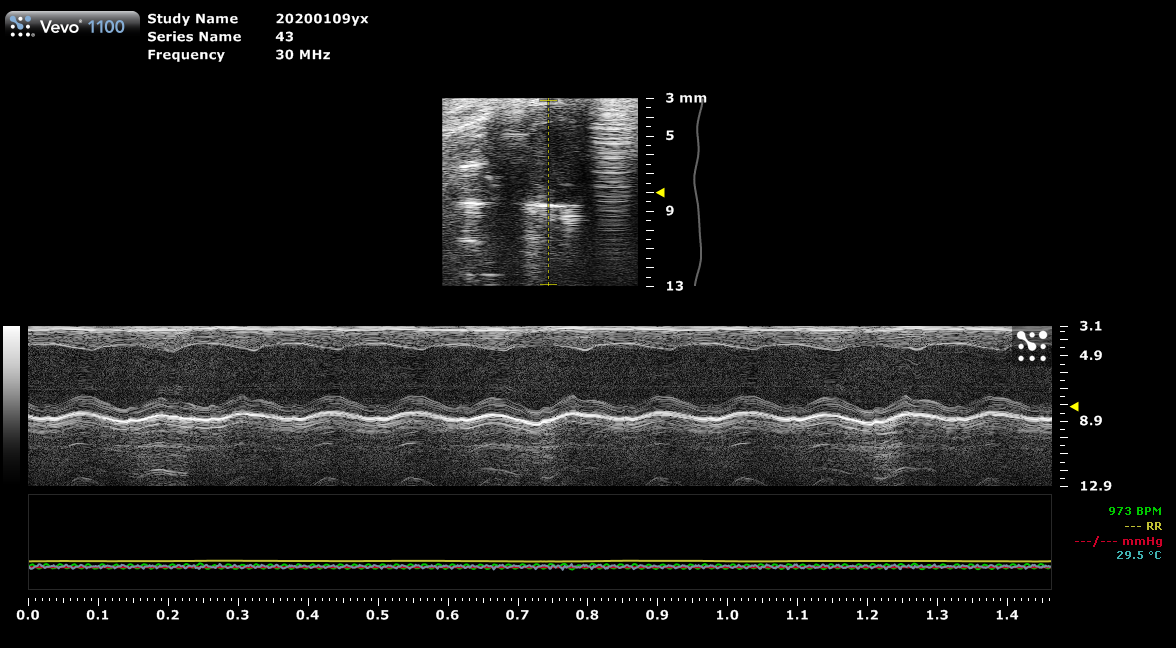

Supplement: Supplementary file 4 [file DataSheet1.zip › Data Sheet 1/Figure 1/Figure 1C/CLP 72h original.tif]

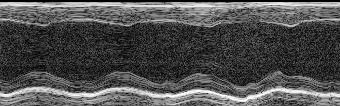

Supplement: Supplementary file 4 [file DataSheet1.zip › Data Sheet 1/Figure 1/Figure 1C/CLP 72h.jpg]

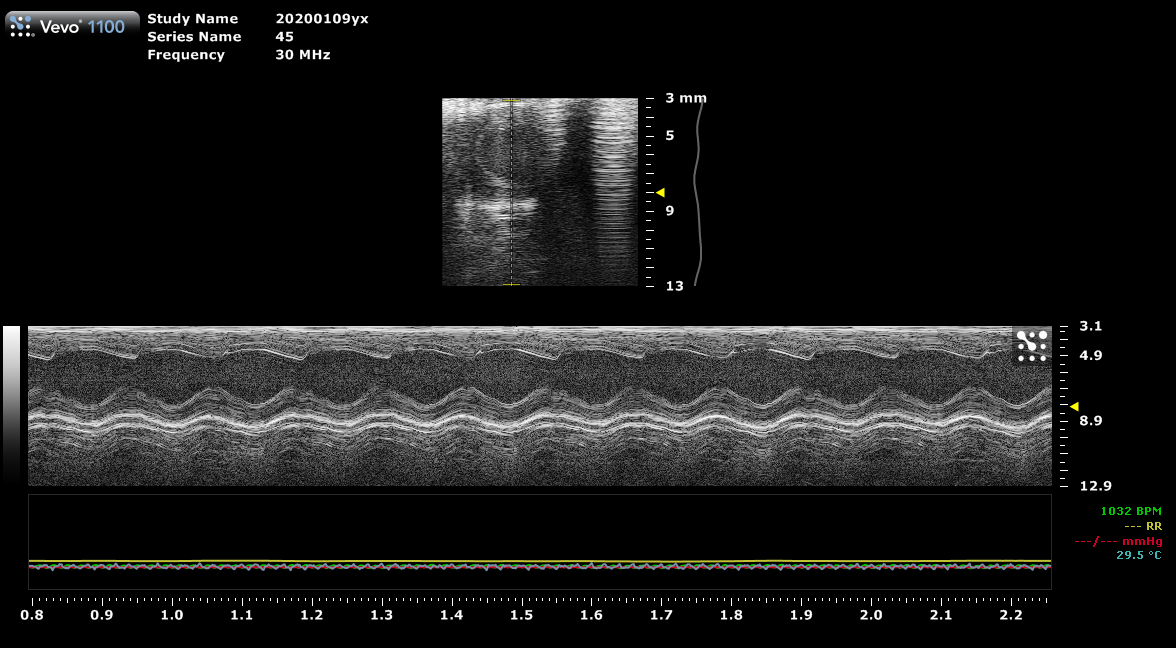

Supplement: Supplementary file 4 [file DataSheet1.zip › Data Sheet 1/Figure 1/Figure 1C/Sham original.tif]

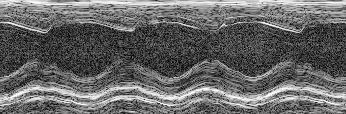

Supplement: Supplementary file 4 [file DataSheet1.zip › Data Sheet 1/Figure 1/Figure 1C/Sham.jpg]

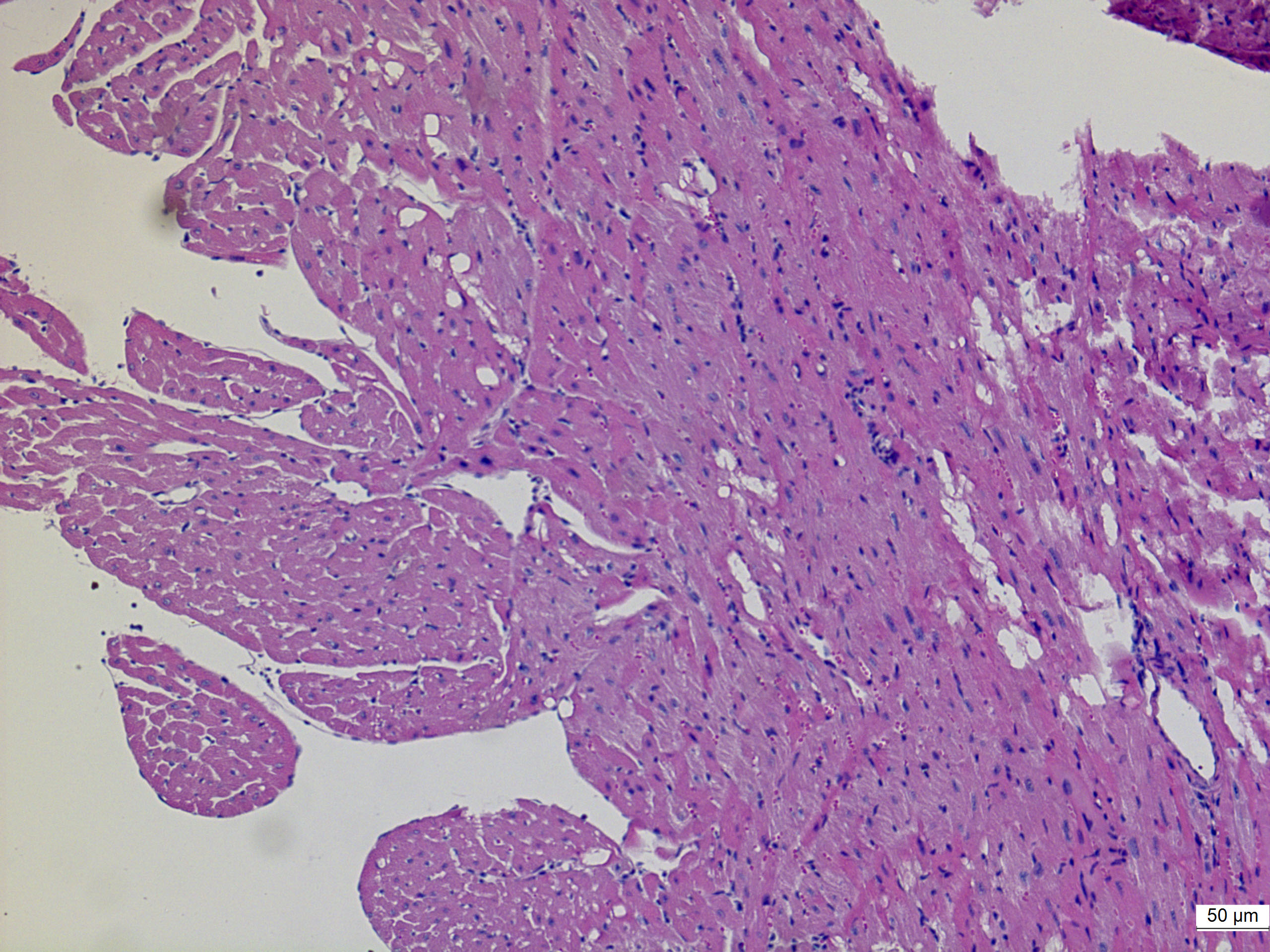

Supplement: Supplementary file 4 [file DataSheet1.zip › Data Sheet 1/Figure 1/Figure 1D/HE/CLP 24h original.jpg]

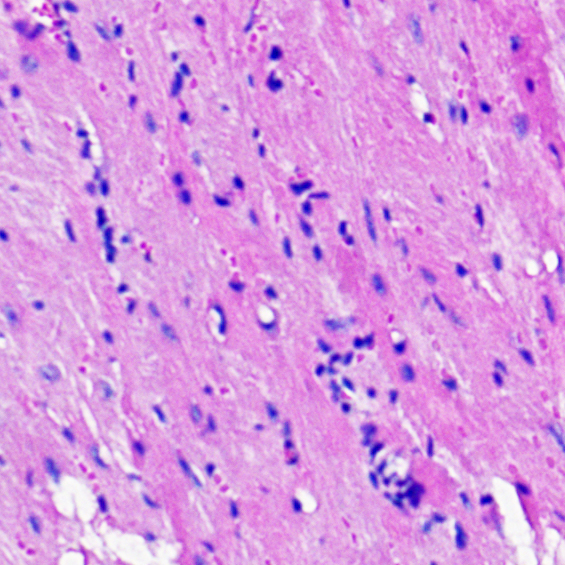

Supplement: Supplementary file 4 [file DataSheet1.zip › Data Sheet 1/Figure 1/Figure 1D/HE/CLP 24h.jpg]

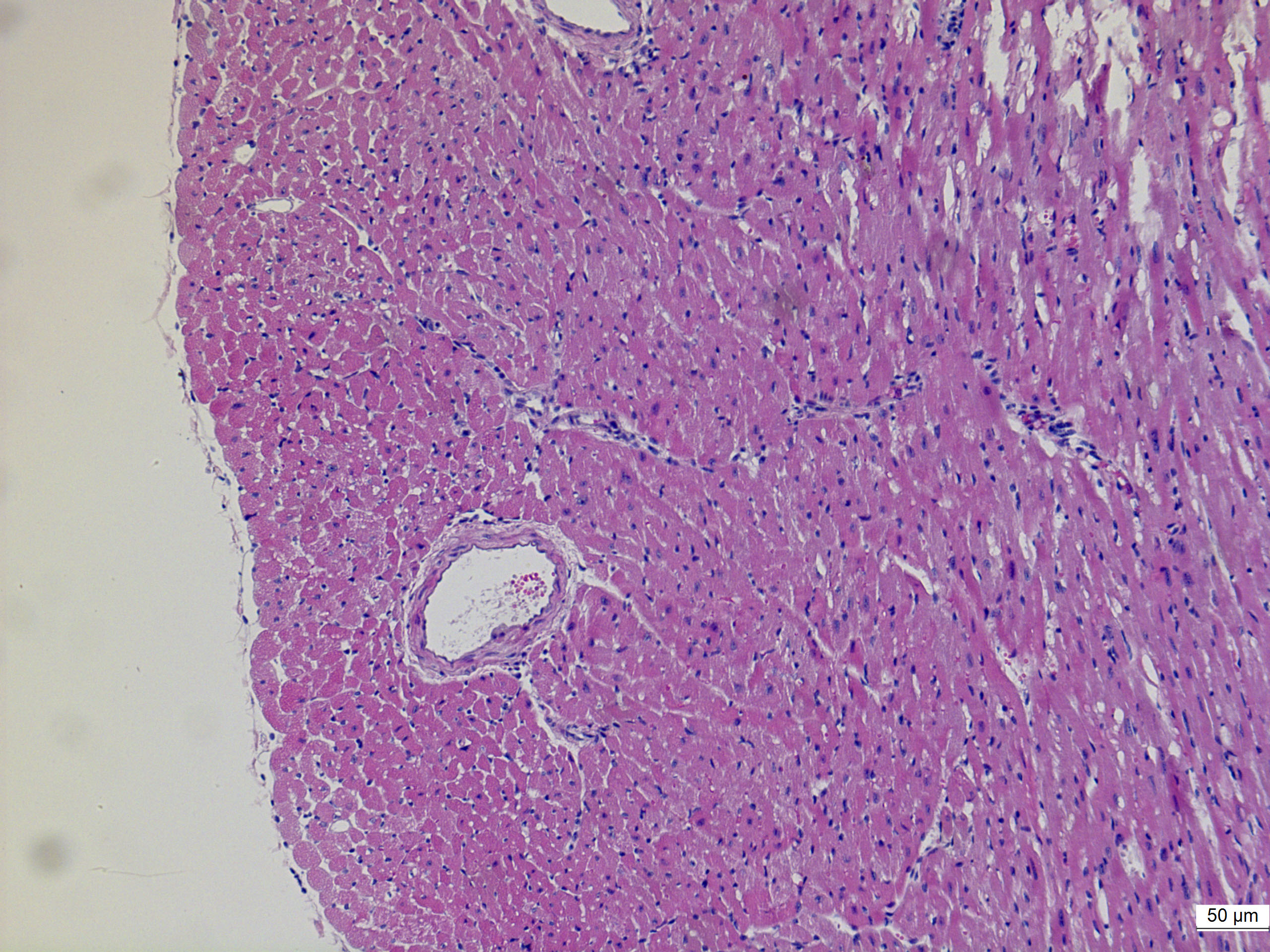

Supplement: Supplementary file 4 [file DataSheet1.zip › Data Sheet 1/Figure 1/Figure 1D/HE/CLP 48h original.jpg]

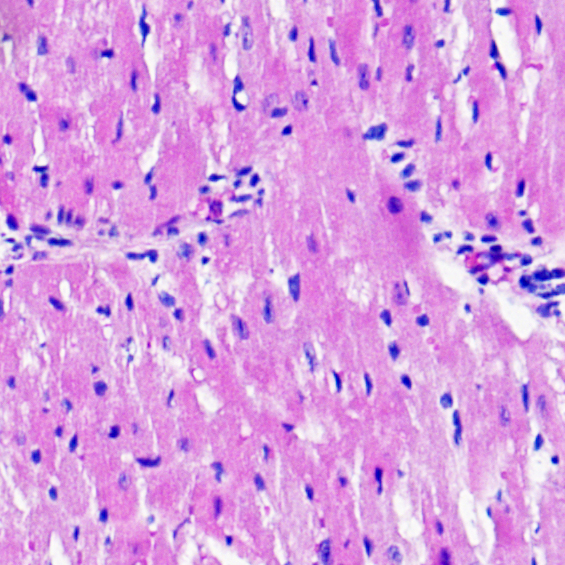

Supplement: Supplementary file 4 [file DataSheet1.zip › Data Sheet 1/Figure 1/Figure 1D/HE/CLP 48h.jpg]

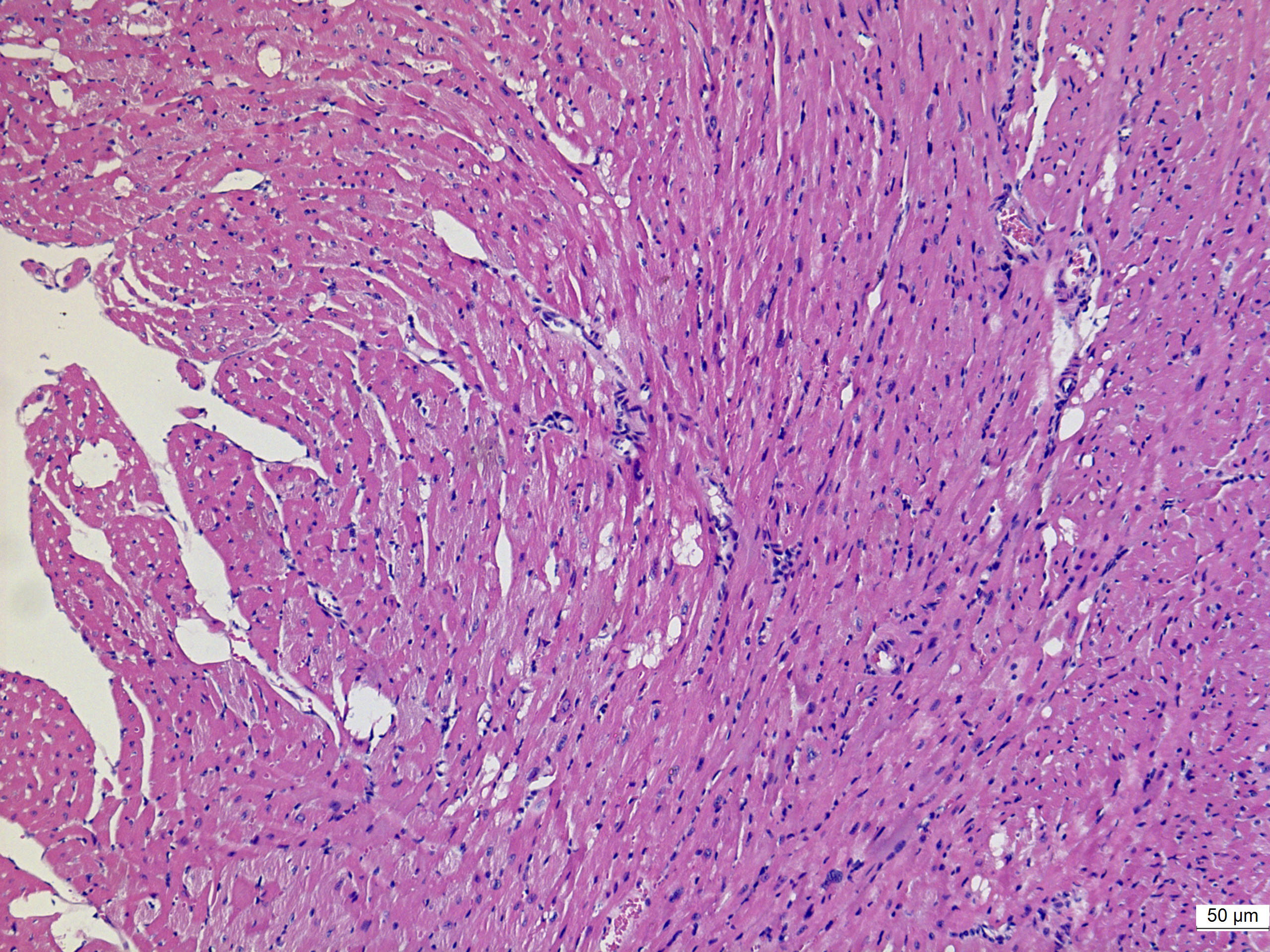

Supplement: Supplementary file 4 [file DataSheet1.zip › Data Sheet 1/Figure 1/Figure 1D/HE/CLP 72h original.jpg]

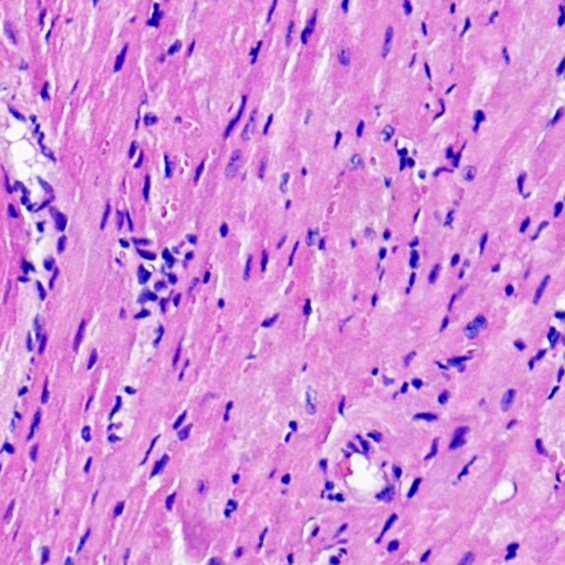

Supplement: Supplementary file 4 [file DataSheet1.zip › Data Sheet 1/Figure 1/Figure 1D/HE/CLP 72h.jpg]

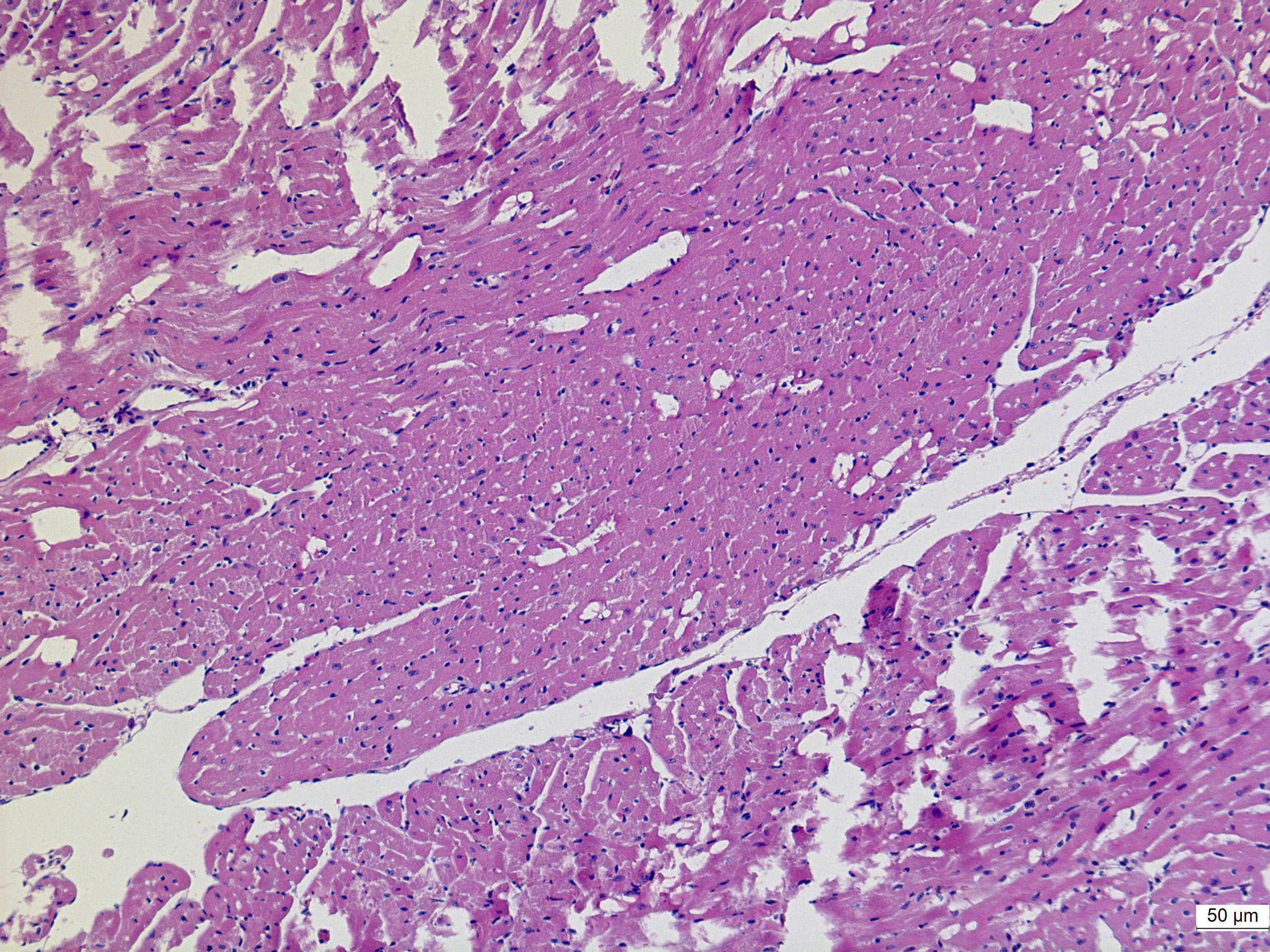

Supplement: Supplementary file 4 [file DataSheet1.zip › Data Sheet 1/Figure 1/Figure 1D/HE/Sham original.jpg]

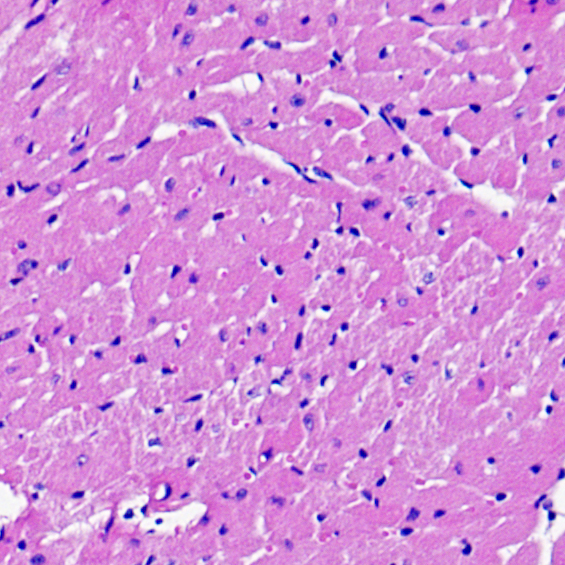

Supplement: Supplementary file 4 [file DataSheet1.zip › Data Sheet 1/Figure 1/Figure 1D/HE/Sham.jpg]

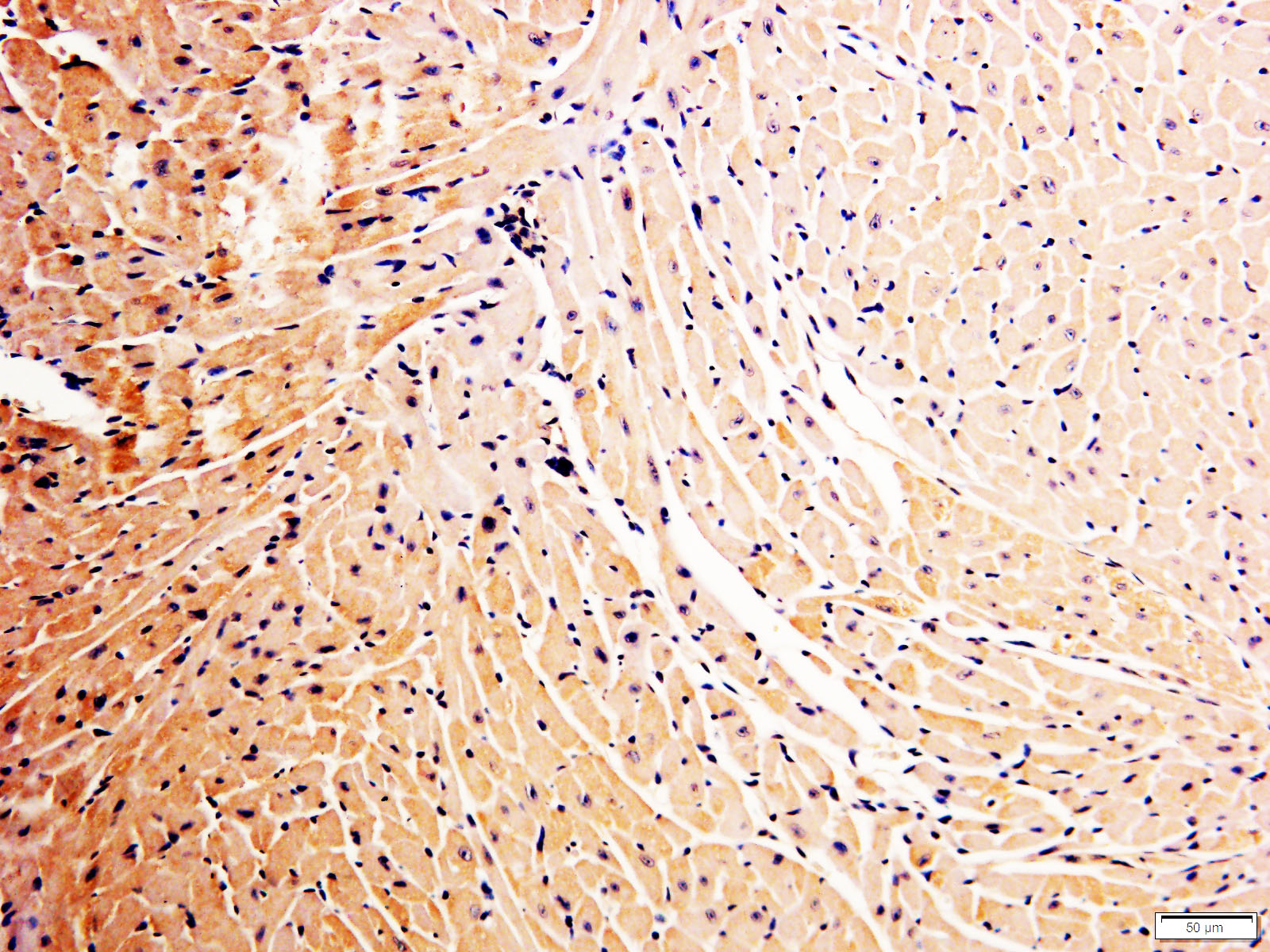

Supplement: Supplementary file 4 [file DataSheet1.zip › Data Sheet 1/Figure 1/Figure 1D/Mac-2/CLP 24H original.jpg]

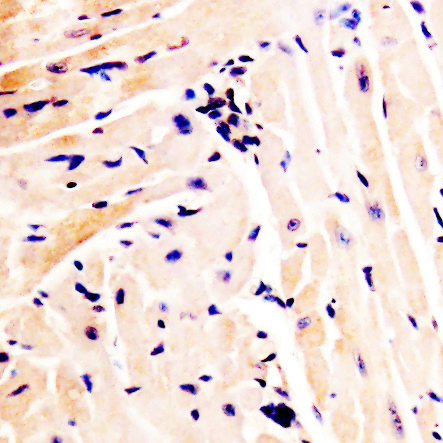

Supplement: Supplementary file 4 [file DataSheet1.zip › Data Sheet 1/Figure 1/Figure 1D/Mac-2/CLP 24H.jpg]

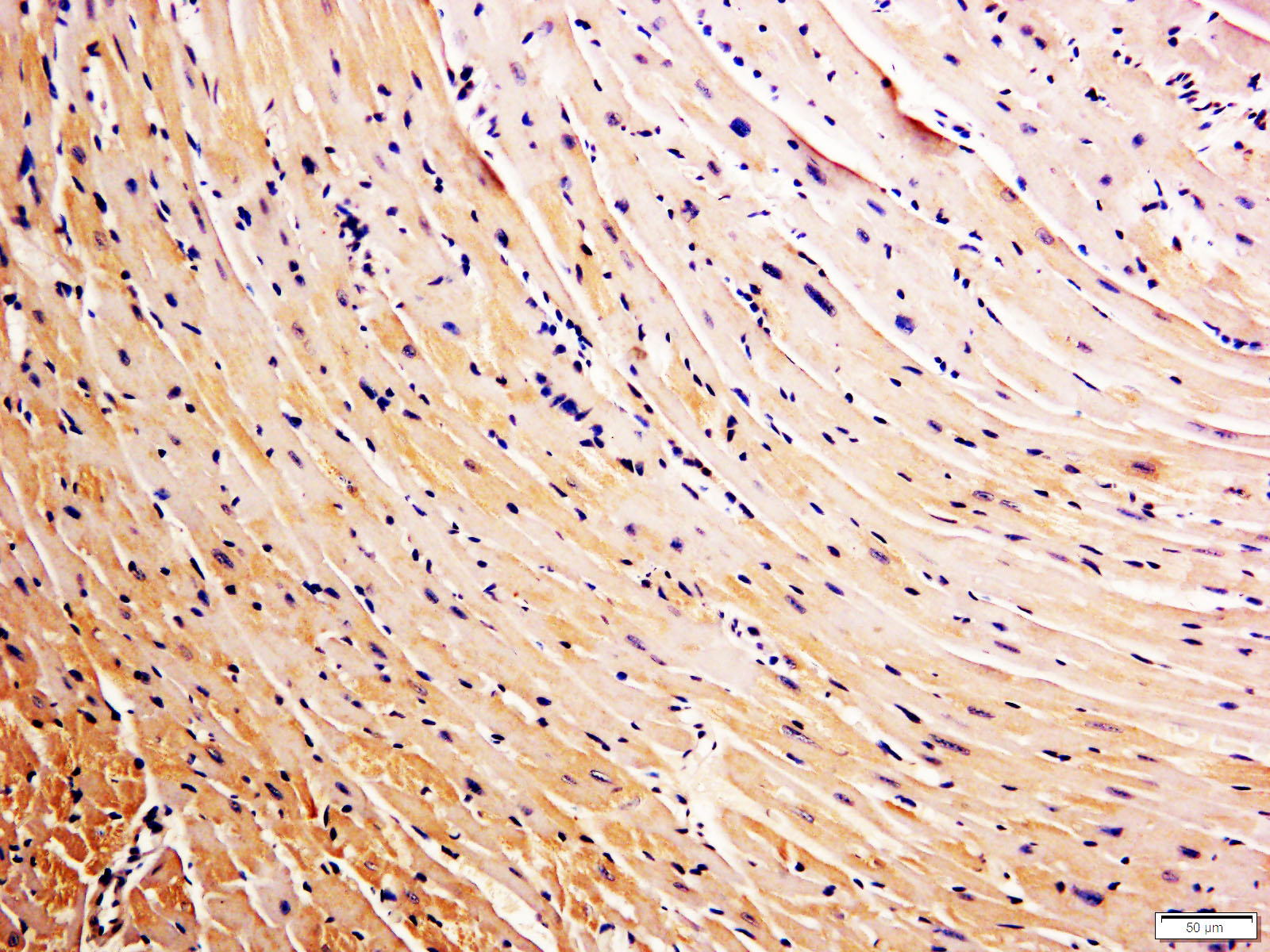

Supplement: Supplementary file 4 [file DataSheet1.zip › Data Sheet 1/Figure 1/Figure 1D/Mac-2/CLP 48H original.jpg]

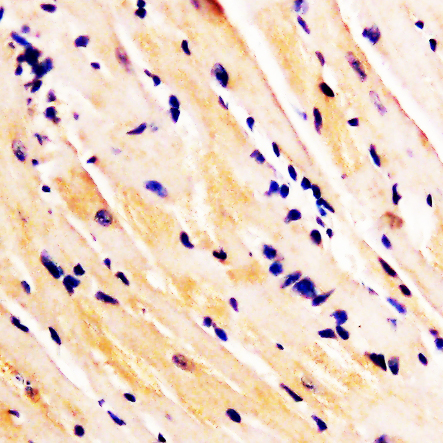

Supplement: Supplementary file 4 [file DataSheet1.zip › Data Sheet 1/Figure 1/Figure 1D/Mac-2/CLP 48H.jpg]

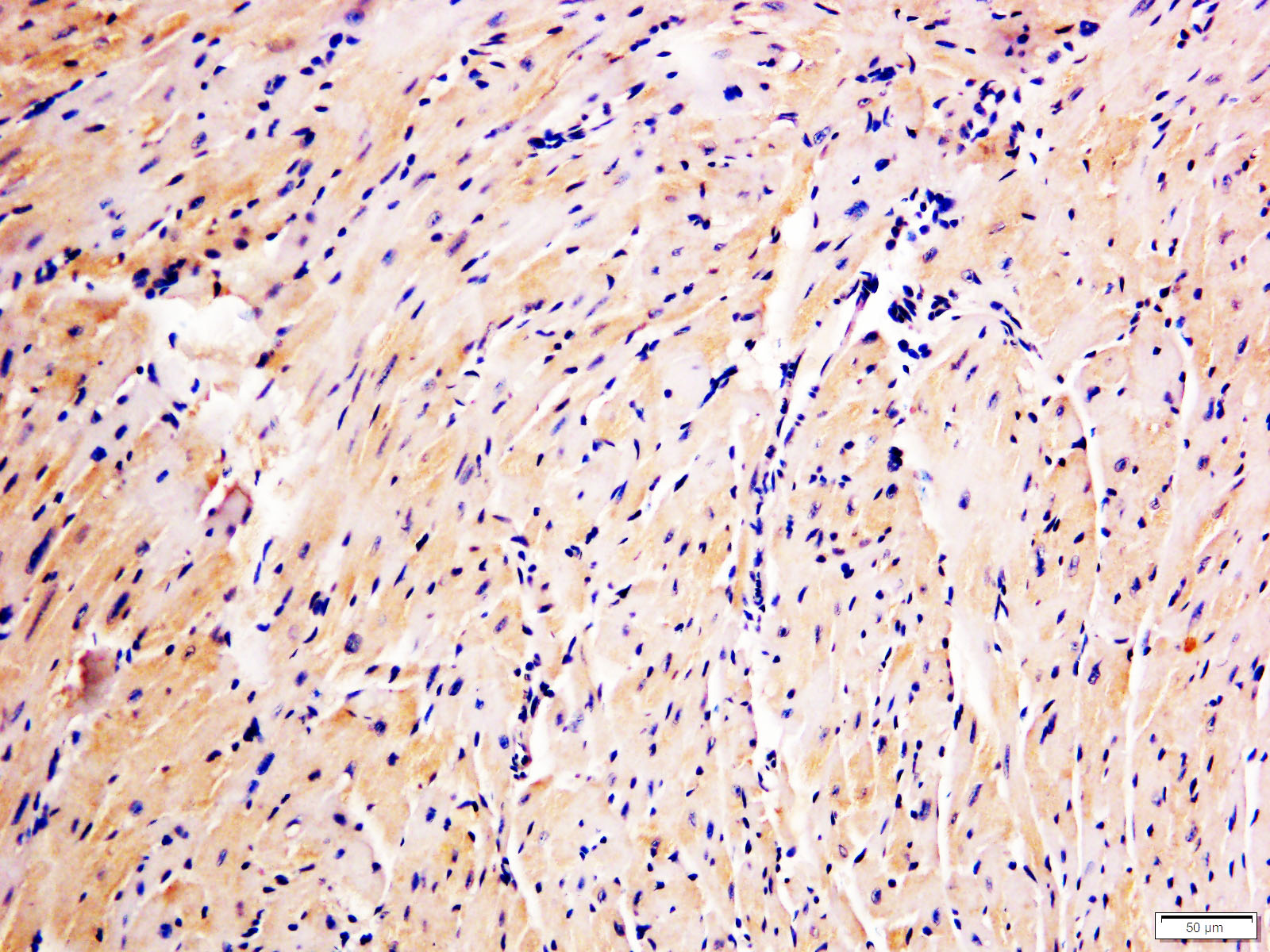

Supplement: Supplementary file 4 [file DataSheet1.zip › Data Sheet 1/Figure 1/Figure 1D/Mac-2/CLP 72H original.jpg]

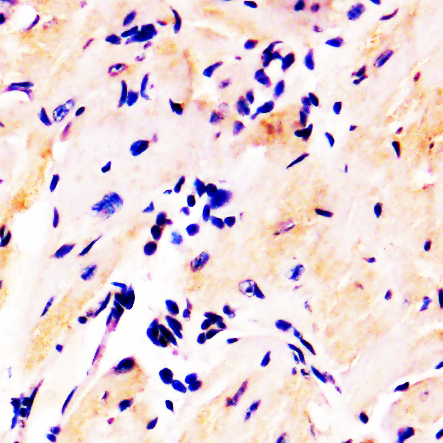

Supplement: Supplementary file 4 [file DataSheet1.zip › Data Sheet 1/Figure 1/Figure 1D/Mac-2/CLP 72H.jpg]

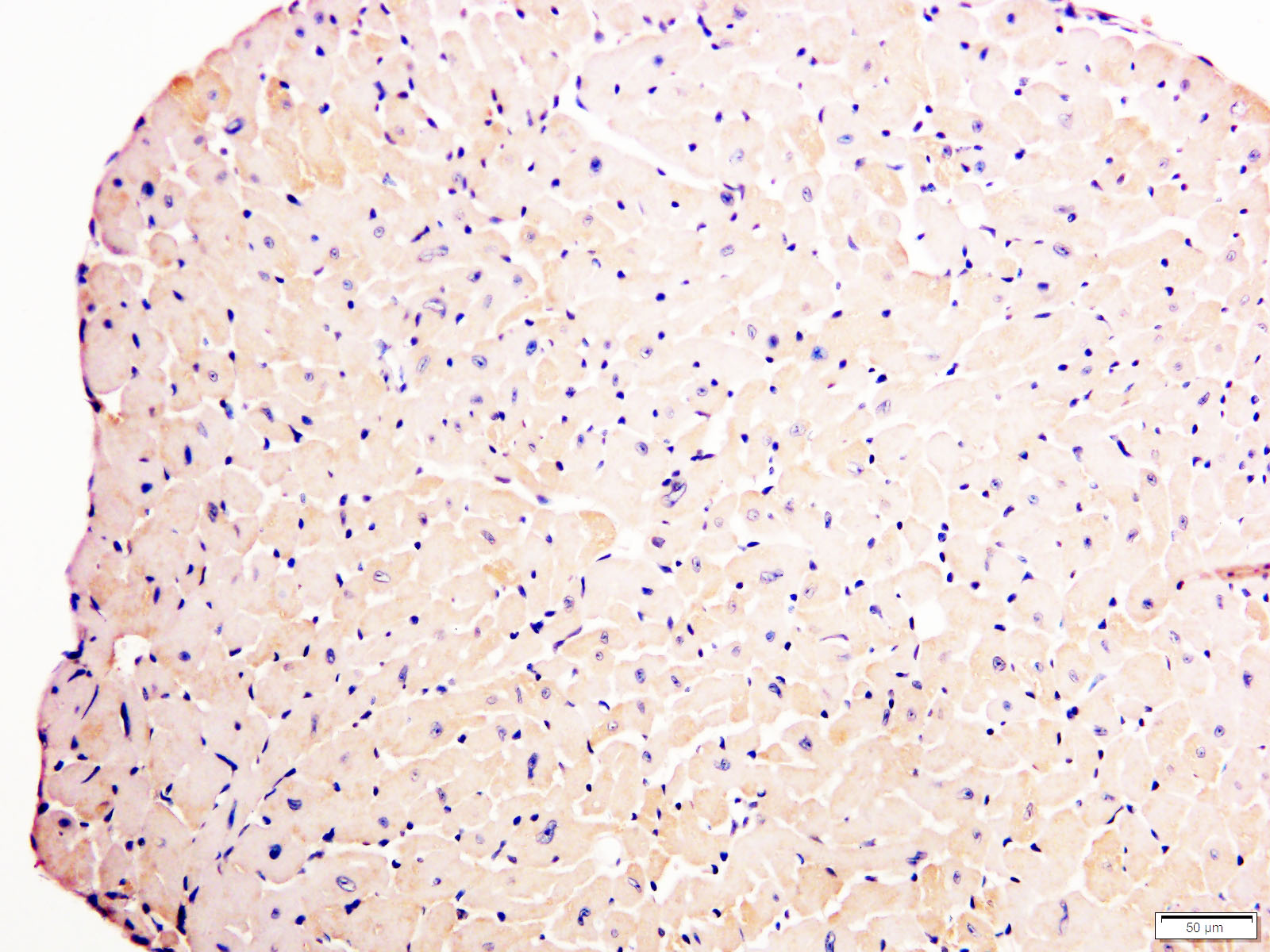

Supplement: Supplementary file 4 [file DataSheet1.zip › Data Sheet 1/Figure 1/Figure 1D/Mac-2/Negative control original.jpg]

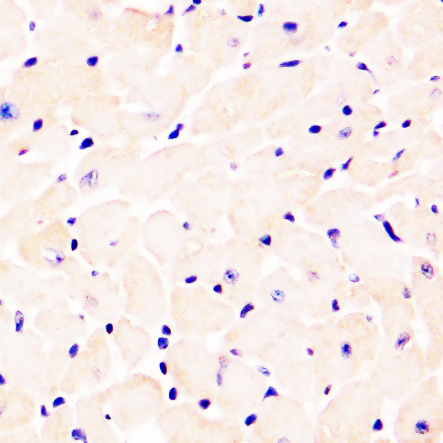

Supplement: Supplementary file 4 [file DataSheet1.zip › Data Sheet 1/Figure 1/Figure 1D/Mac-2/Negative control.jpg]

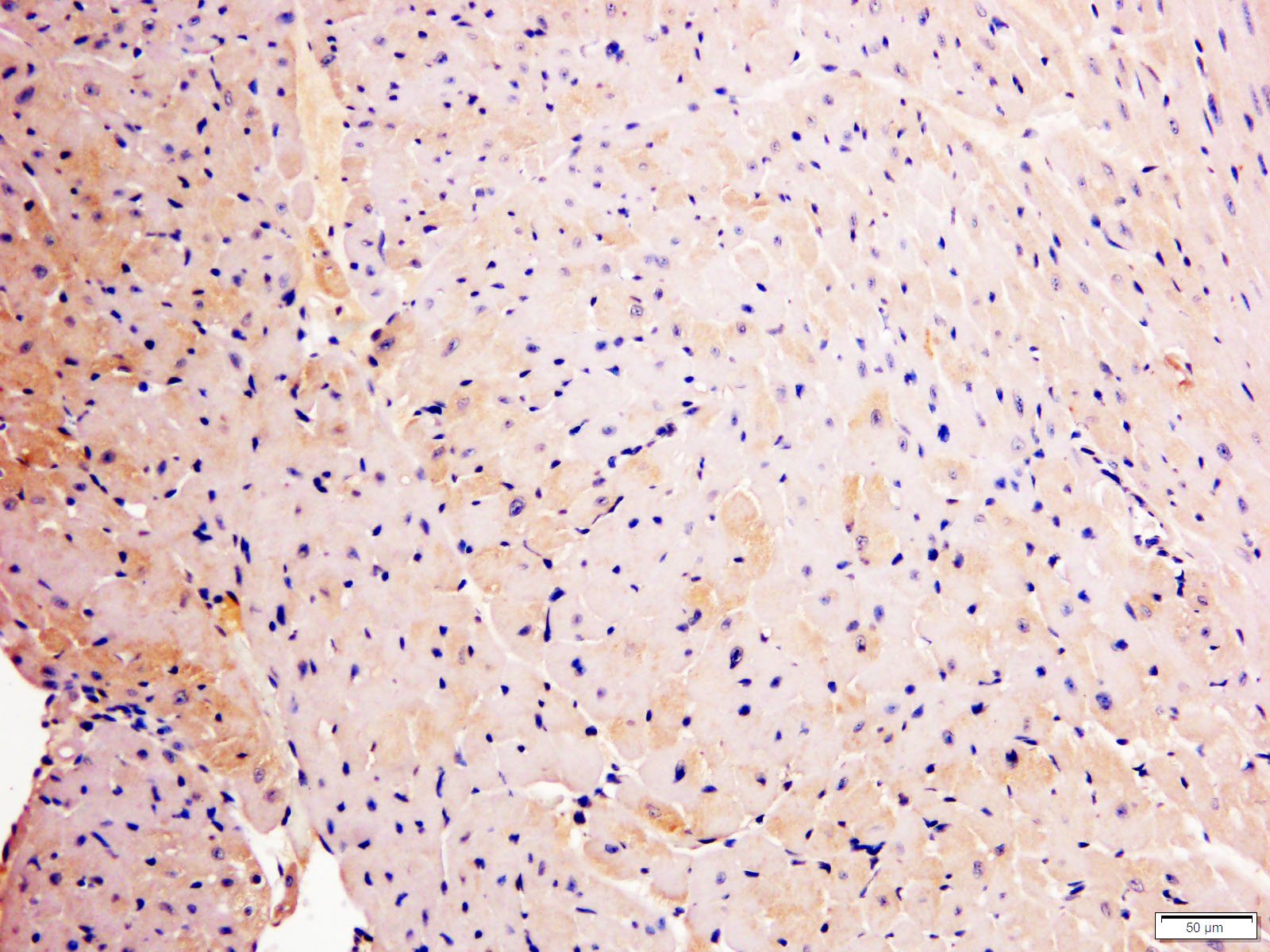

Supplement: Supplementary file 4 [file DataSheet1.zip › Data Sheet 1/Figure 1/Figure 1D/Mac-2/Sham original.jpg]

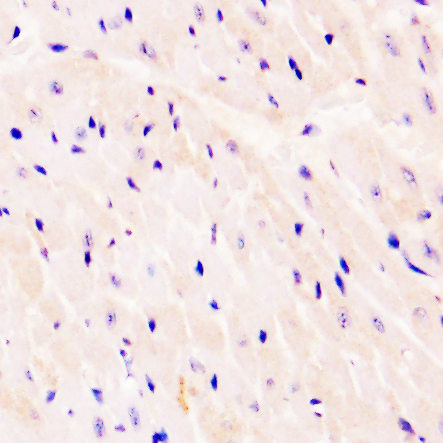

Supplement: Supplementary file 4 [file DataSheet1.zip › Data Sheet 1/Figure 1/Figure 1D/Mac-2/Sham.jpg]

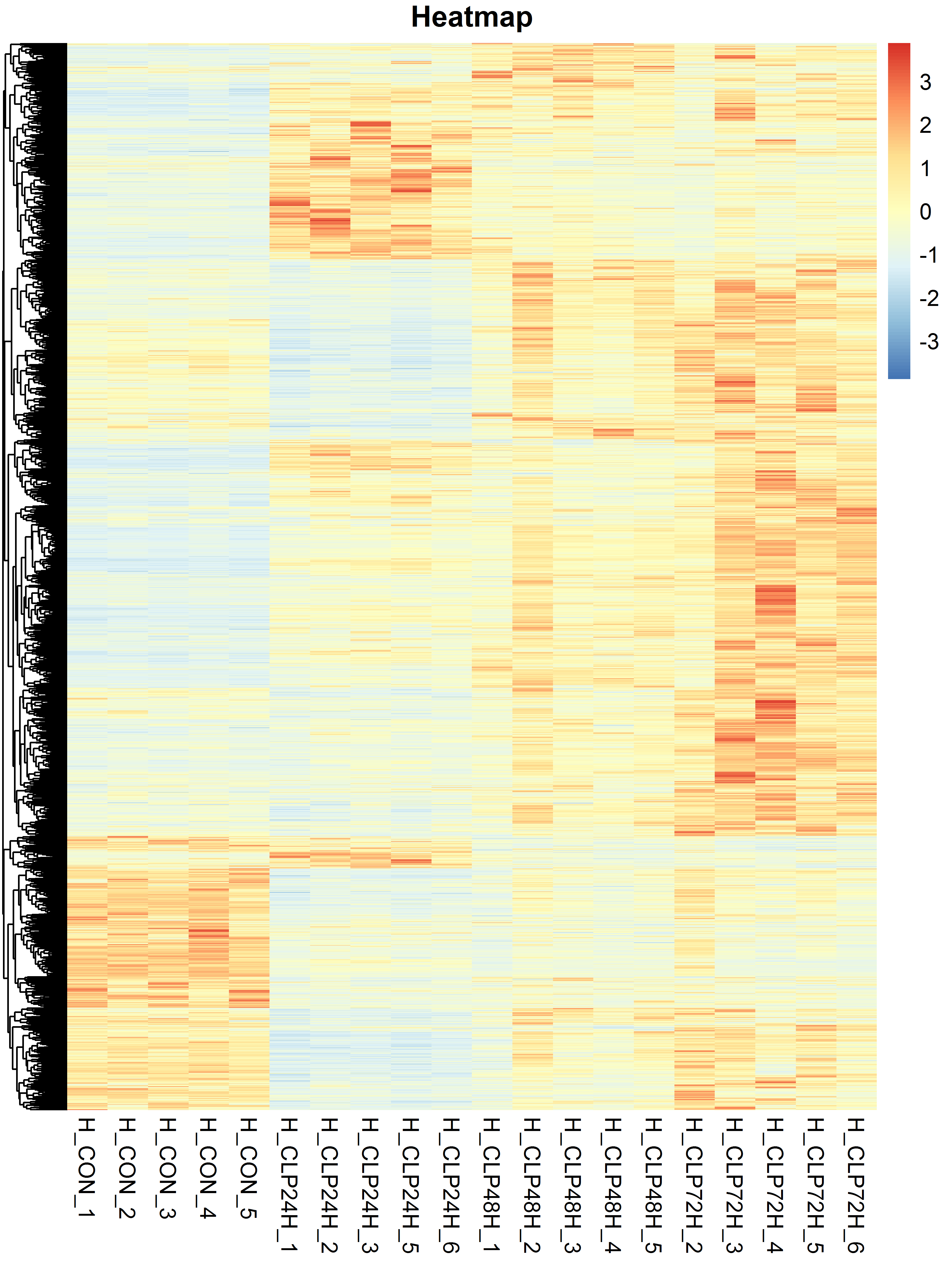

Supplement: Supplementary file 4 [file DataSheet1.zip › Data Sheet 1/Figure 2/Figure 2B/Heatmap.png]

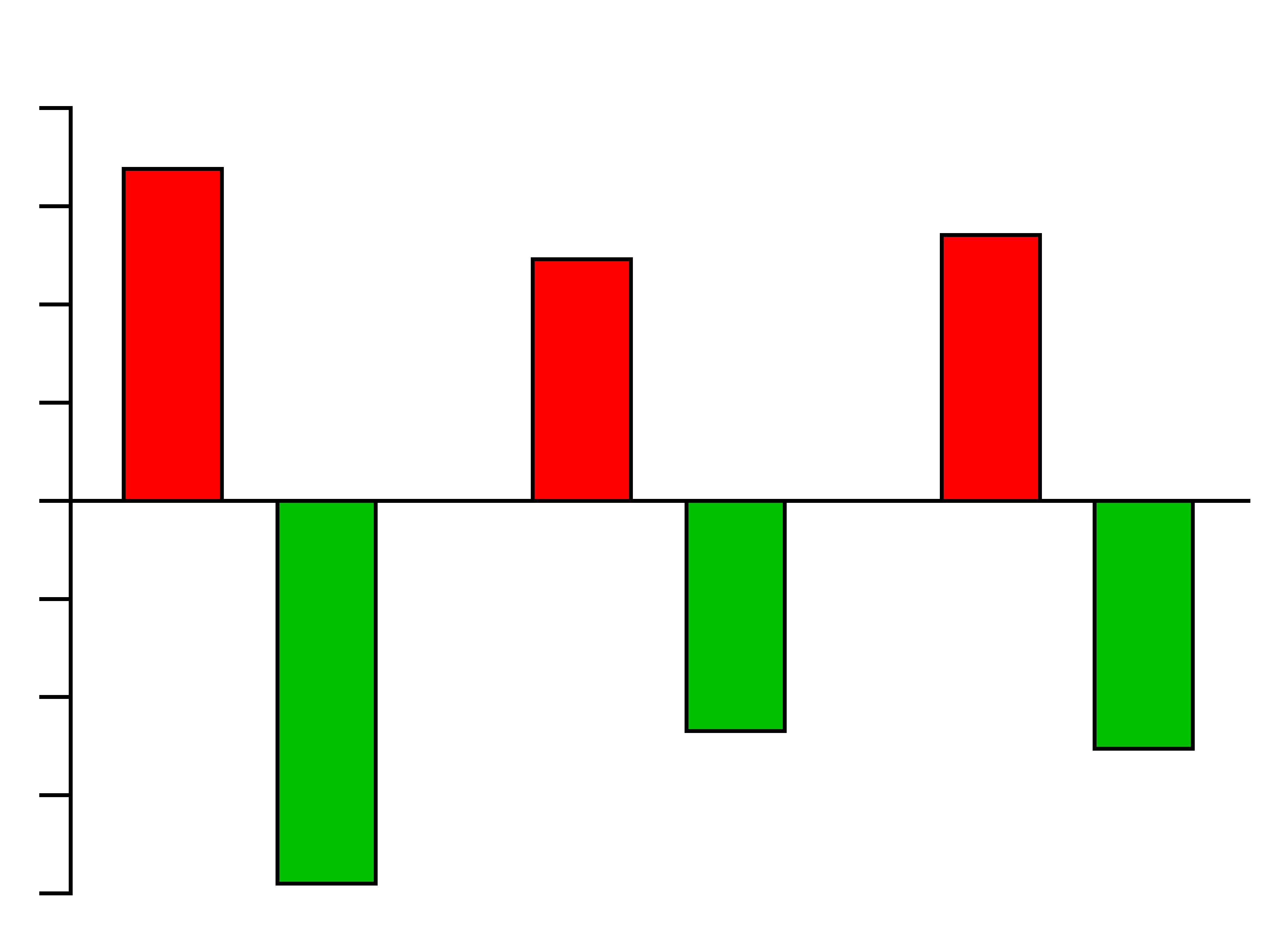

Supplement: Supplementary file 4 [file DataSheet1.zip › Data Sheet 1/Figure 2/Figure 2C/Figure 2C.jpg]

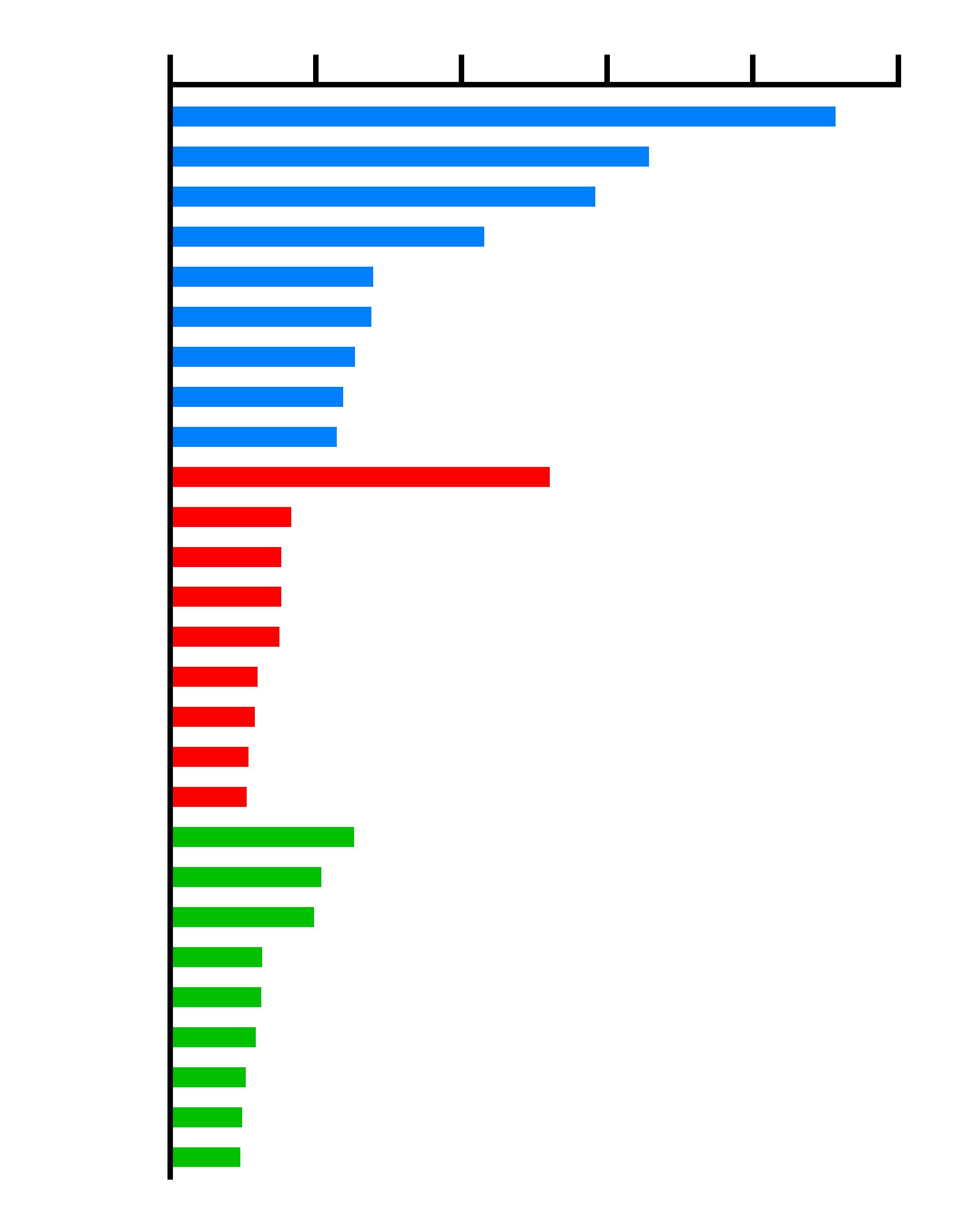

Supplement: Supplementary file 4 [file DataSheet1.zip › Data Sheet 1/Figure 2/Figure 2D/Figure 2D.jpg]

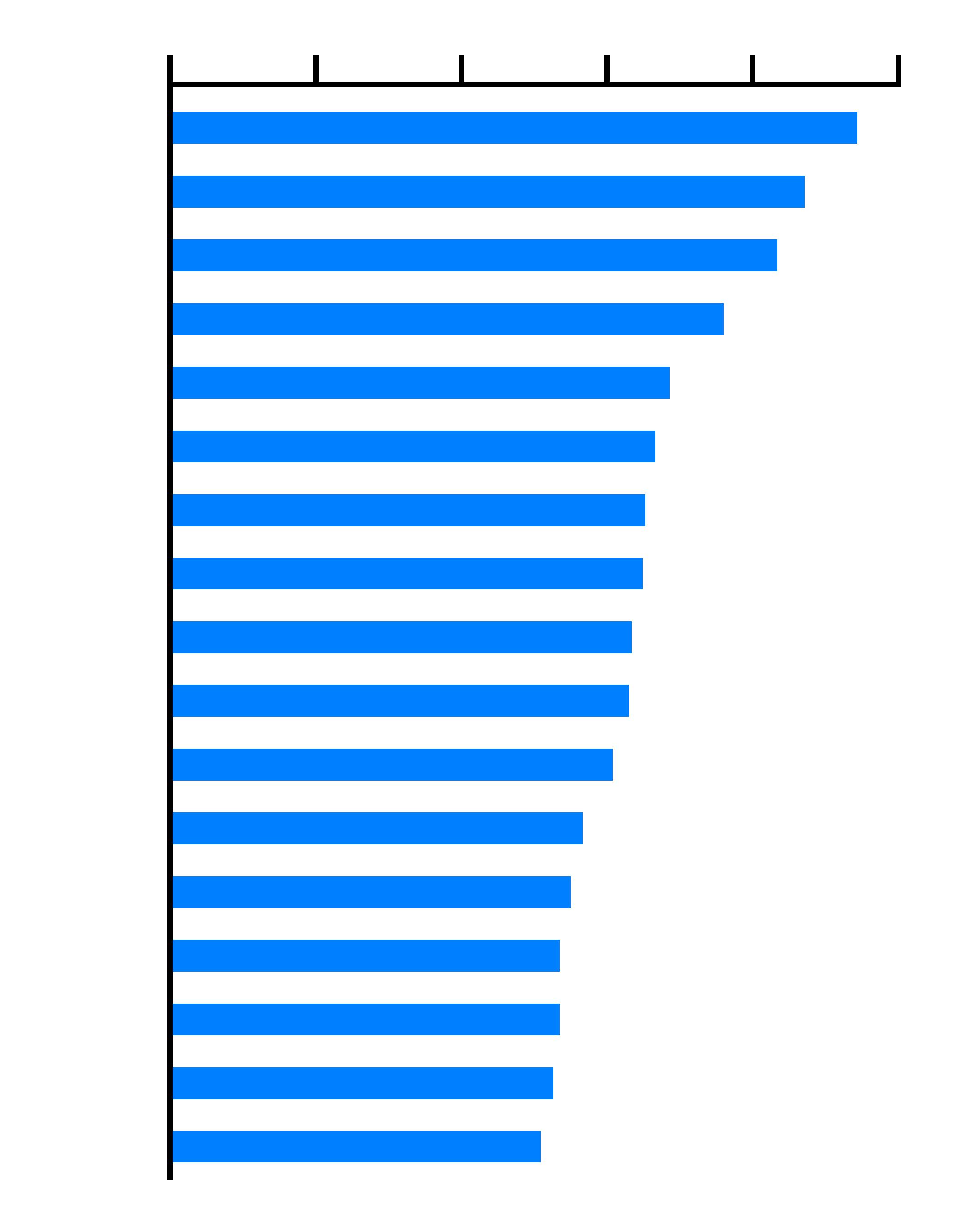

Supplement: Supplementary file 4 [file DataSheet1.zip › Data Sheet 1/Figure 2/Figure 2E/Figure 2E.jpg]

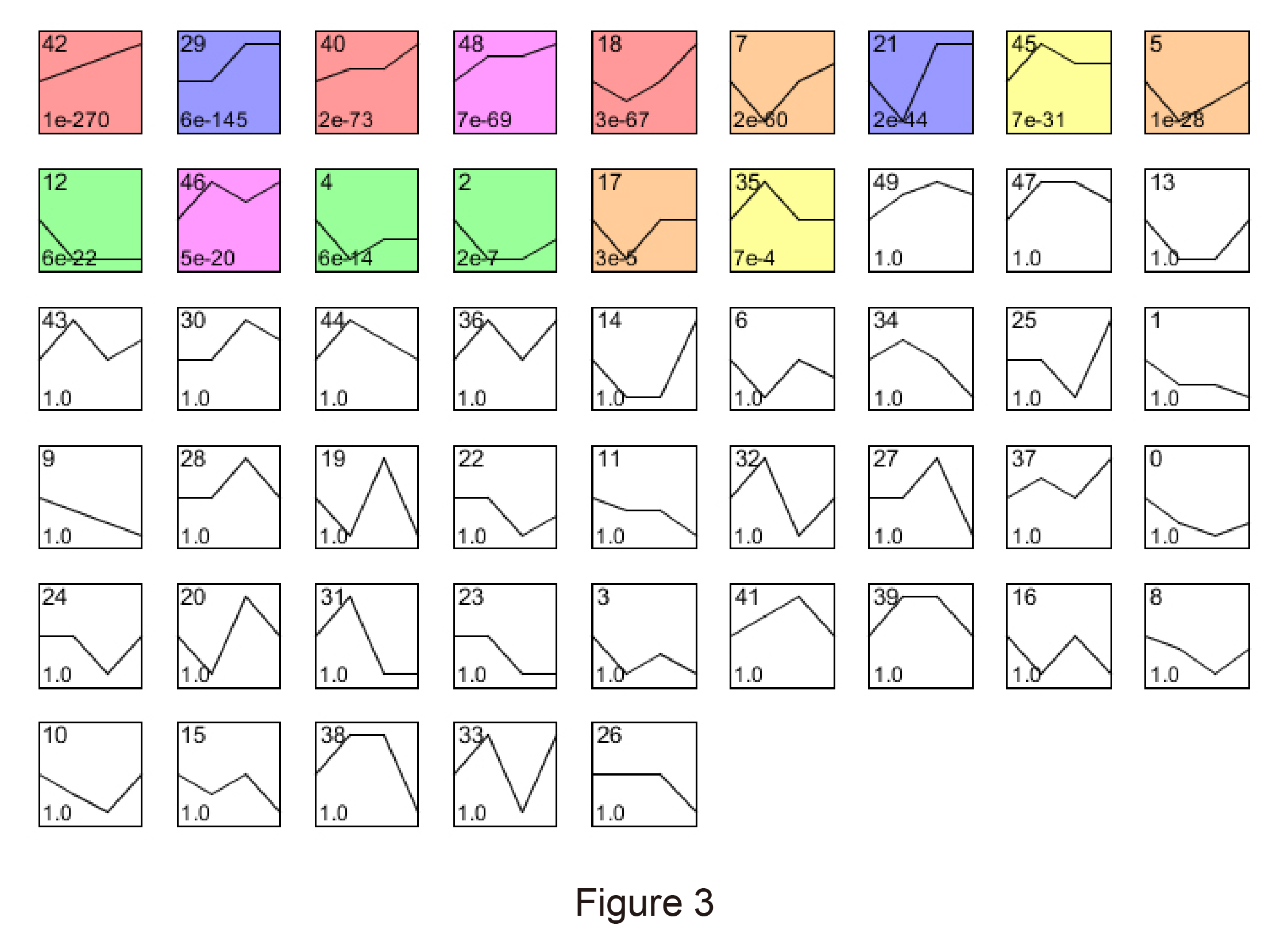

Supplement: Supplementary file 4 [file DataSheet1.zip › Data Sheet 1/Figure 3/Figure 3.jpg]

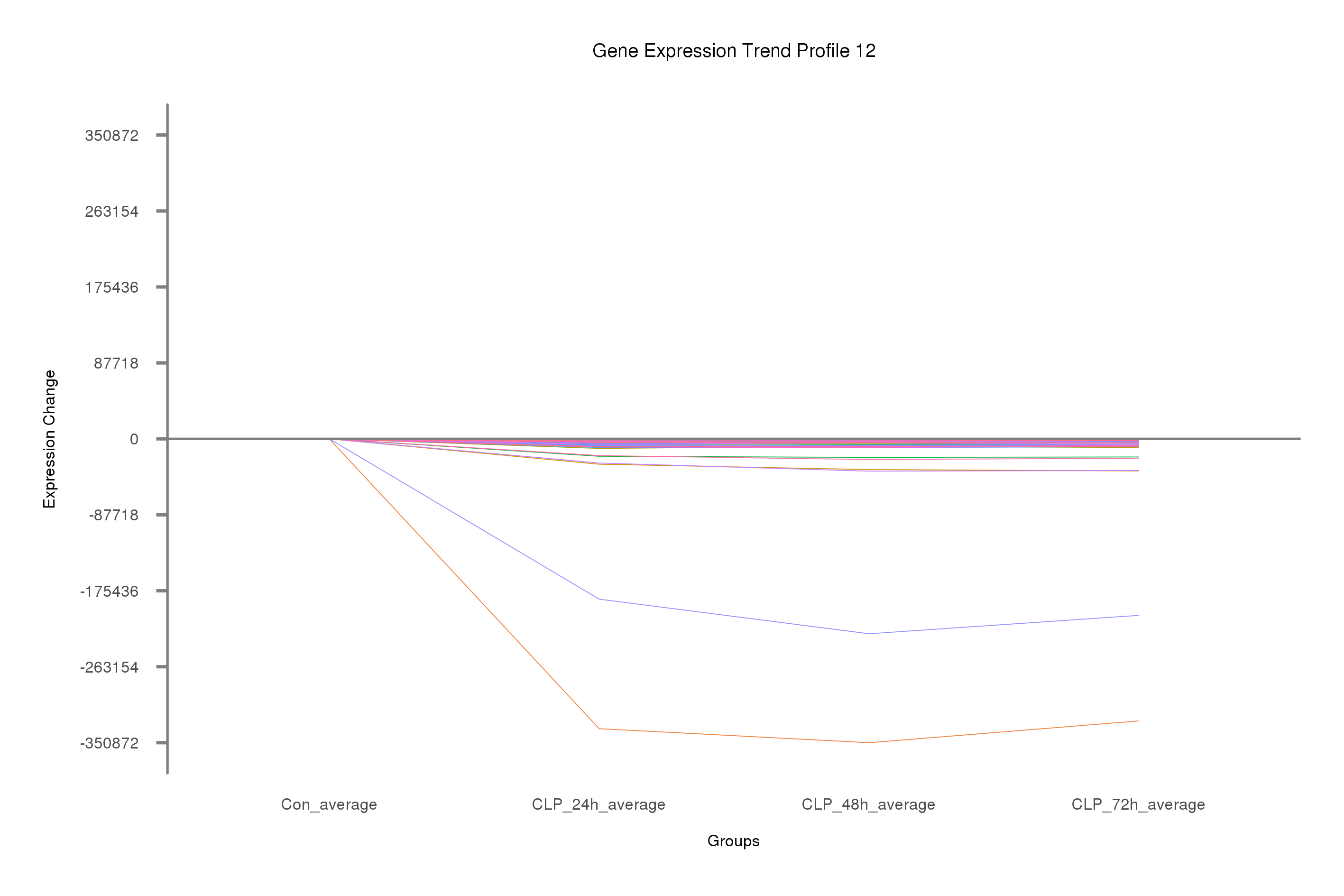

Supplement: Supplementary file 4 [file DataSheet1.zip › Data Sheet 1/Figure 4/12.png]

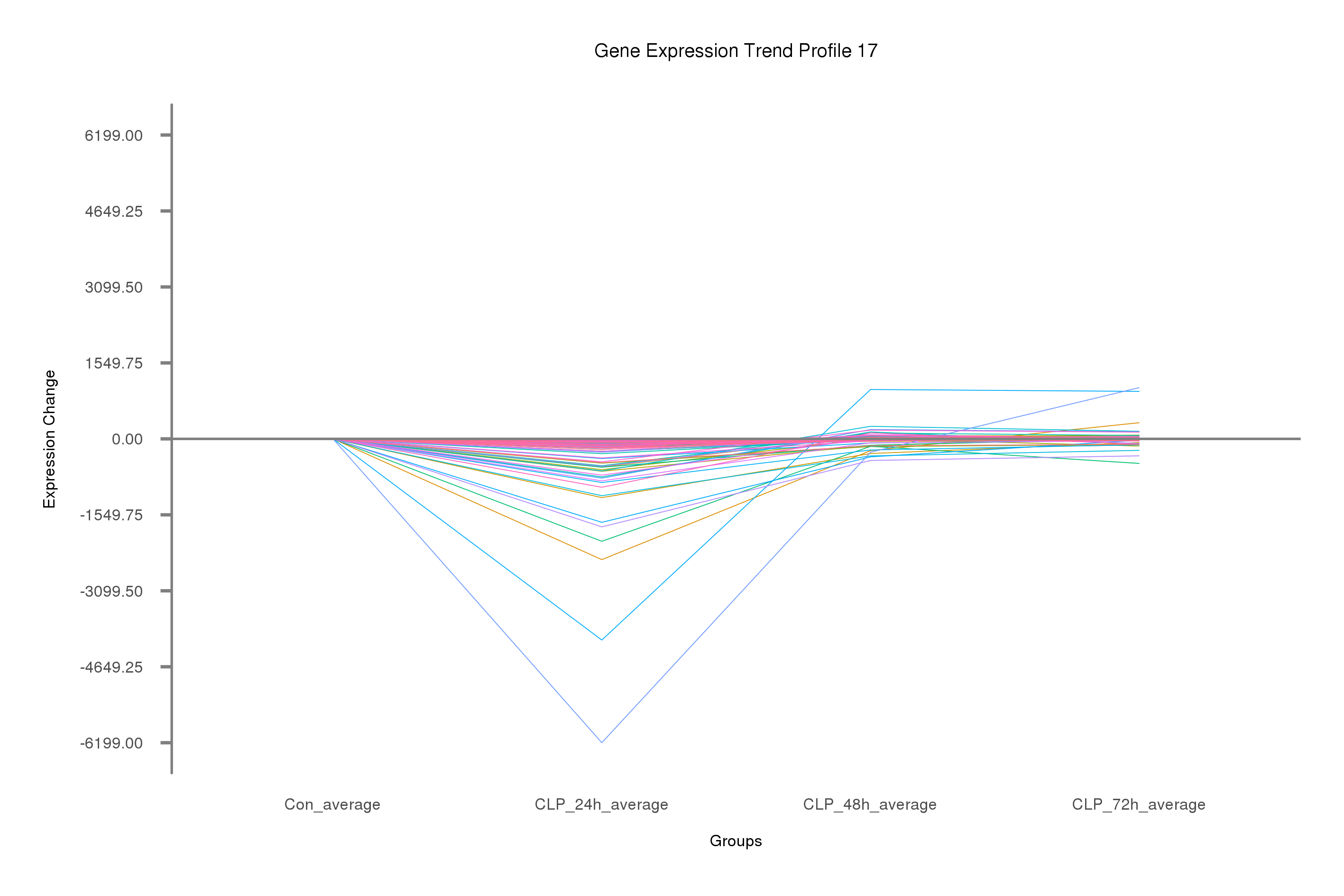

Supplement: Supplementary file 4 [file DataSheet1.zip › Data Sheet 1/Figure 4/17.png]

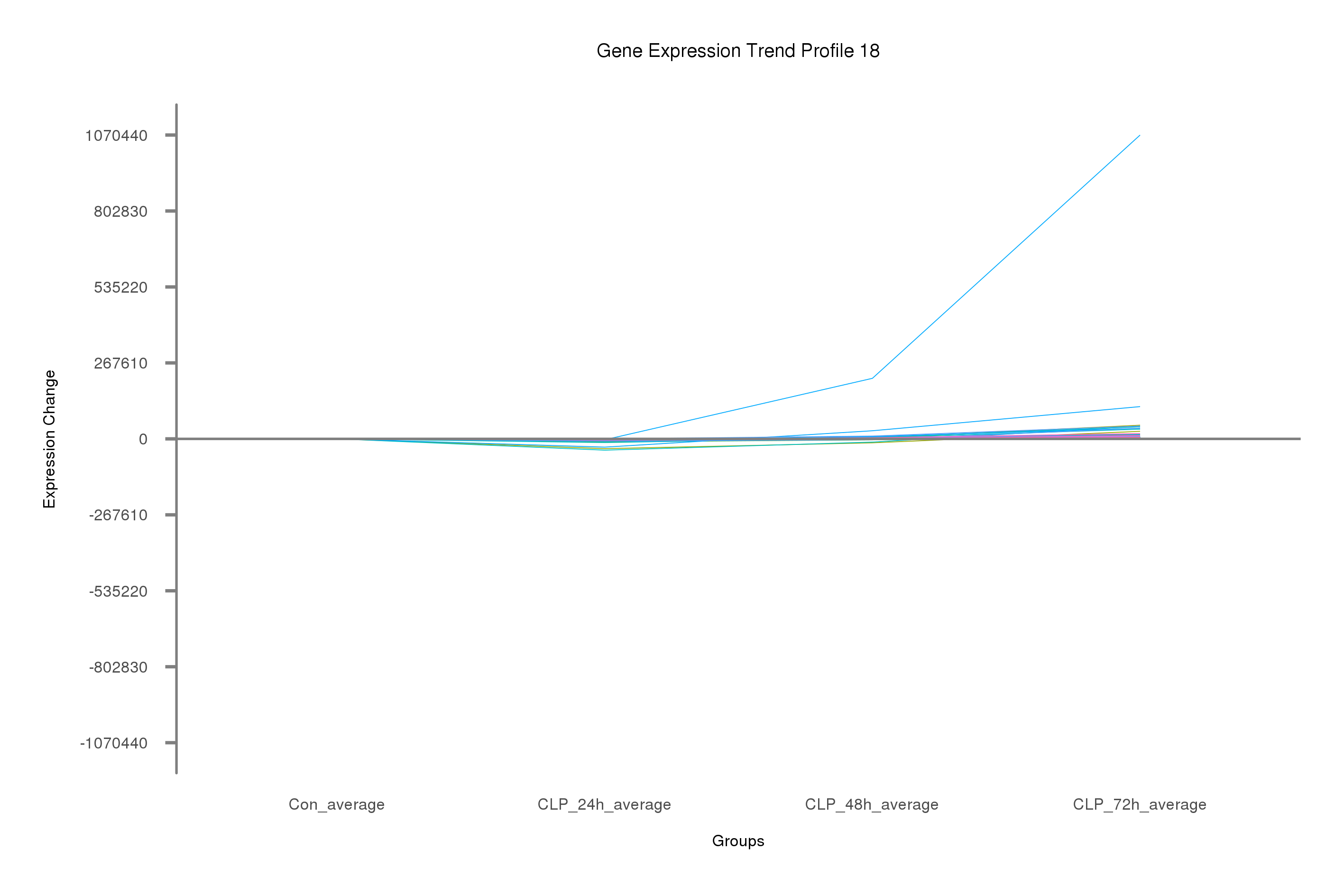

Supplement: Supplementary file 4 [file DataSheet1.zip › Data Sheet 1/Figure 4/18.png]

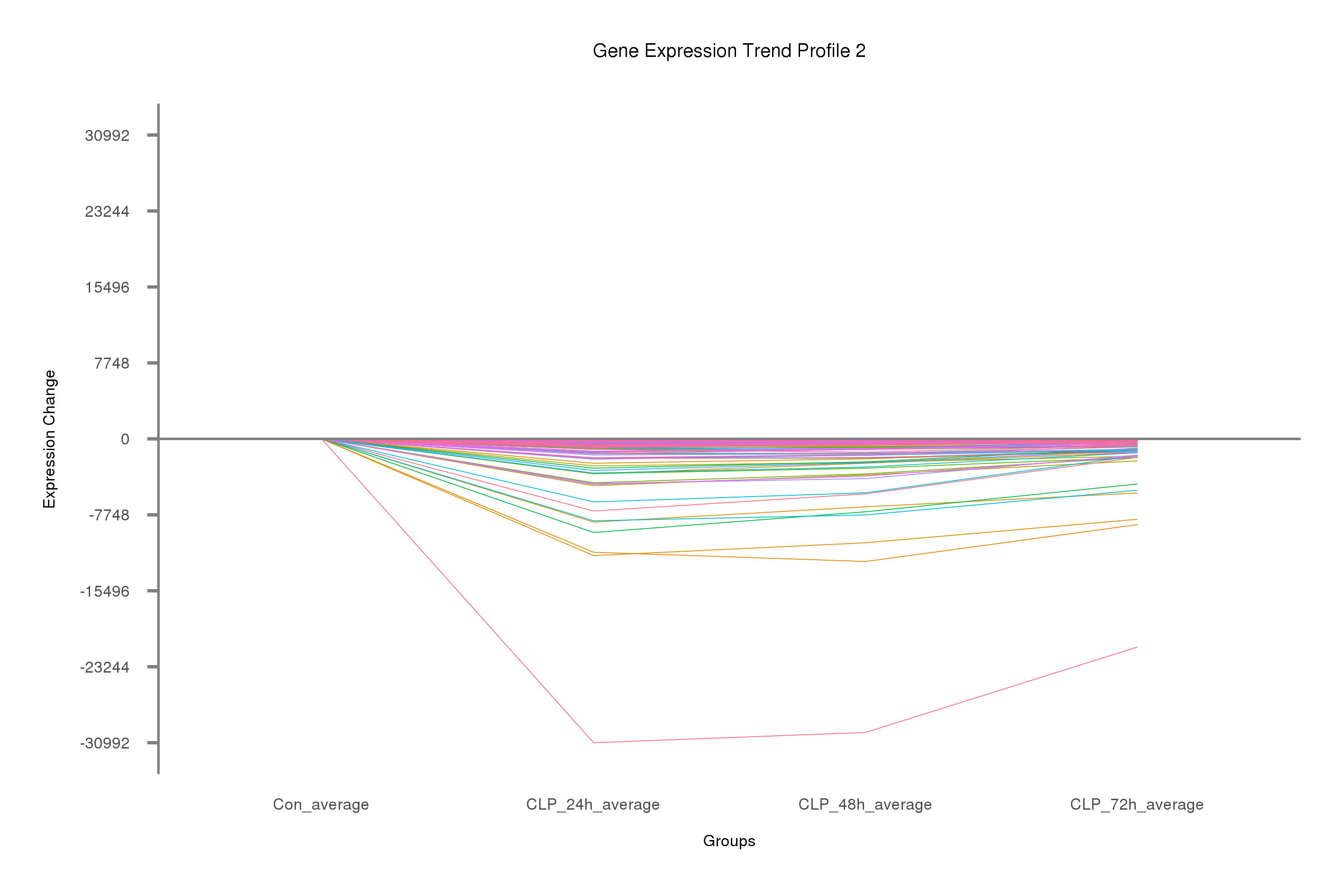

Supplement: Supplementary file 4 [file DataSheet1.zip › Data Sheet 1/Figure 4/2.png]

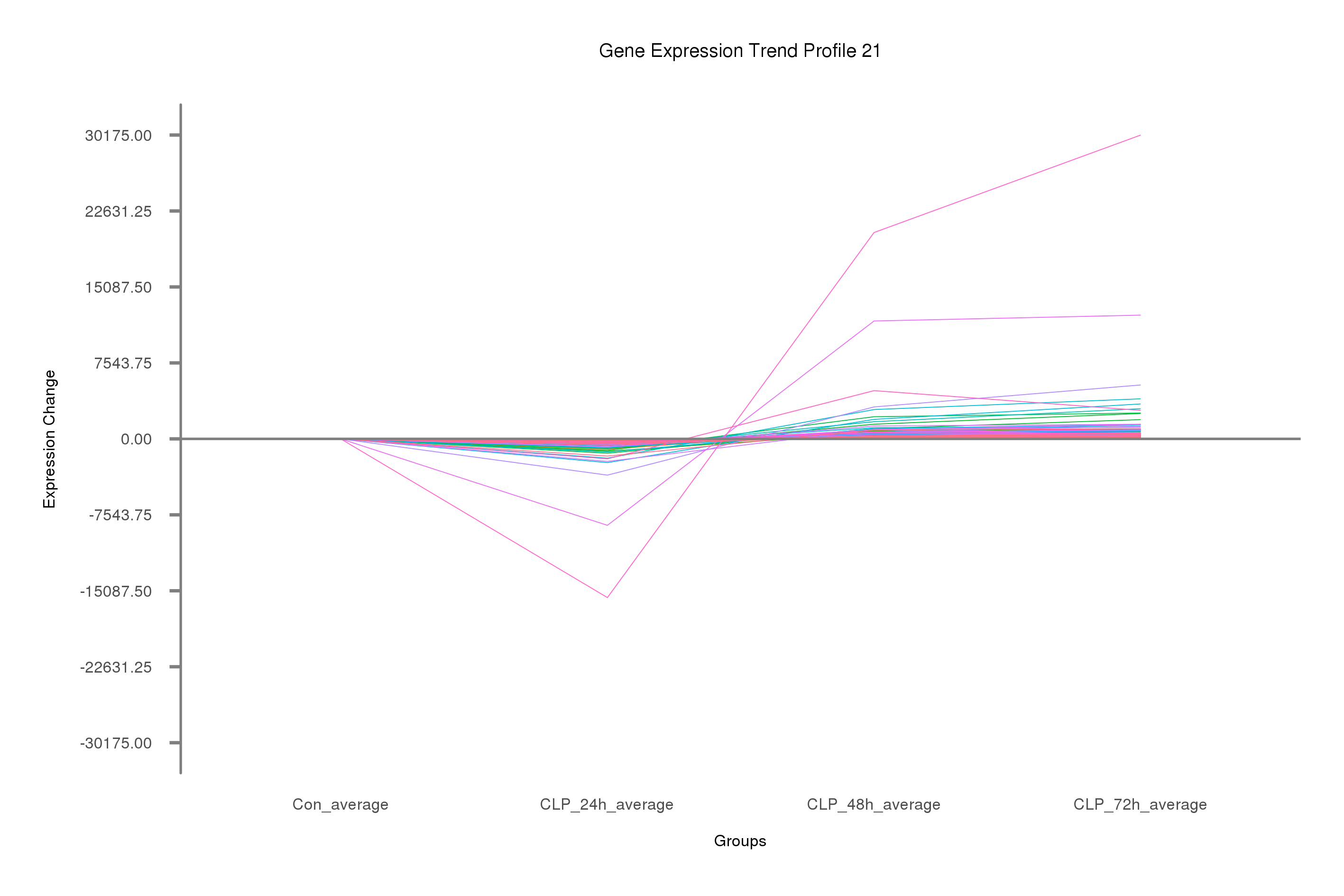

Supplement: Supplementary file 4 [file DataSheet1.zip › Data Sheet 1/Figure 4/21.png]

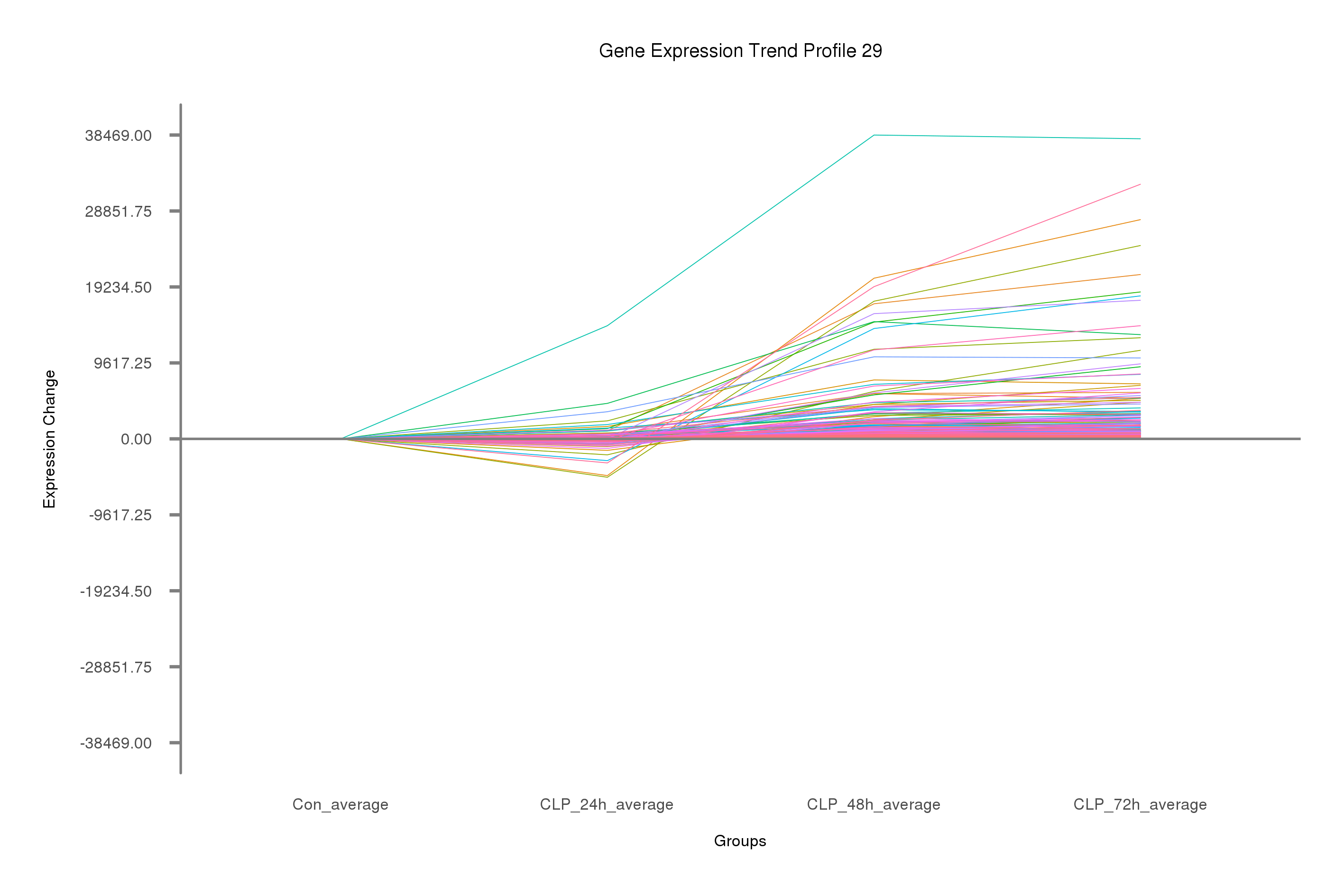

Supplement: Supplementary file 4 [file DataSheet1.zip › Data Sheet 1/Figure 4/29.png]

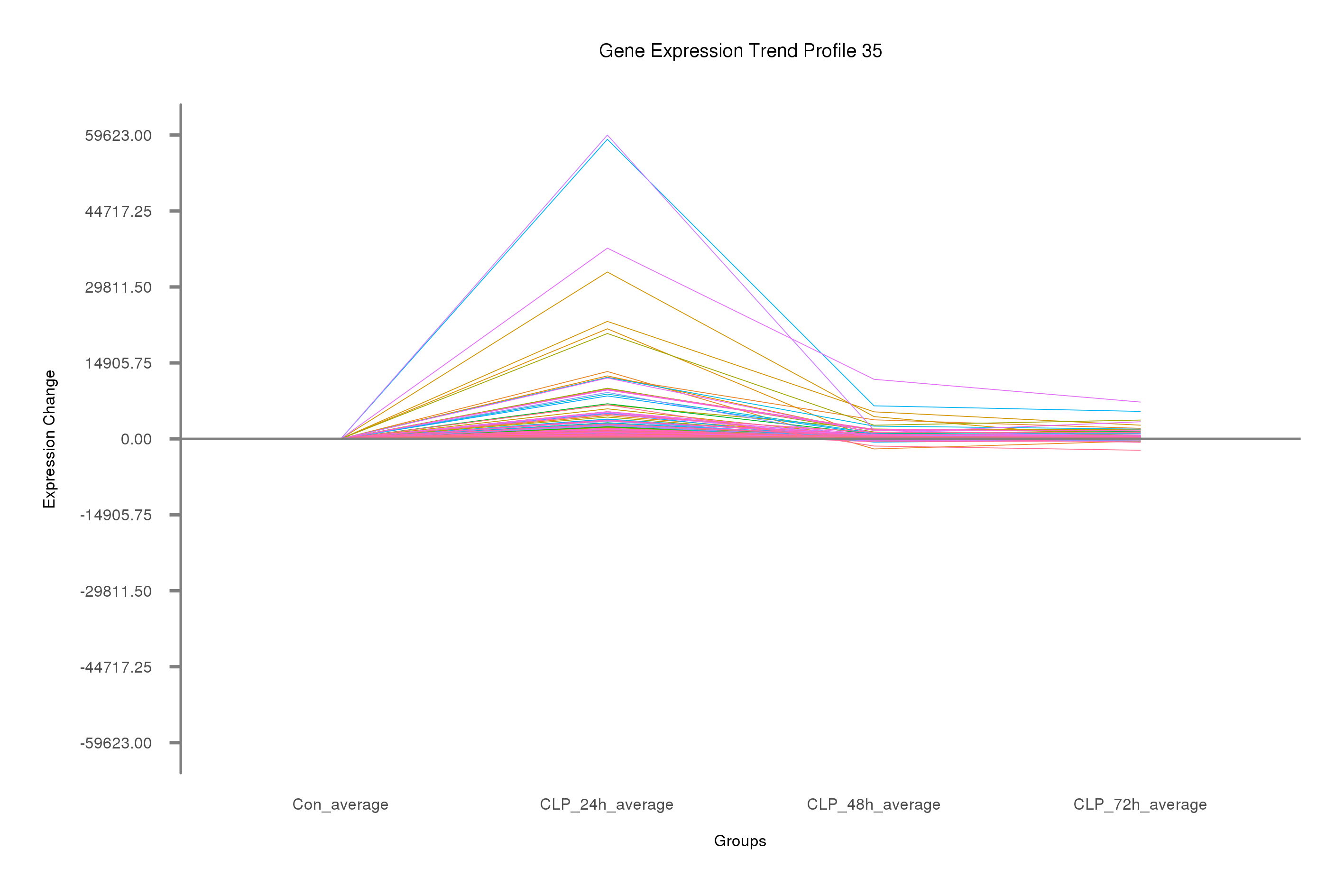

Supplement: Supplementary file 4 [file DataSheet1.zip › Data Sheet 1/Figure 4/35.png]

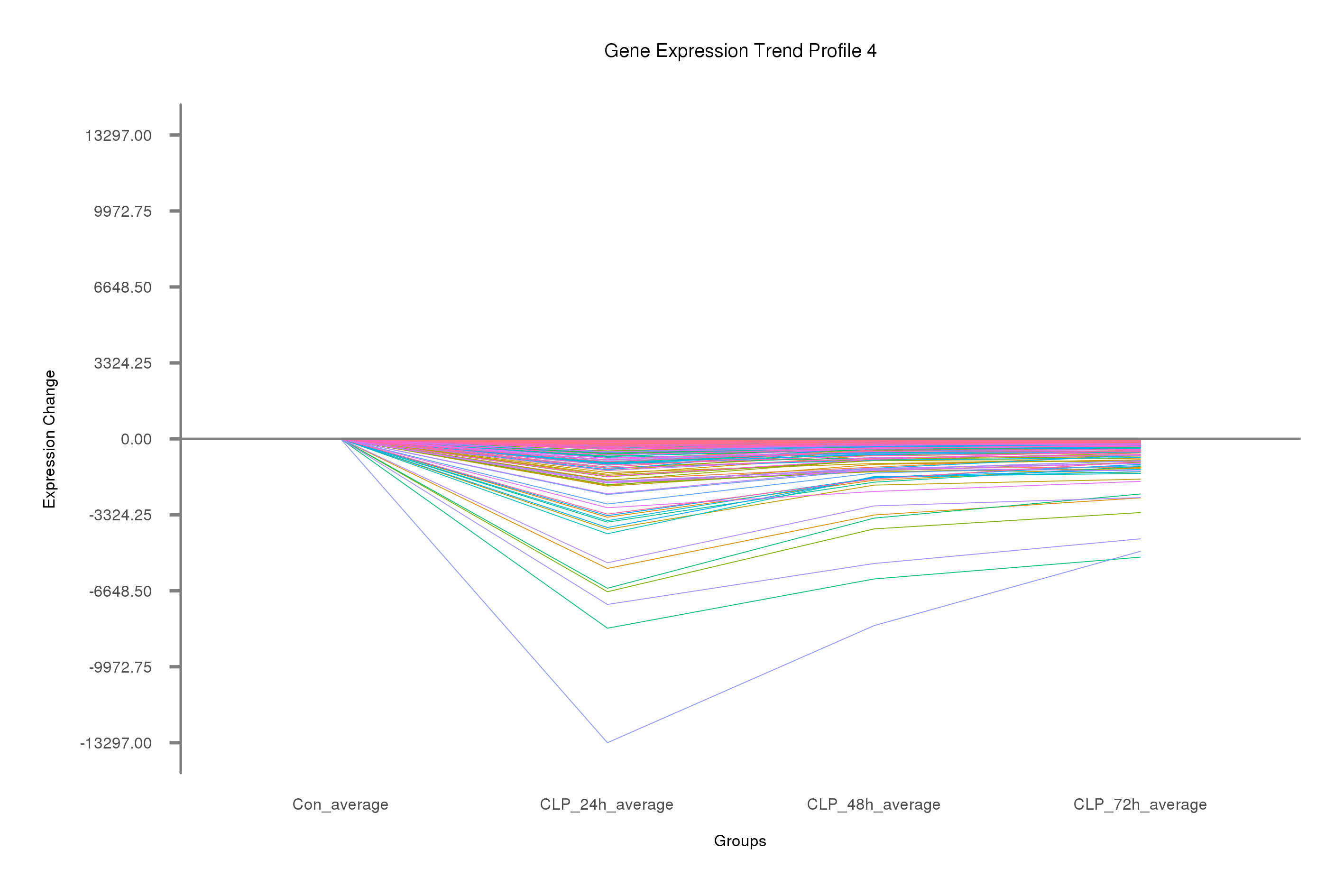

Supplement: Supplementary file 4 [file DataSheet1.zip › Data Sheet 1/Figure 4/4.png]

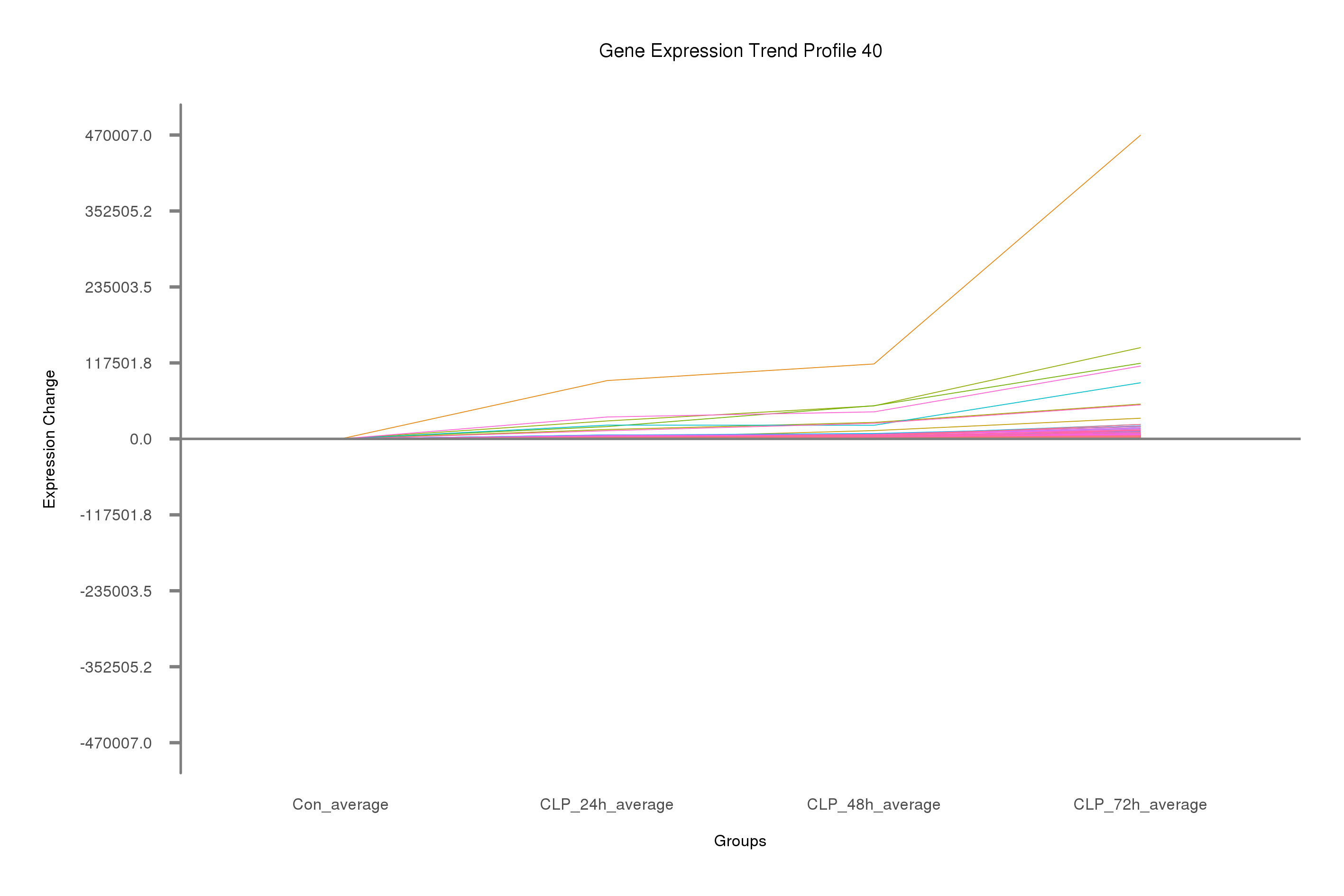

Supplement: Supplementary file 4 [file DataSheet1.zip › Data Sheet 1/Figure 4/40.png]

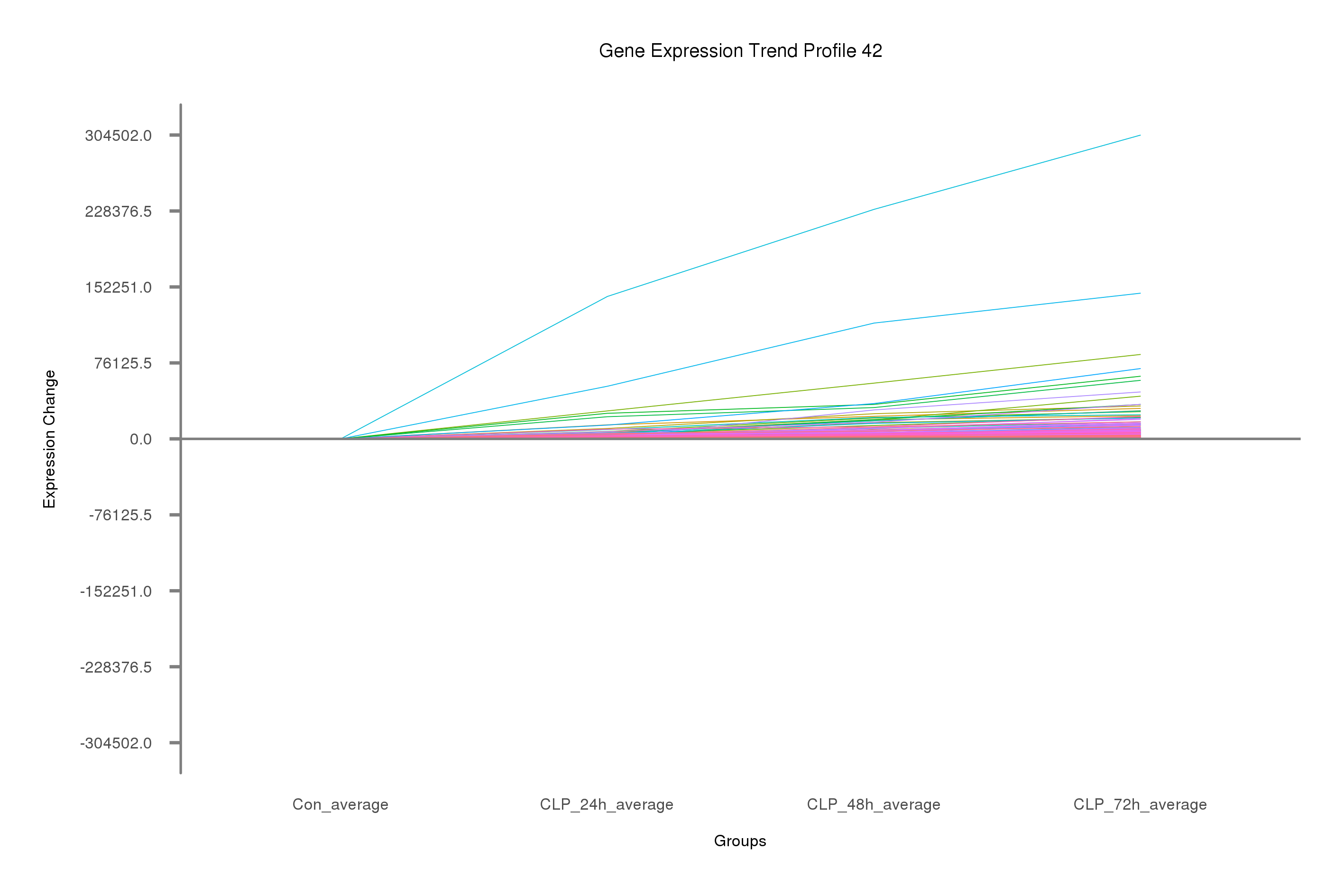

Supplement: Supplementary file 4 [file DataSheet1.zip › Data Sheet 1/Figure 4/42.png]

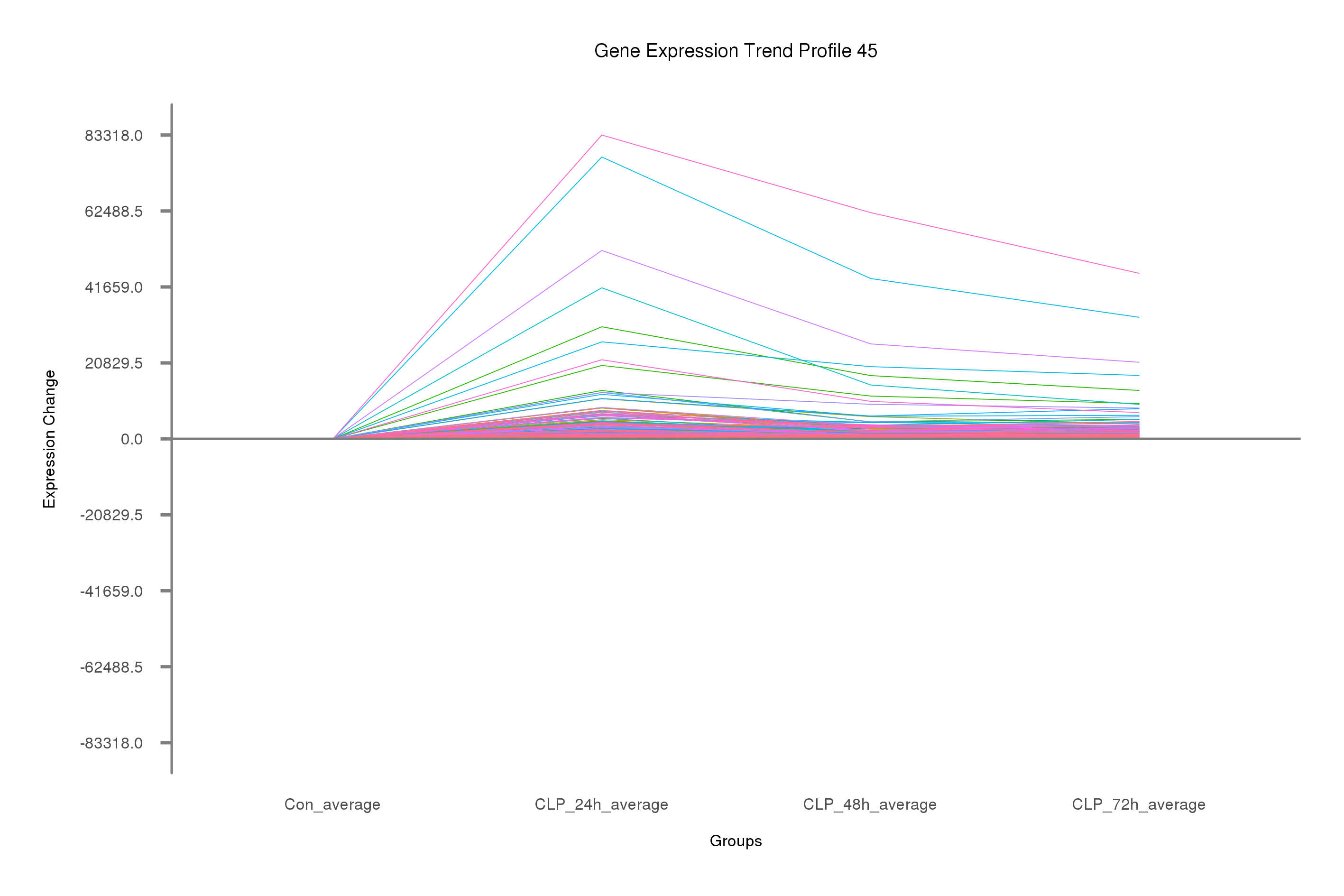

Supplement: Supplementary file 4 [file DataSheet1.zip › Data Sheet 1/Figure 4/45.png]

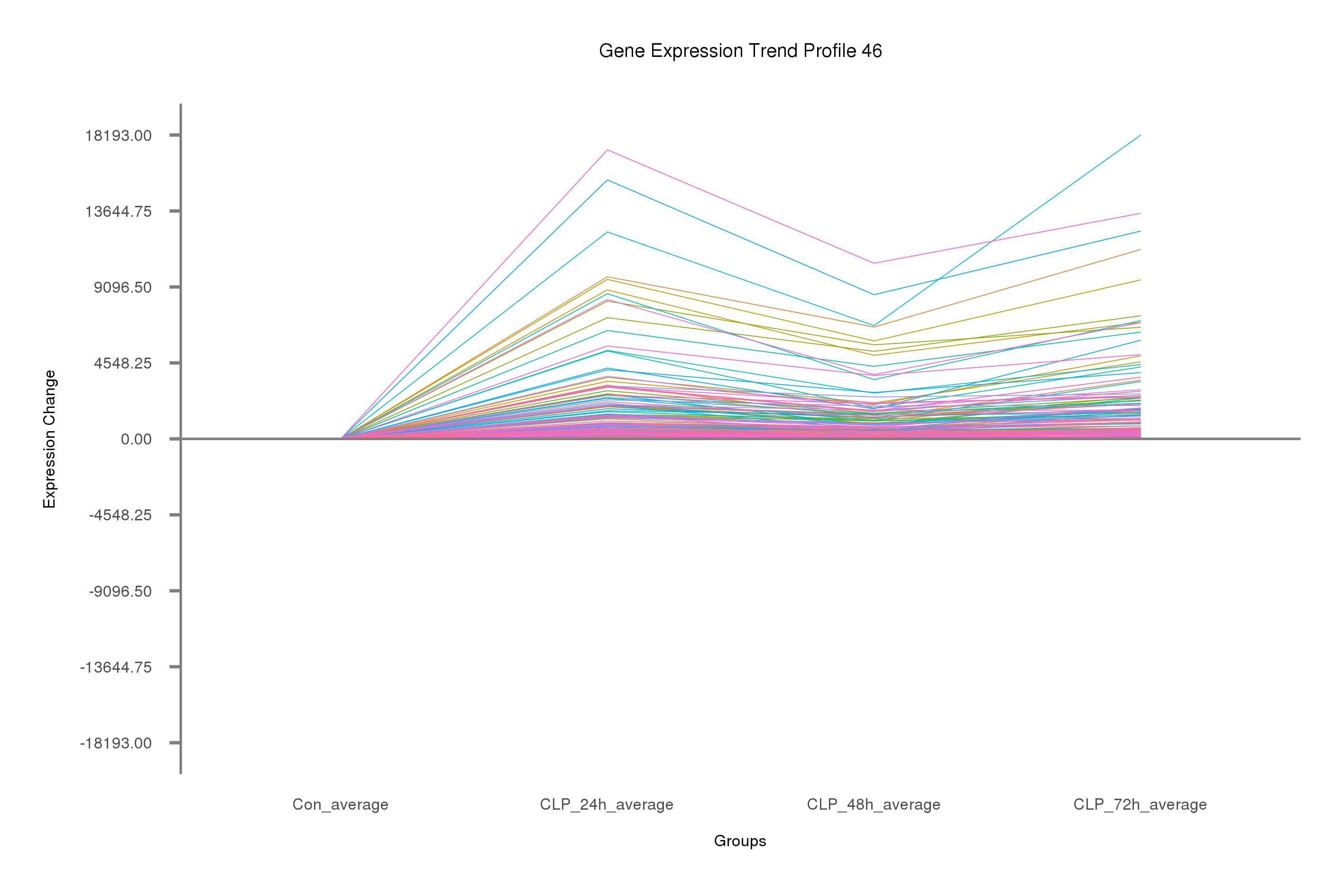

Supplement: Supplementary file 4 [file DataSheet1.zip › Data Sheet 1/Figure 4/46.png]

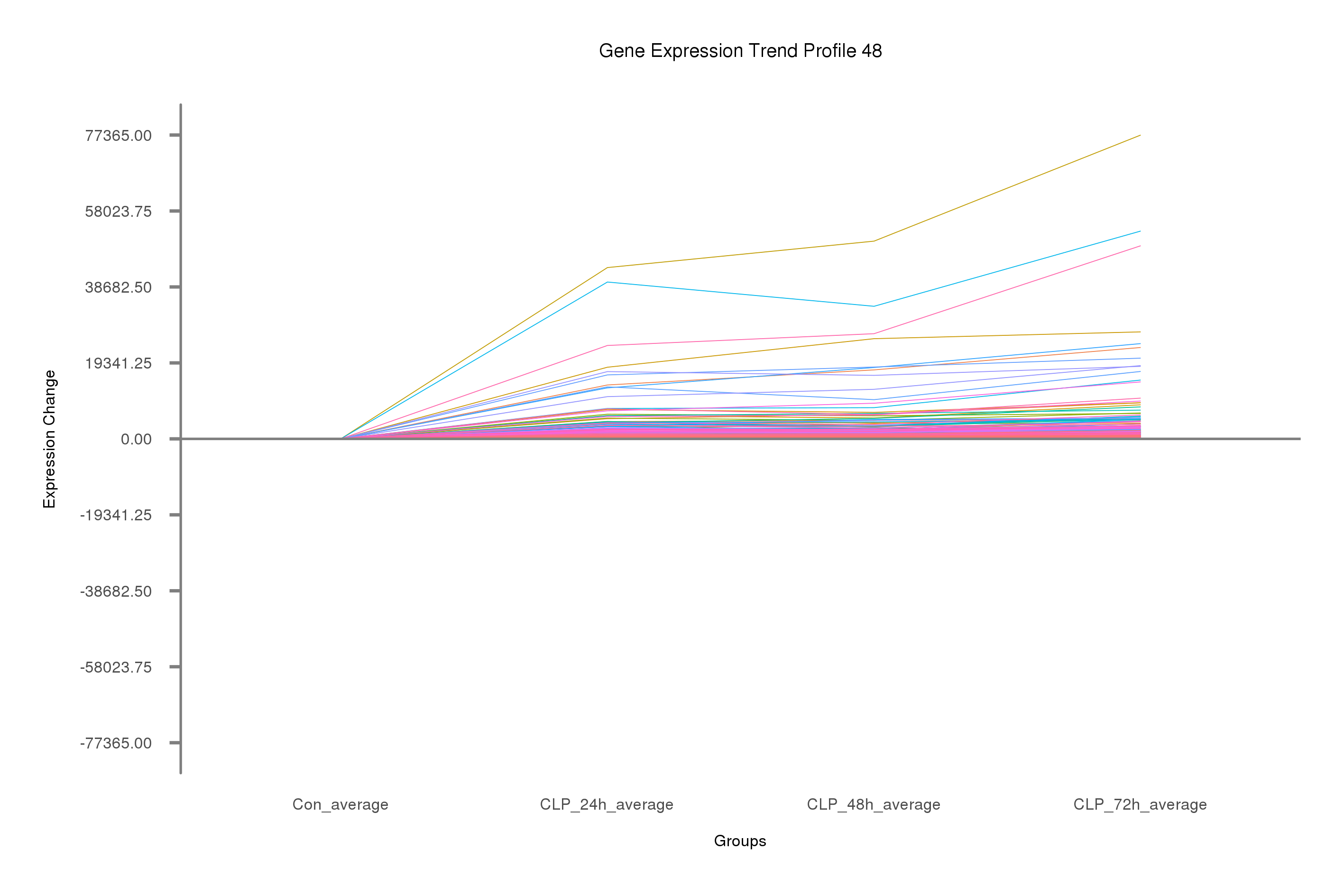

Supplement: Supplementary file 4 [file DataSheet1.zip › Data Sheet 1/Figure 4/48.png]

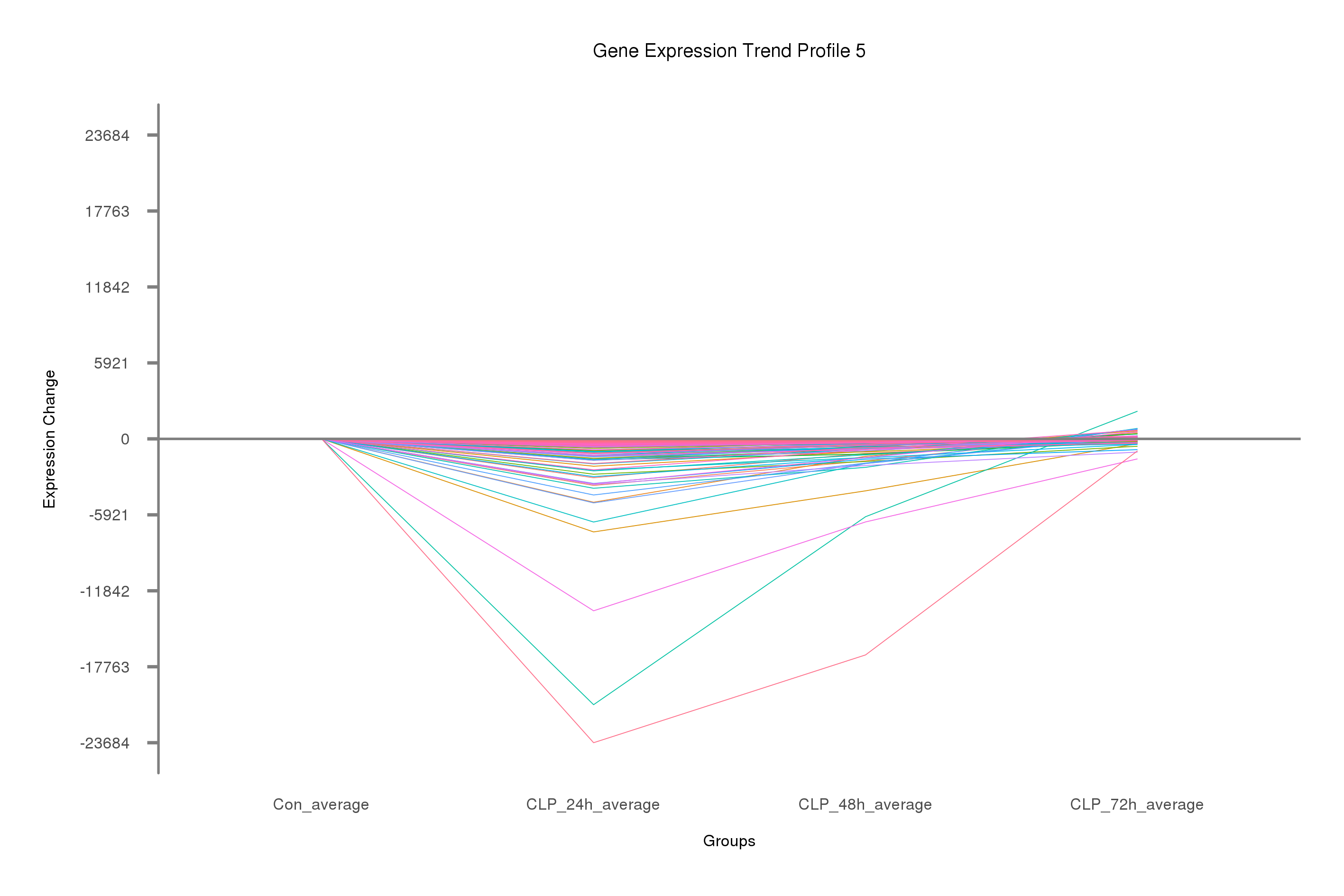

Supplement: Supplementary file 4 [file DataSheet1.zip › Data Sheet 1/Figure 4/5.png]

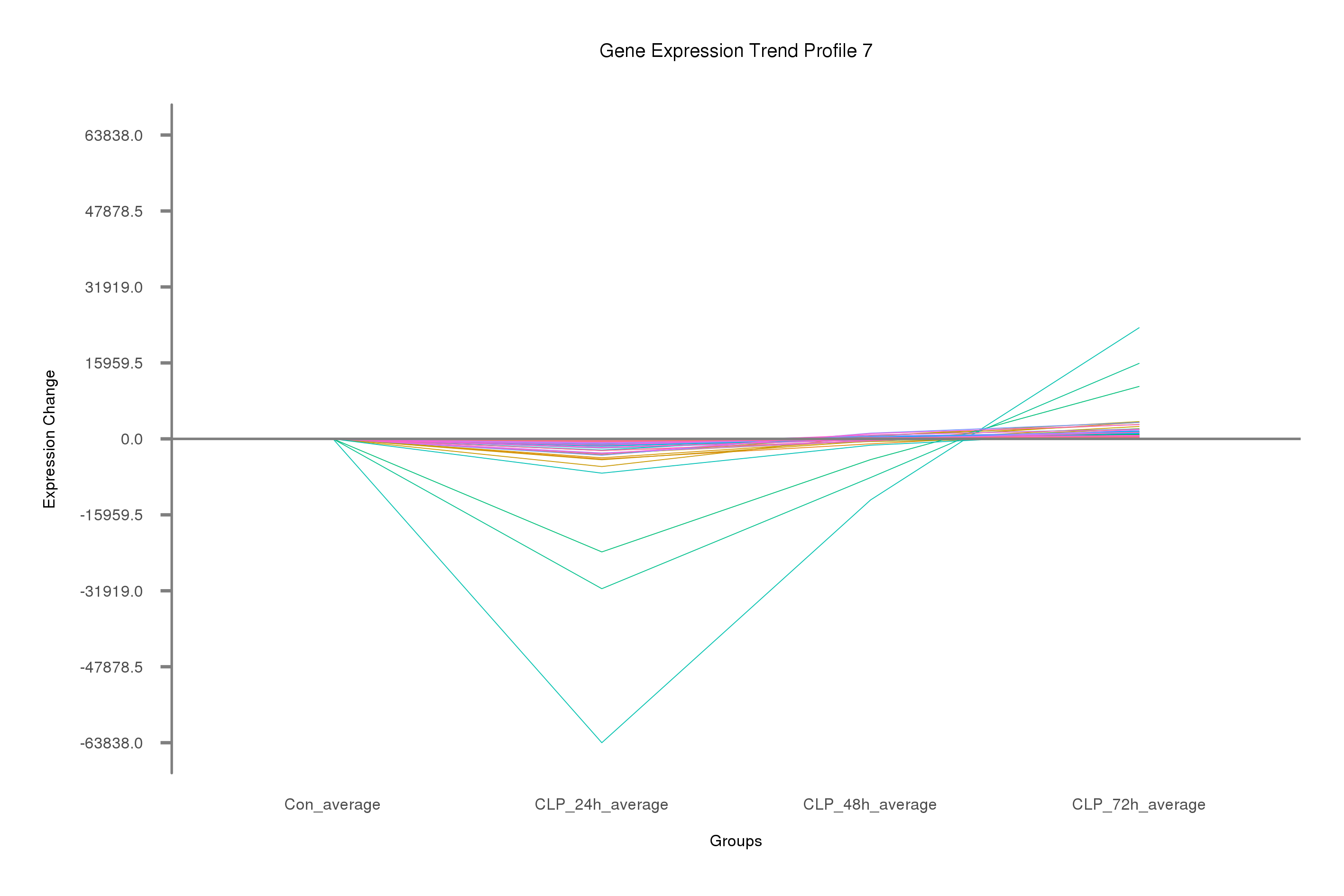

Supplement: Supplementary file 4 [file DataSheet1.zip › Data Sheet 1/Figure 4/7.png]

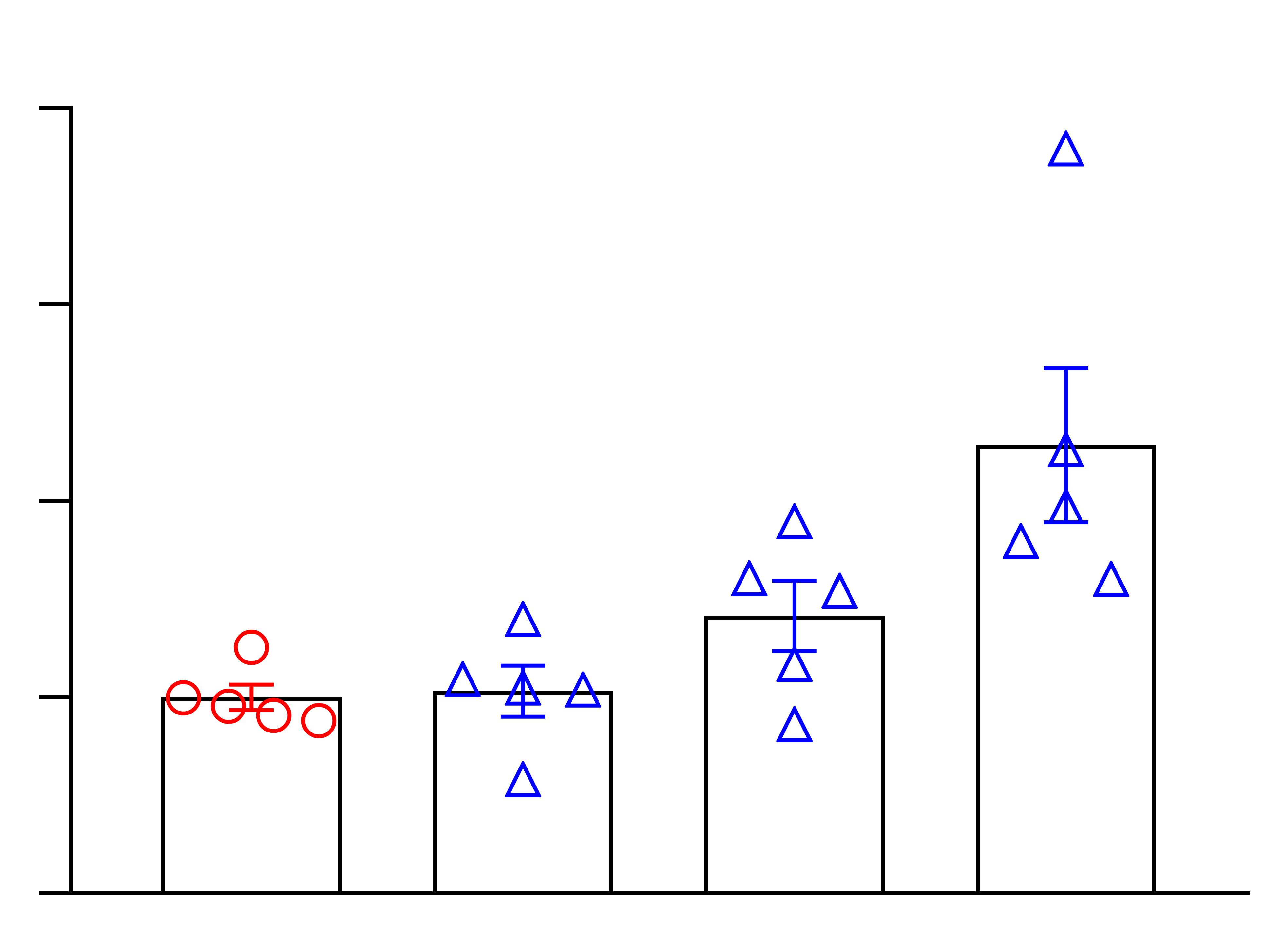

Supplement: Supplementary file 4 [file DataSheet1.zip › Data Sheet 1/Figure 6/Figure 6B/Pik3r1 time point RNASeq.jpg]

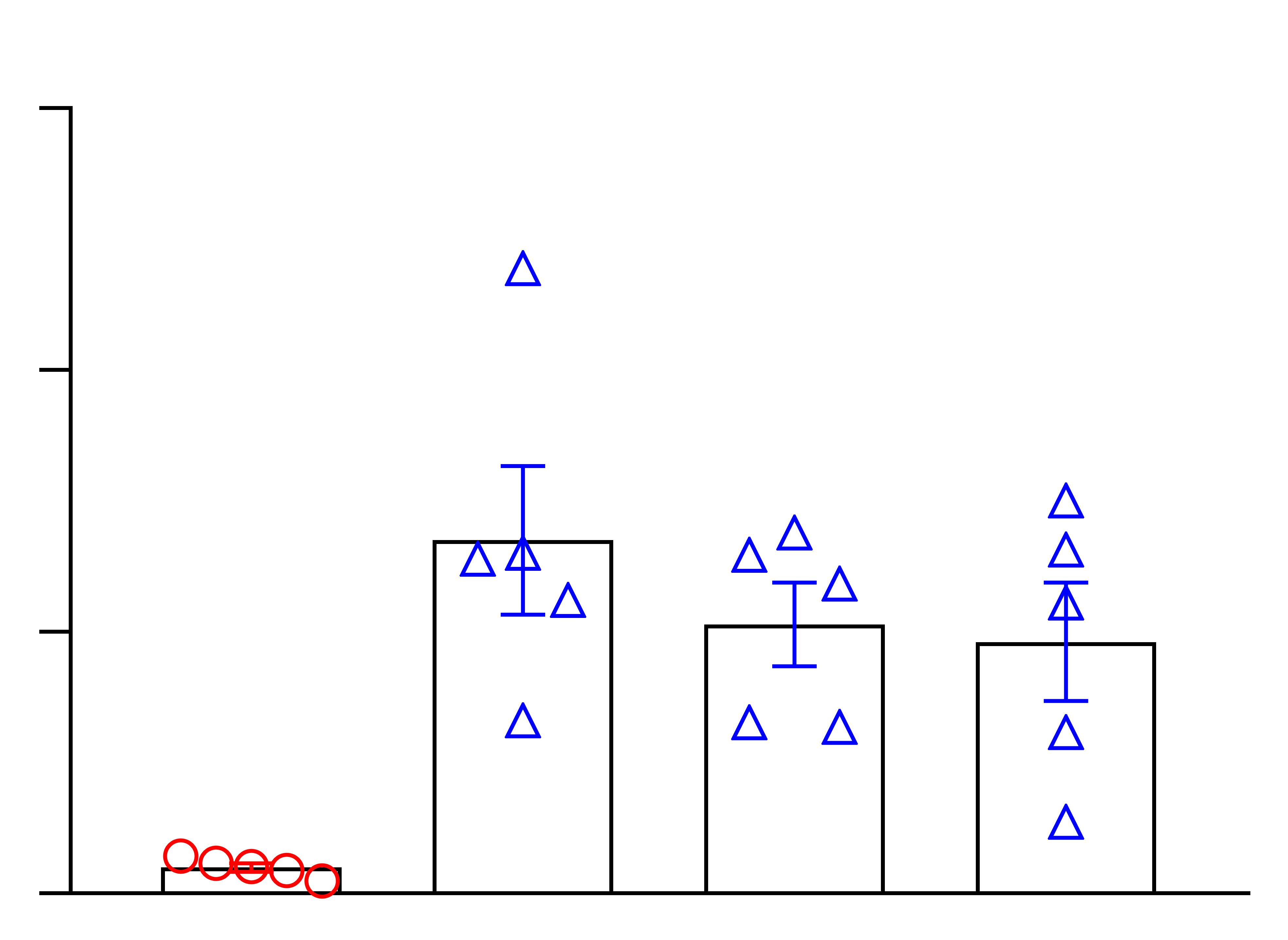

Supplement: Supplementary file 4 [file DataSheet1.zip › Data Sheet 1/Figure 6/Figure 6B/Pik3r5 time point RNASeq.jpg]

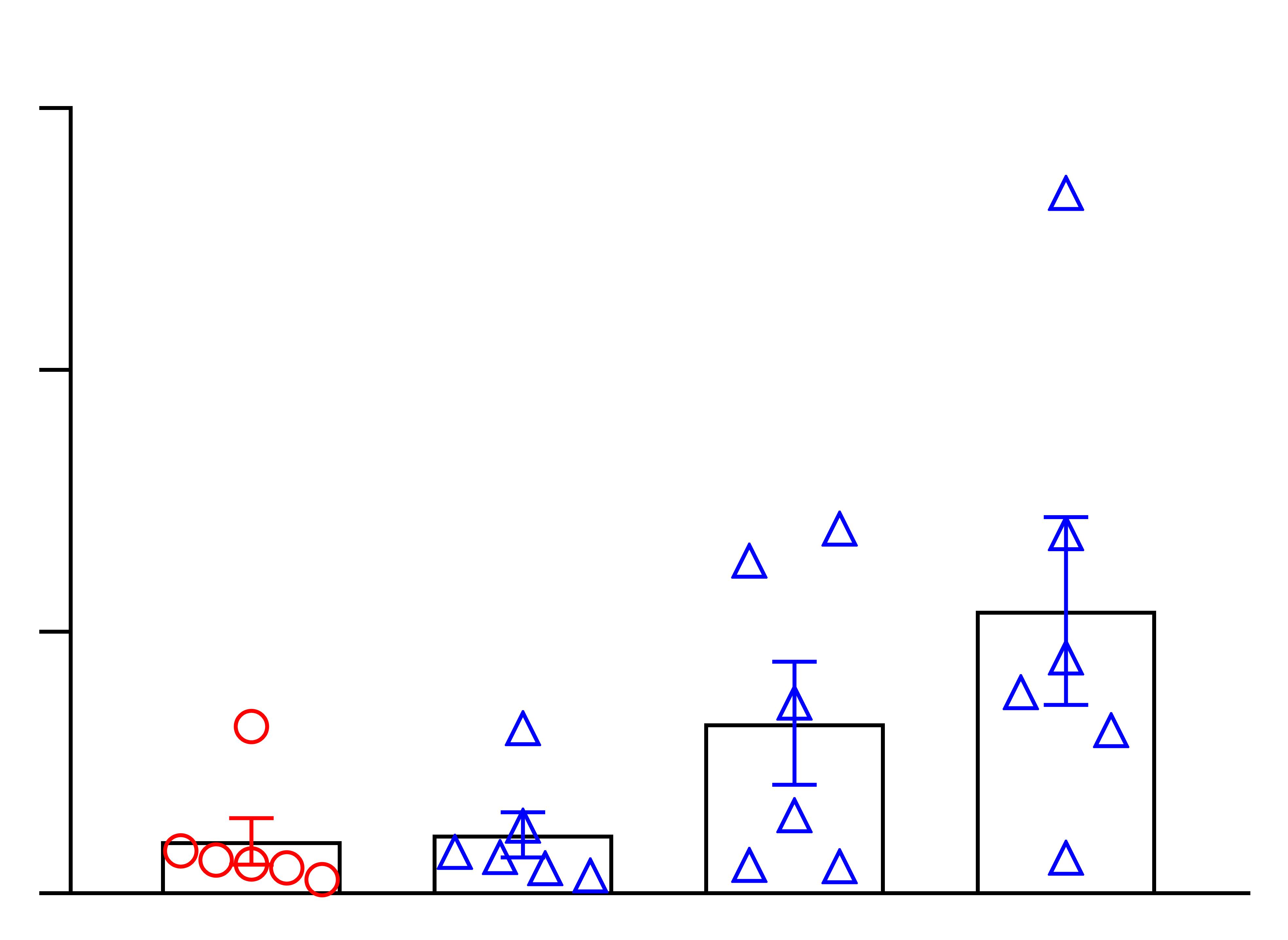

Supplement: Supplementary file 4 [file DataSheet1.zip › Data Sheet 1/Figure 6/Figure 6C/Pik3r1 time point qPCR.jpg]

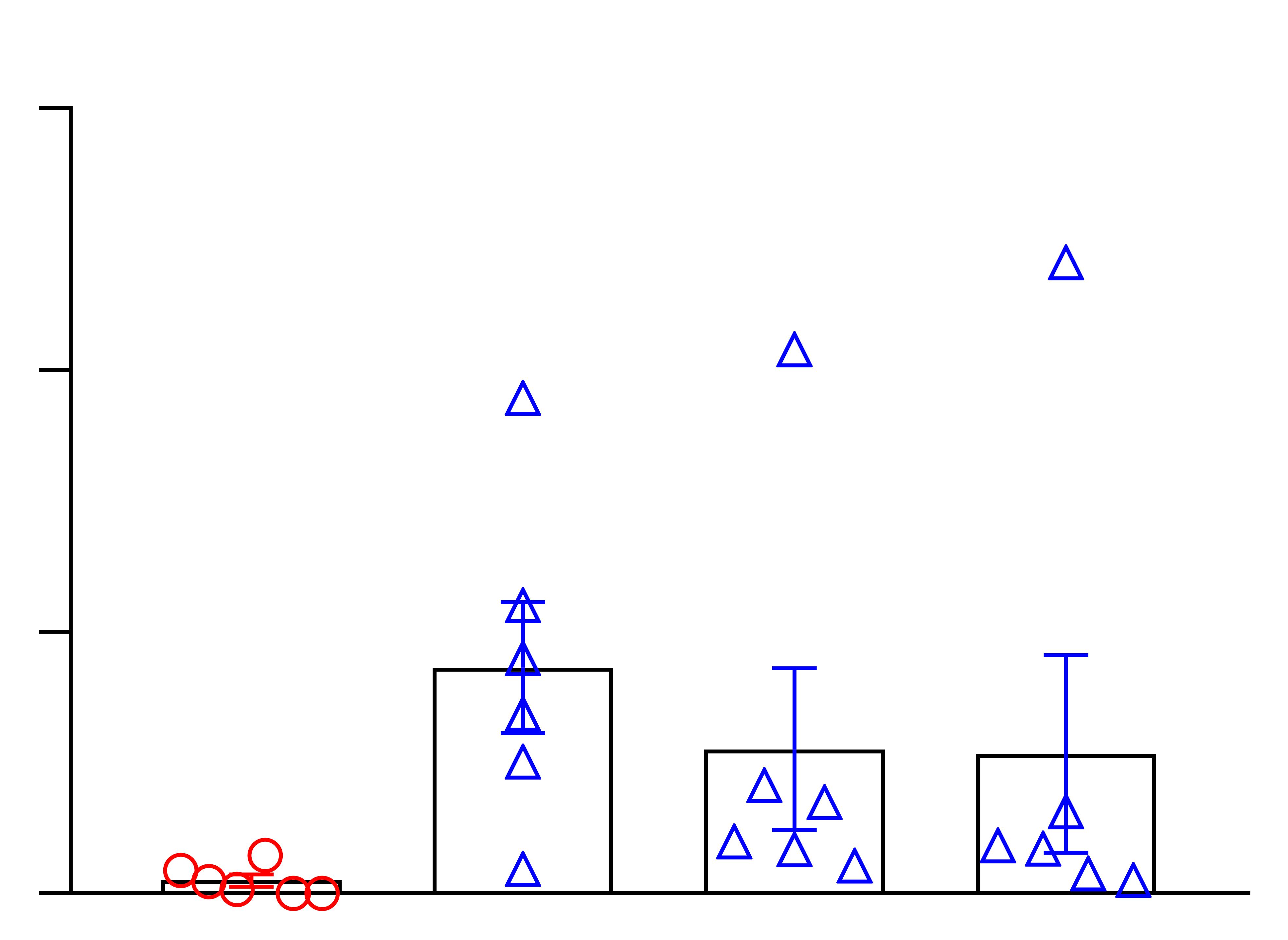

Supplement: Supplementary file 4 [file DataSheet1.zip › Data Sheet 1/Figure 6/Figure 6C/Pik3r5 time point qPCR.jpg]

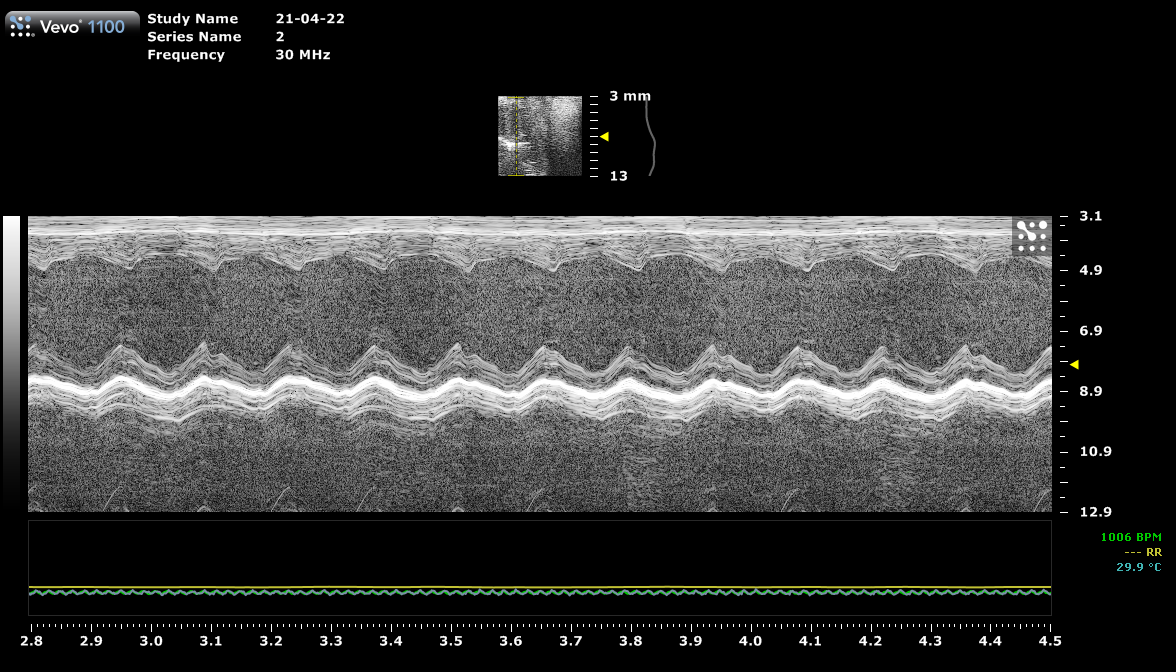

Supplement: Supplementary file 6 [file DataSheet2.zip › Data Sheet 2/Figure 7/Figure 7C/CLP original.tif]

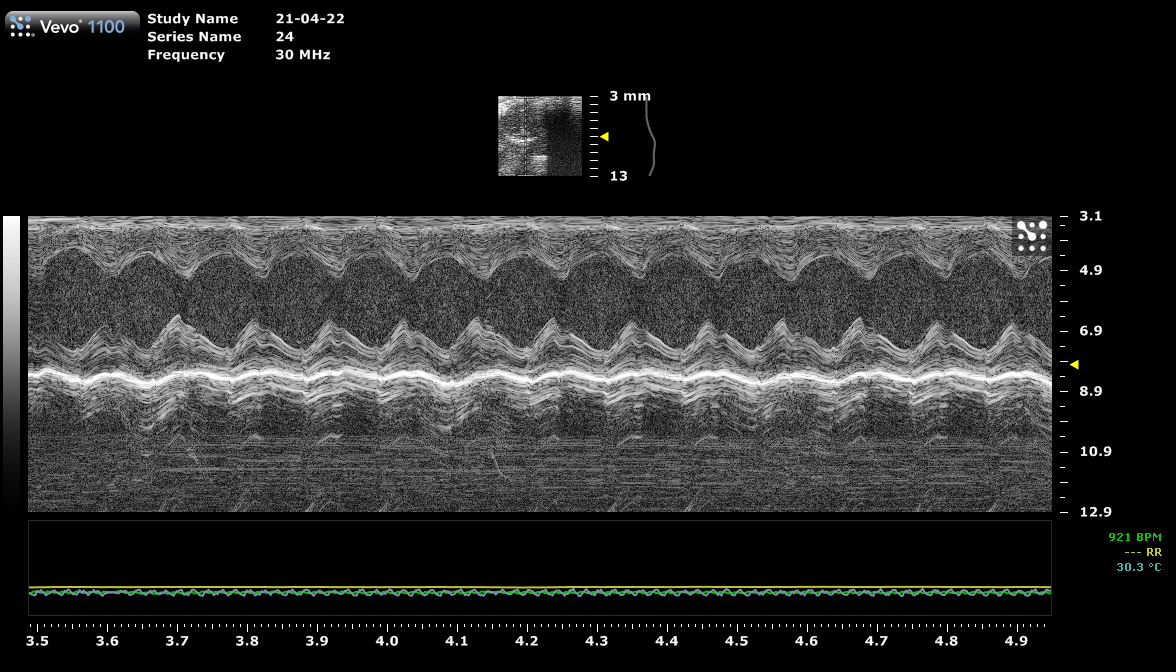

Supplement: Supplementary file 6 [file DataSheet2.zip › Data Sheet 2/Figure 7/Figure 7C/CLP+CZC original.tif]

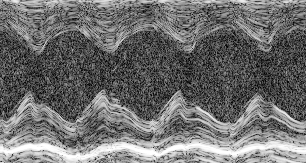

Supplement: Supplementary file 6 [file DataSheet2.zip › Data Sheet 2/Figure 7/Figure 7C/CLP+CZC.jpg]

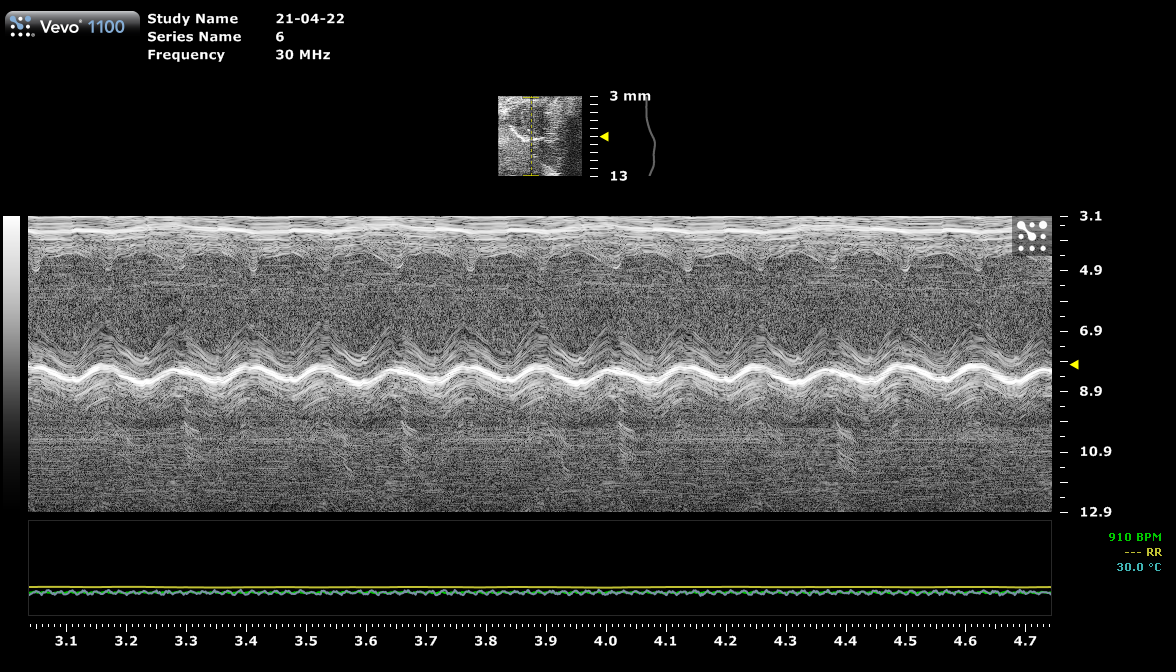

Supplement: Supplementary file 6 [file DataSheet2.zip › Data Sheet 2/Figure 7/Figure 7C/CLP+LY original.tif]

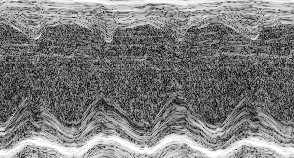

Supplement: Supplementary file 6 [file DataSheet2.zip › Data Sheet 2/Figure 7/Figure 7C/CLP+LY.jpg]

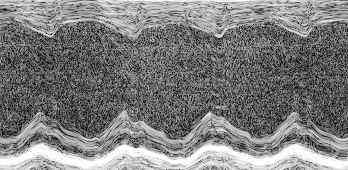

Supplement: Supplementary file 6 [file DataSheet2.zip › Data Sheet 2/Figure 7/Figure 7C/CLP.jpg]

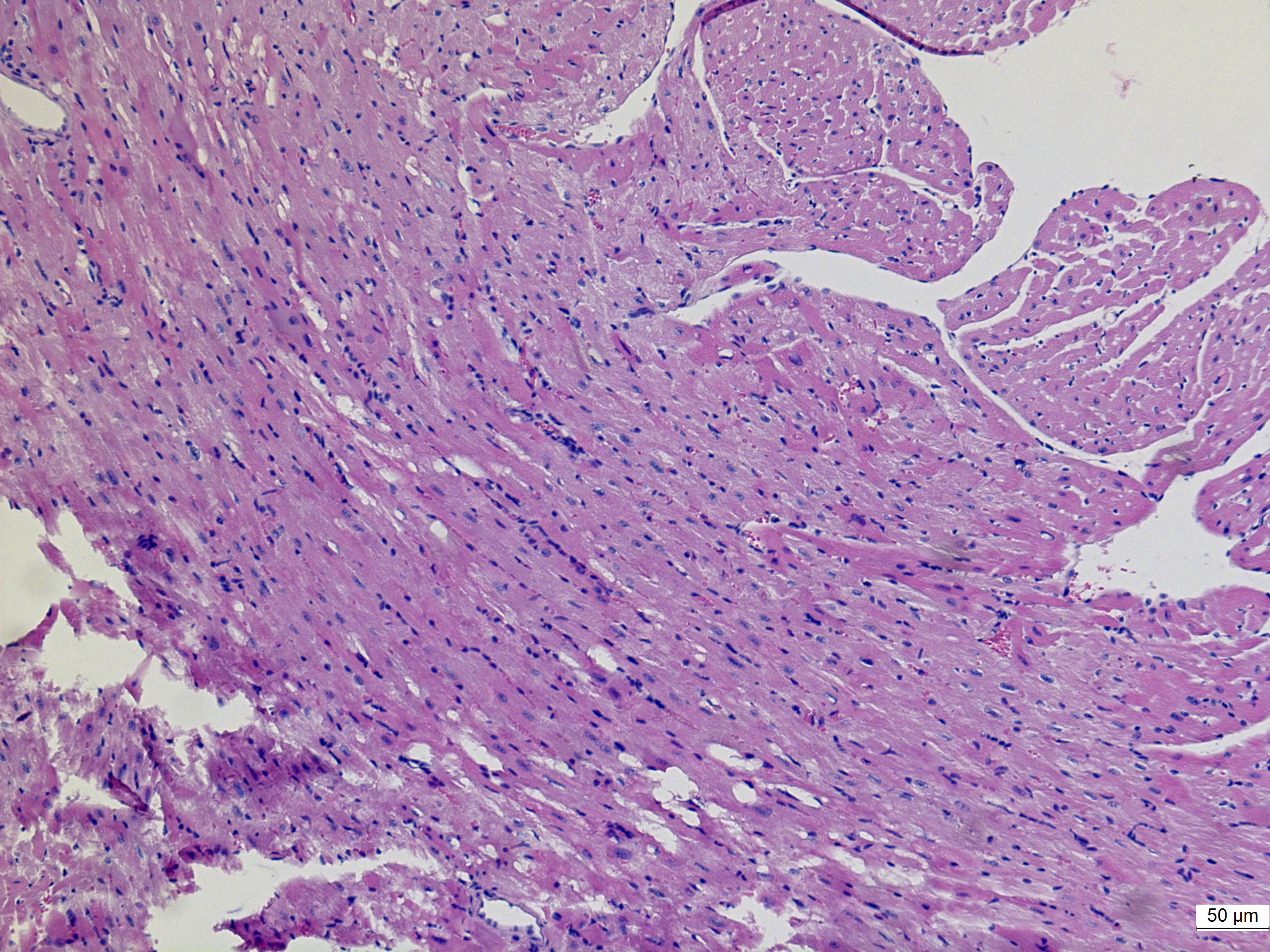

Supplement: Supplementary file 6 [file DataSheet2.zip › Data Sheet 2/Figure 7/Figure 7D/HE/CLP original.jpg]

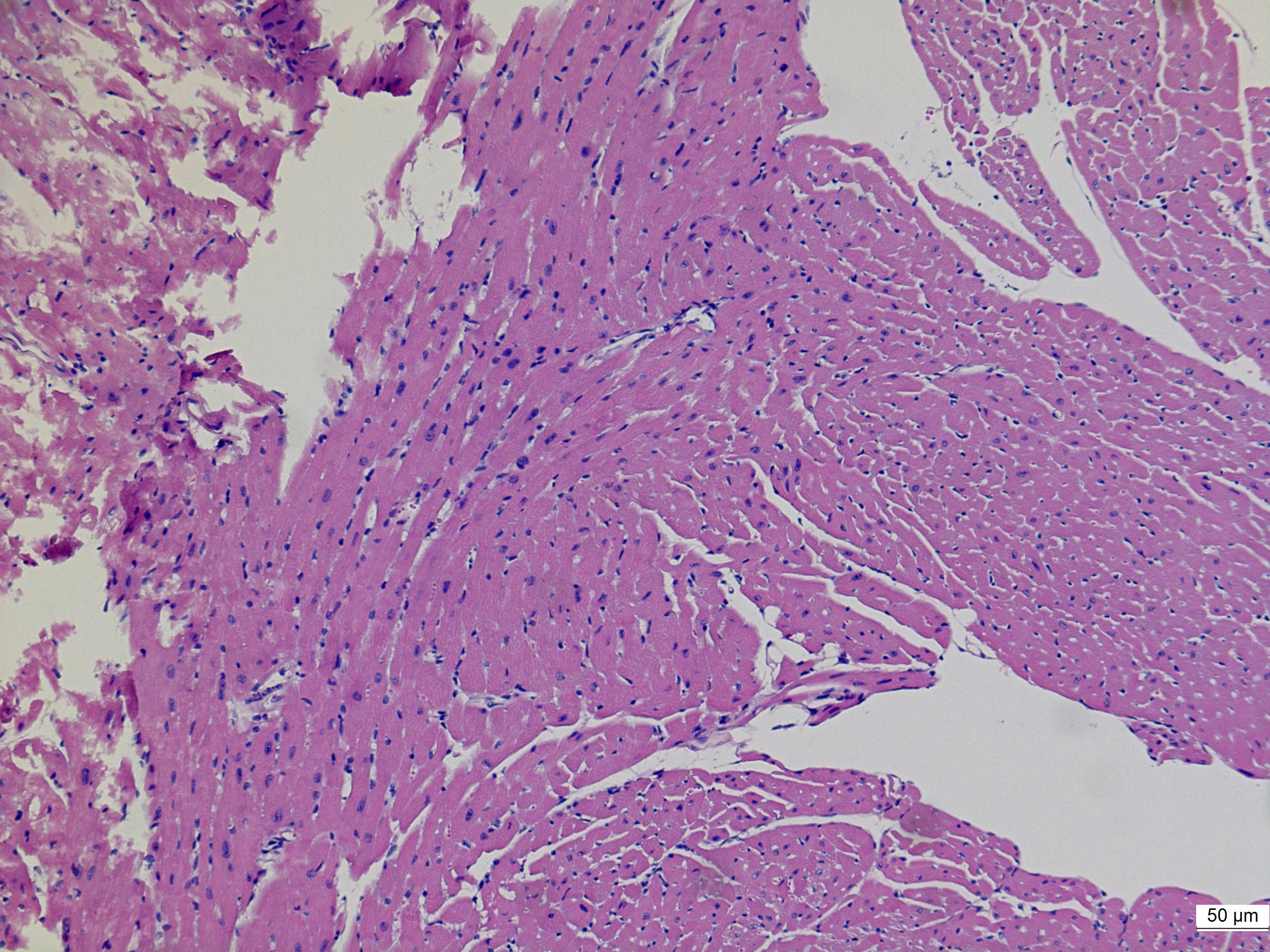

Supplement: Supplementary file 6 [file DataSheet2.zip › Data Sheet 2/Figure 7/Figure 7D/HE/CLP+CZC original.jpg]

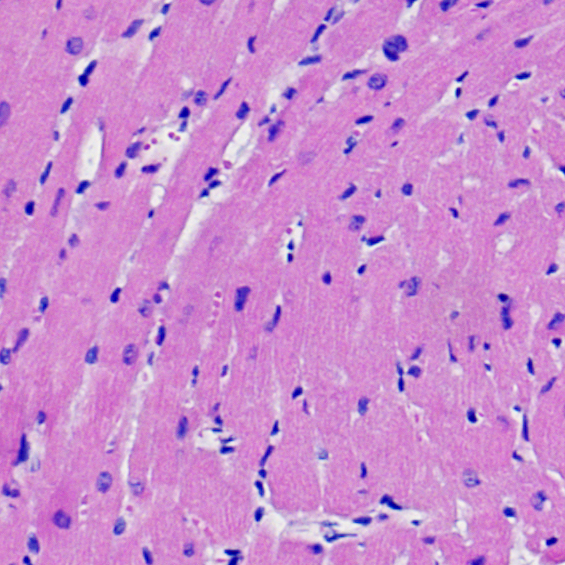

Supplement: Supplementary file 6 [file DataSheet2.zip › Data Sheet 2/Figure 7/Figure 7D/HE/CLP+CZC.jpg]

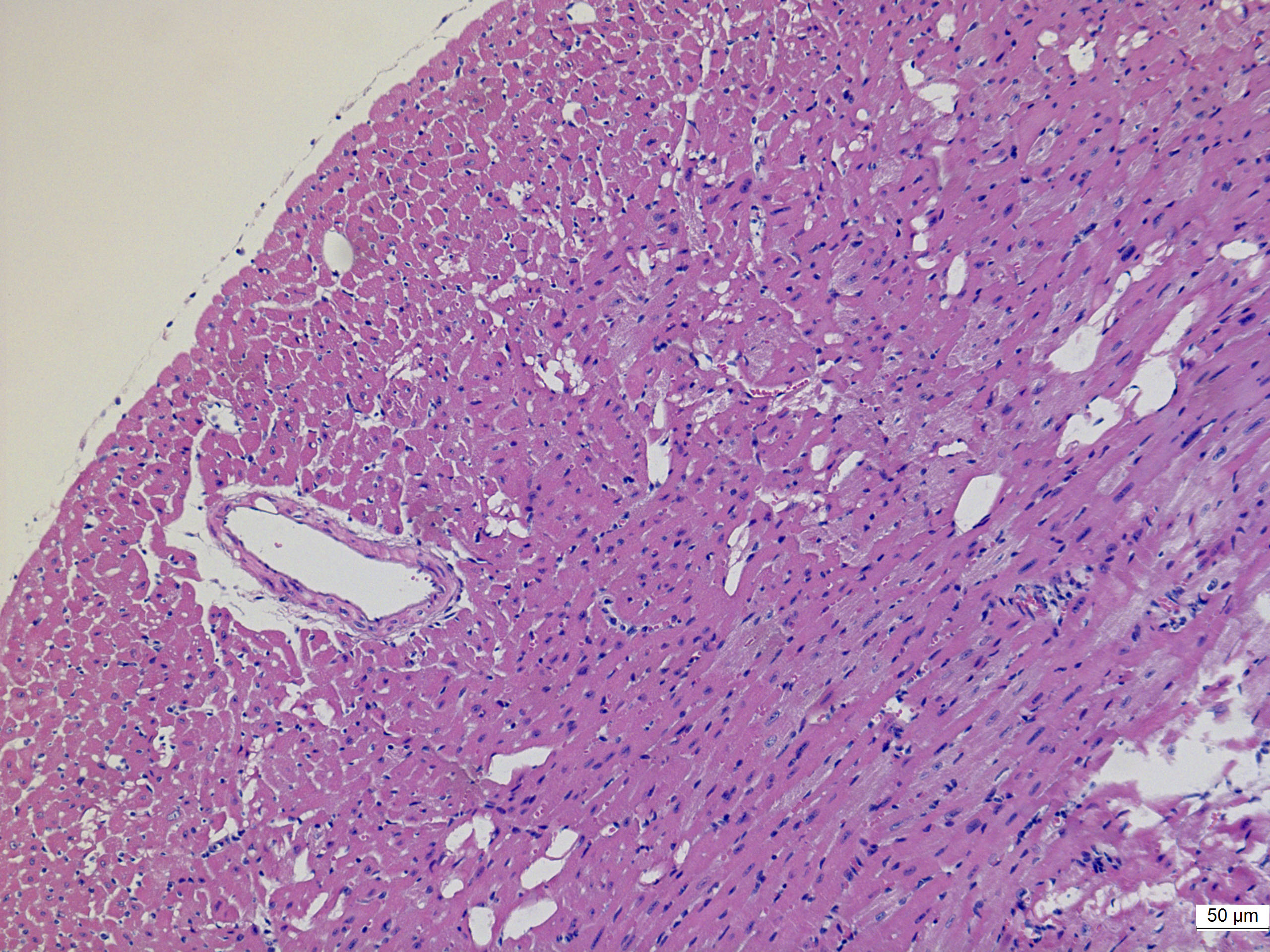

Supplement: Supplementary file 6 [file DataSheet2.zip › Data Sheet 2/Figure 7/Figure 7D/HE/CLP+LY original.jpg]

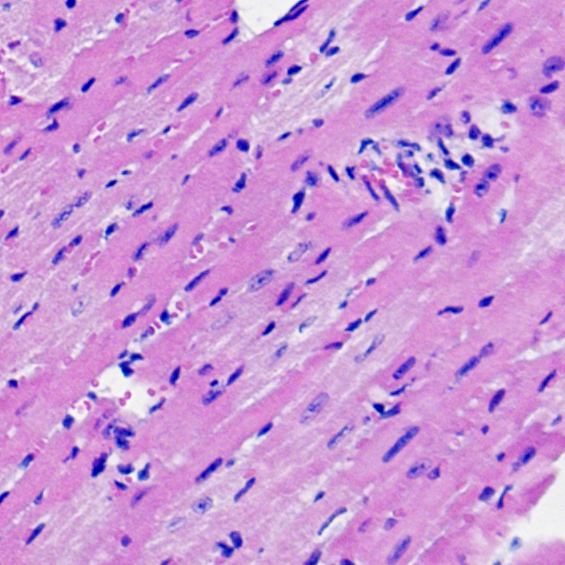

Supplement: Supplementary file 6 [file DataSheet2.zip › Data Sheet 2/Figure 7/Figure 7D/HE/CLP+LY.jpg]

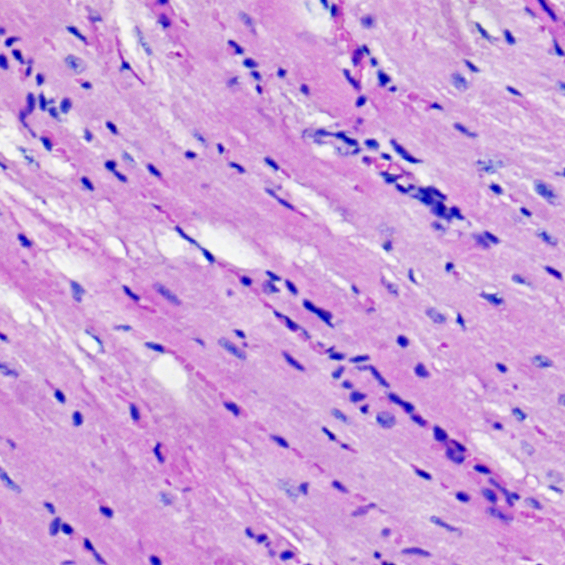

Supplement: Supplementary file 6 [file DataSheet2.zip › Data Sheet 2/Figure 7/Figure 7D/HE/CLP.jpg]

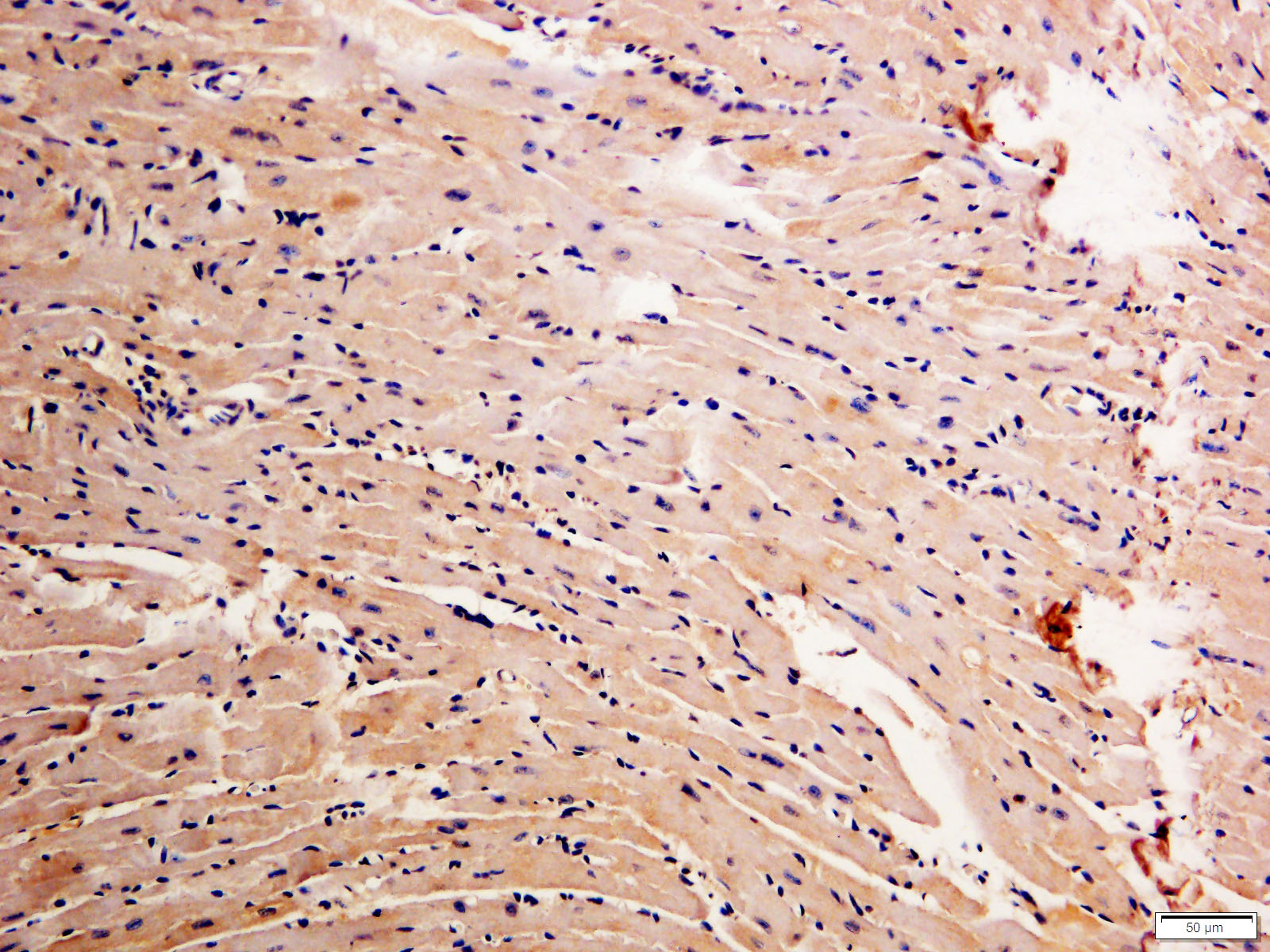

Supplement: Supplementary file 6 [file DataSheet2.zip › Data Sheet 2/Figure 7/Figure 7D/Mac-2/CLP original.jpg]

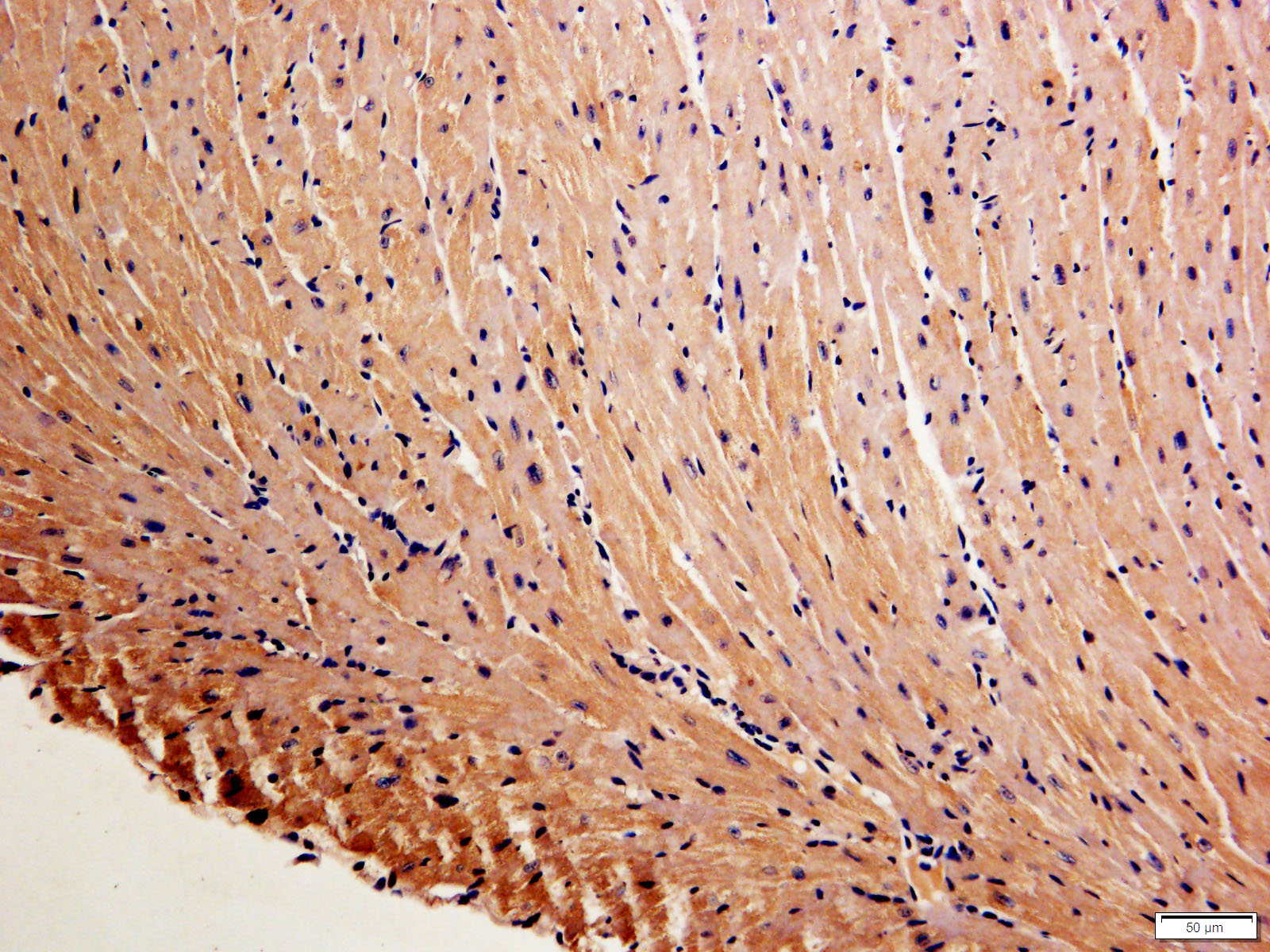

Supplement: Supplementary file 6 [file DataSheet2.zip › Data Sheet 2/Figure 7/Figure 7D/Mac-2/CLP+CZC original.jpg]

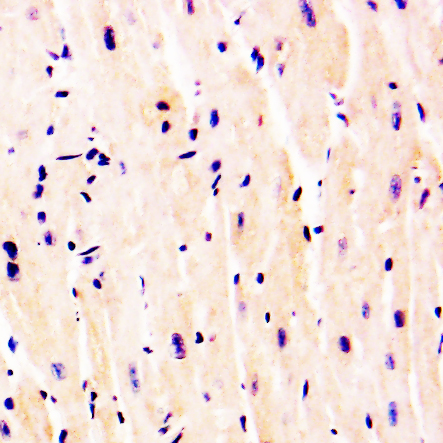

Supplement: Supplementary file 6 [file DataSheet2.zip › Data Sheet 2/Figure 7/Figure 7D/Mac-2/CLP+CZC.jpg]

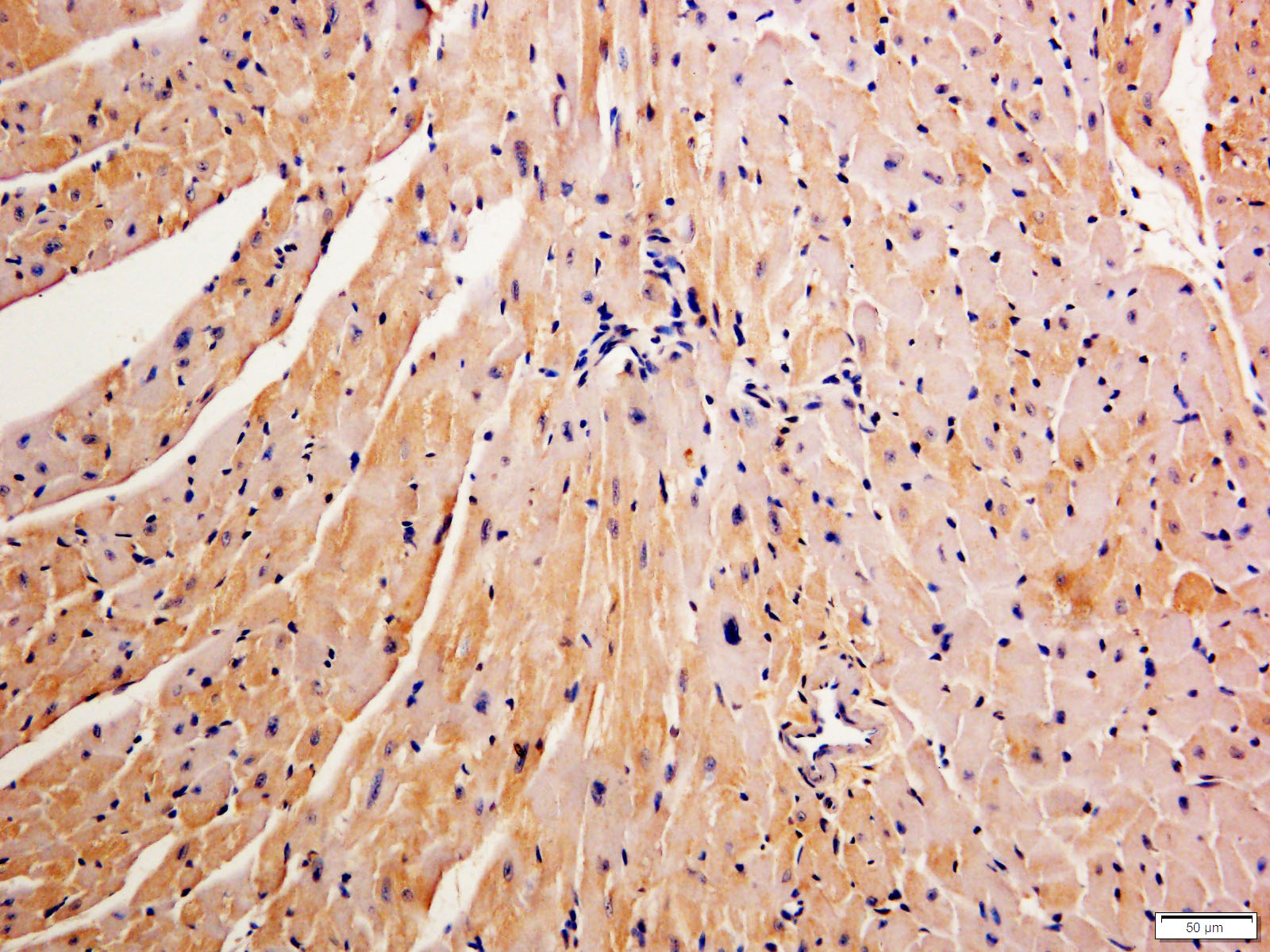

Supplement: Supplementary file 6 [file DataSheet2.zip › Data Sheet 2/Figure 7/Figure 7D/Mac-2/CLP+LY original.jpg]

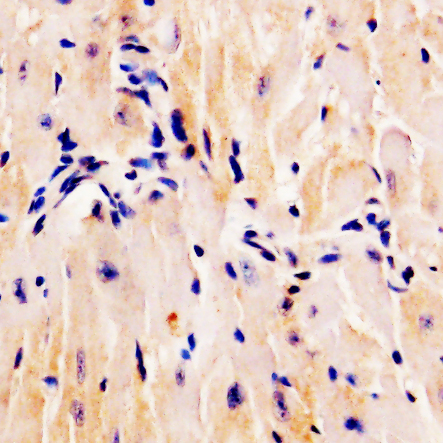

Supplement: Supplementary file 6 [file DataSheet2.zip › Data Sheet 2/Figure 7/Figure 7D/Mac-2/CLP+LY.jpg]

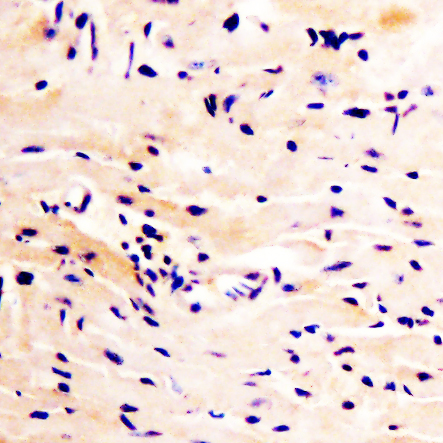

Supplement: Supplementary file 6 [file DataSheet2.zip › Data Sheet 2/Figure 7/Figure 7D/Mac-2/CLP.jpg]

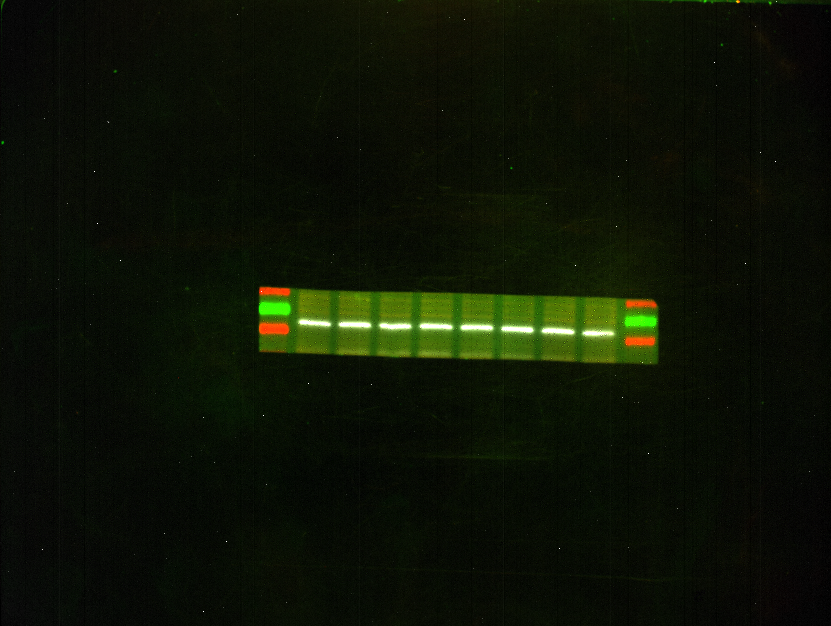

Supplement: Supplementary file 6 [file DataSheet2.zip › Data Sheet 2/Figure 7/Figure 7E/AKT/AKT Marker.png]

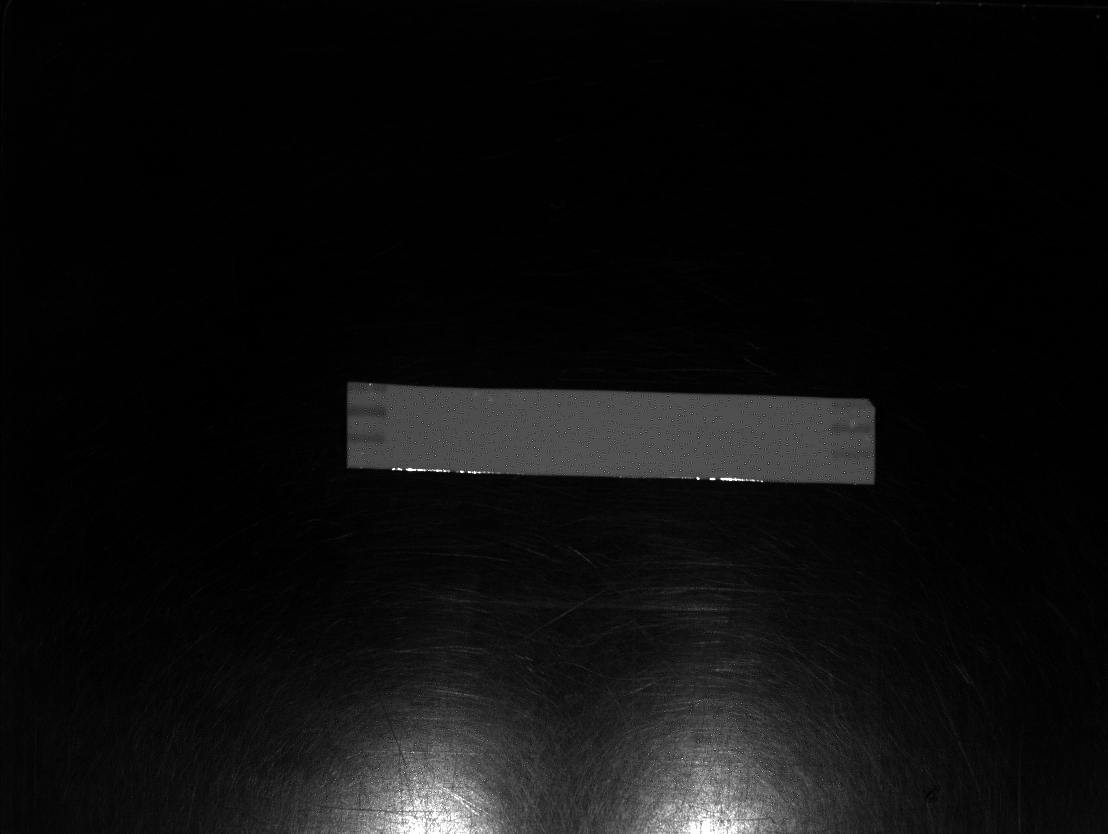

Supplement: Supplementary file 6 [file DataSheet2.zip › Data Sheet 2/Figure 7/Figure 7E/AKT/AKT Membrane.png]

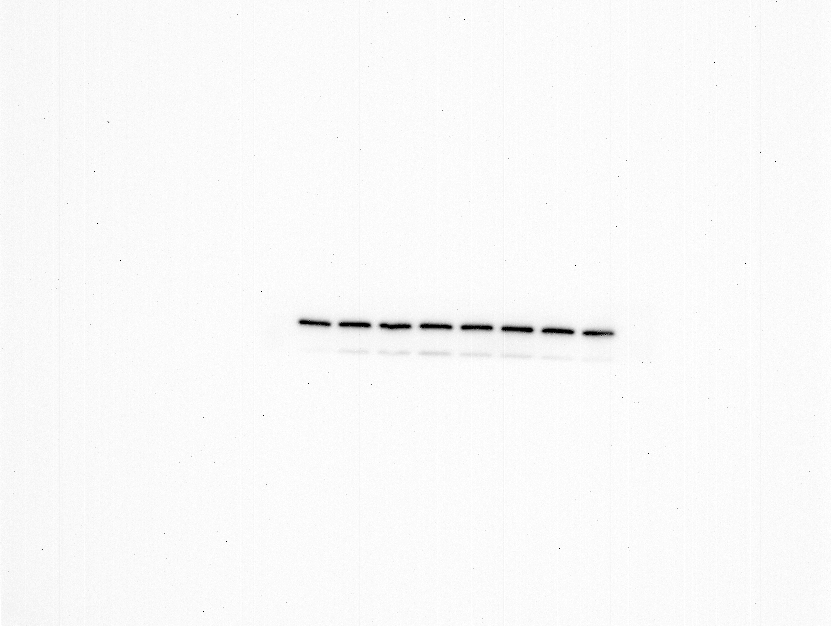

Supplement: Supplementary file 6 [file DataSheet2.zip › Data Sheet 2/Figure 7/Figure 7E/AKT/AKT.jpg]

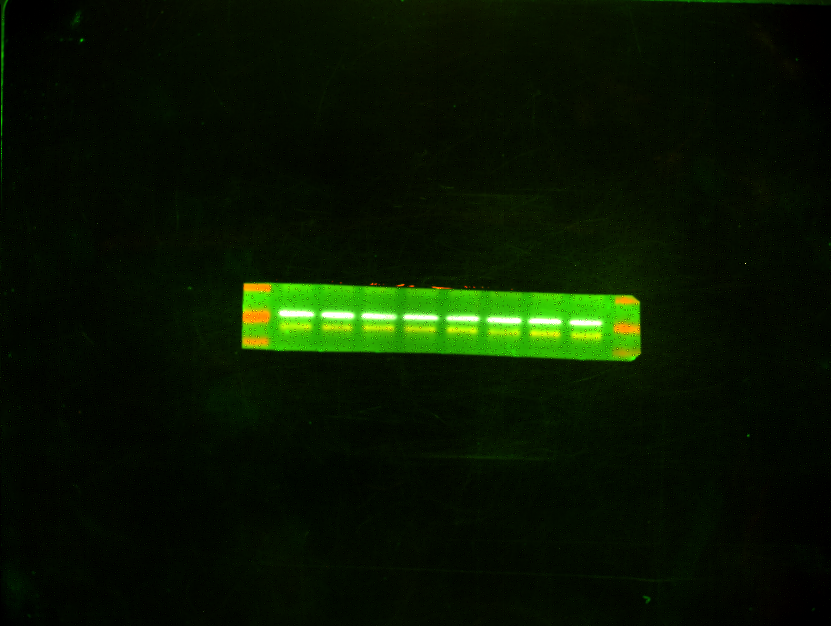

Supplement: Supplementary file 6 [file DataSheet2.zip › Data Sheet 2/Figure 7/Figure 7E/GAPDH/GAPDH Marker.png]

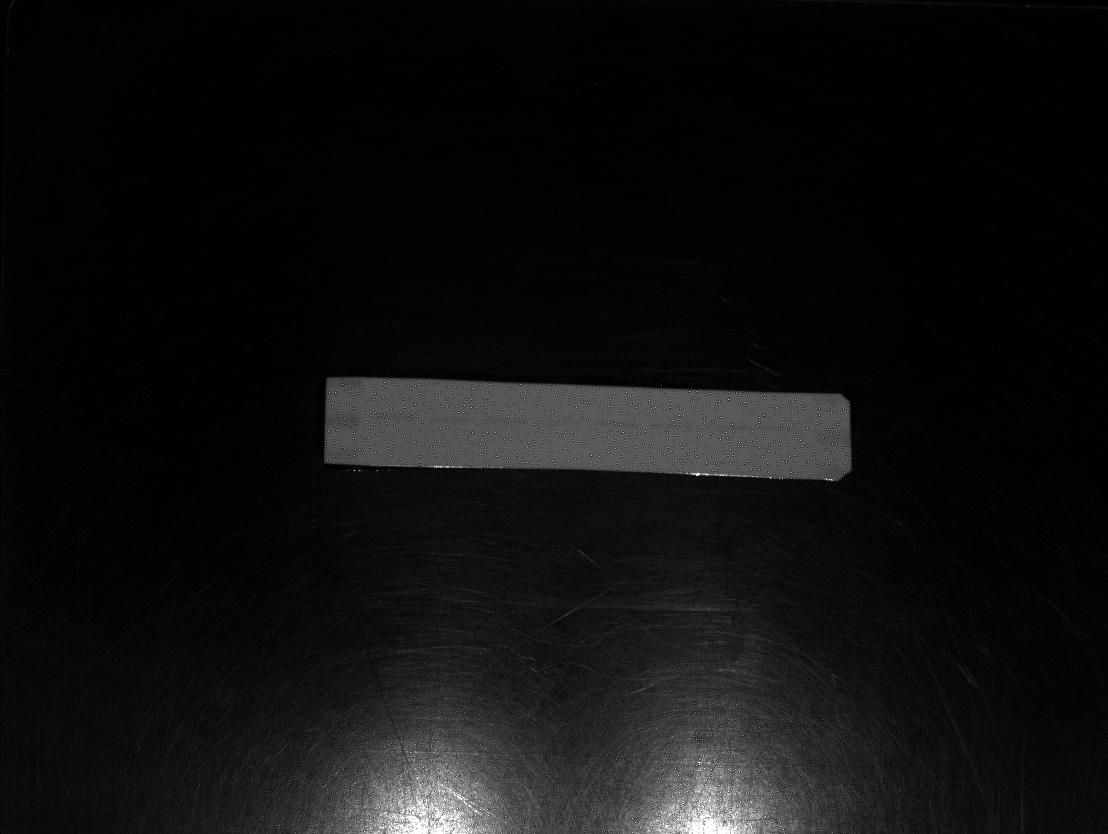

Supplement: Supplementary file 6 [file DataSheet2.zip › Data Sheet 2/Figure 7/Figure 7E/GAPDH/GAPDH Membrane.png]

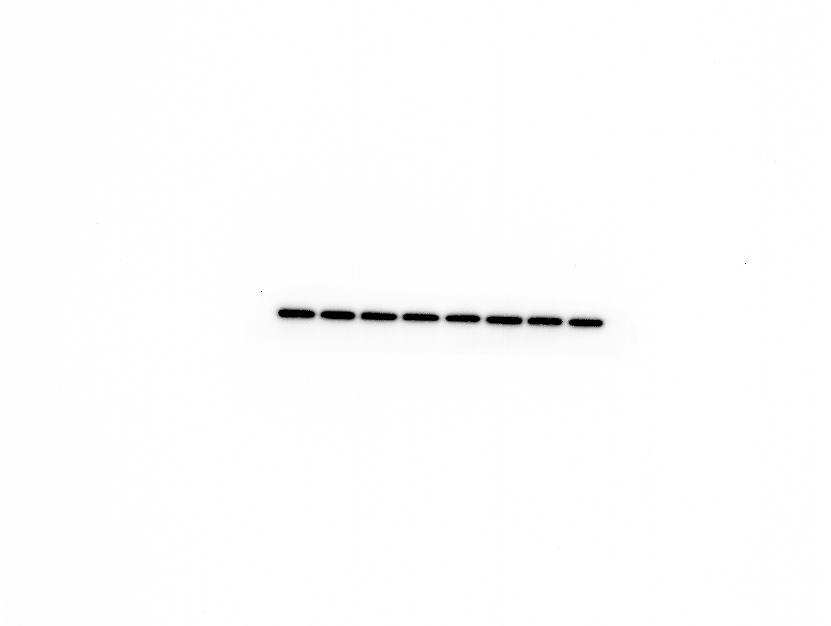

Supplement: Supplementary file 6 [file DataSheet2.zip › Data Sheet 2/Figure 7/Figure 7E/GAPDH/GAPDH.jpg]

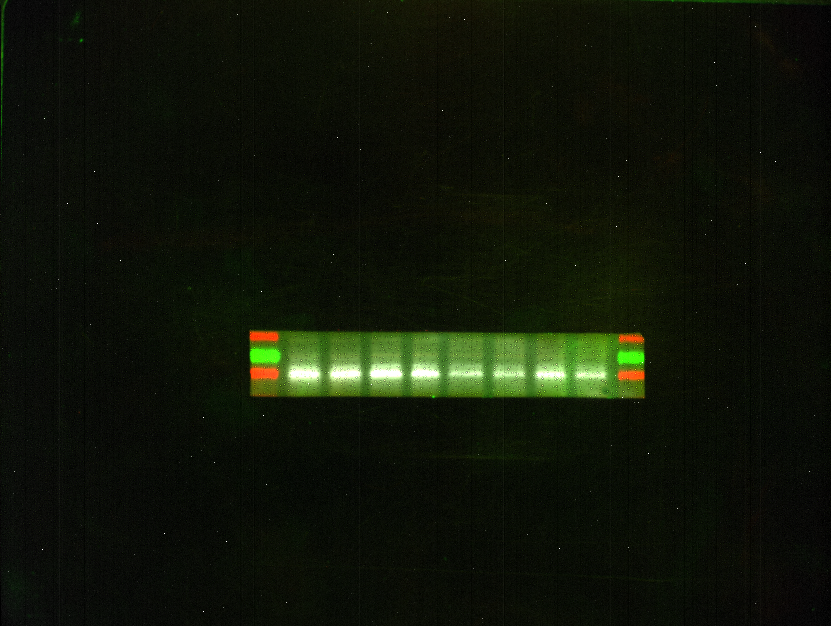

Supplement: Supplementary file 6 [file DataSheet2.zip › Data Sheet 2/Figure 7/Figure 7E/p-AKT/p-AKT Marker.png]

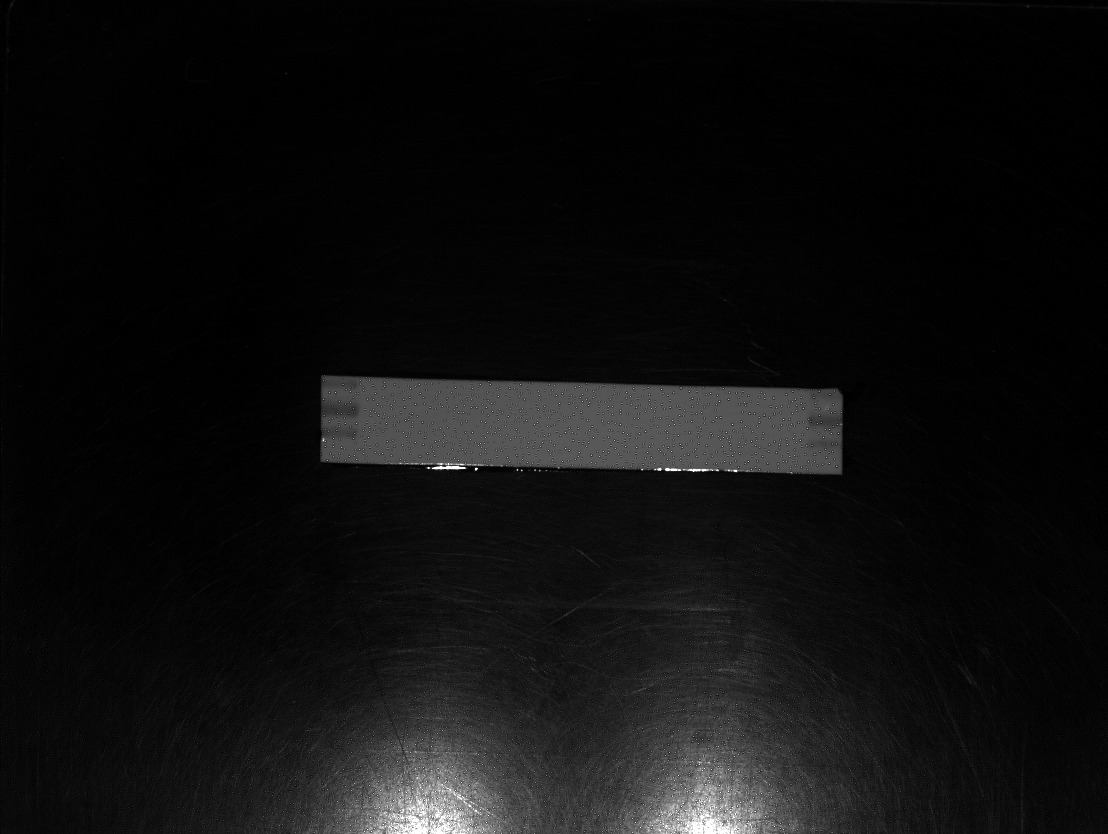

Supplement: Supplementary file 6 [file DataSheet2.zip › Data Sheet 2/Figure 7/Figure 7E/p-AKT/p-AKT Membrane.png]

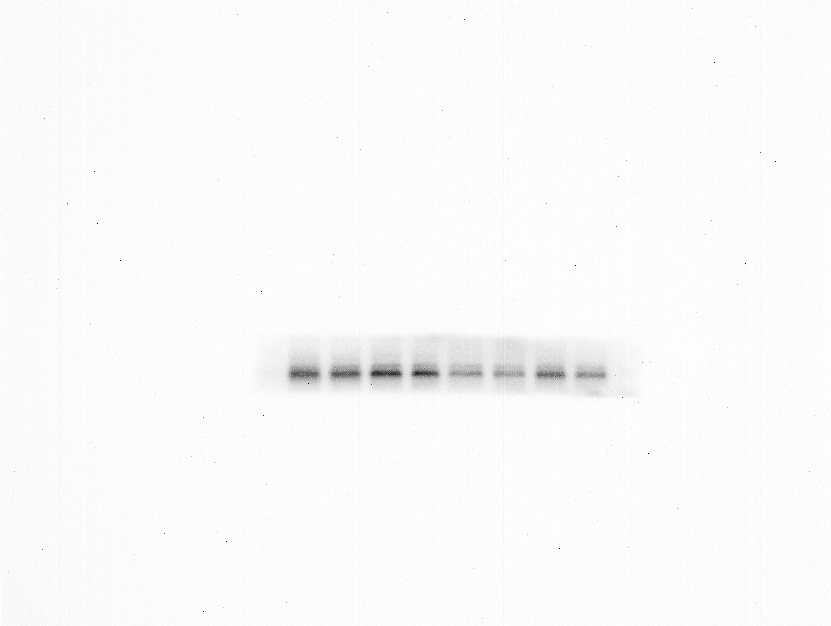

Supplement: Supplementary file 6 [file DataSheet2.zip › Data Sheet 2/Figure 7/Figure 7E/p-AKT/p-AKT.jpg]

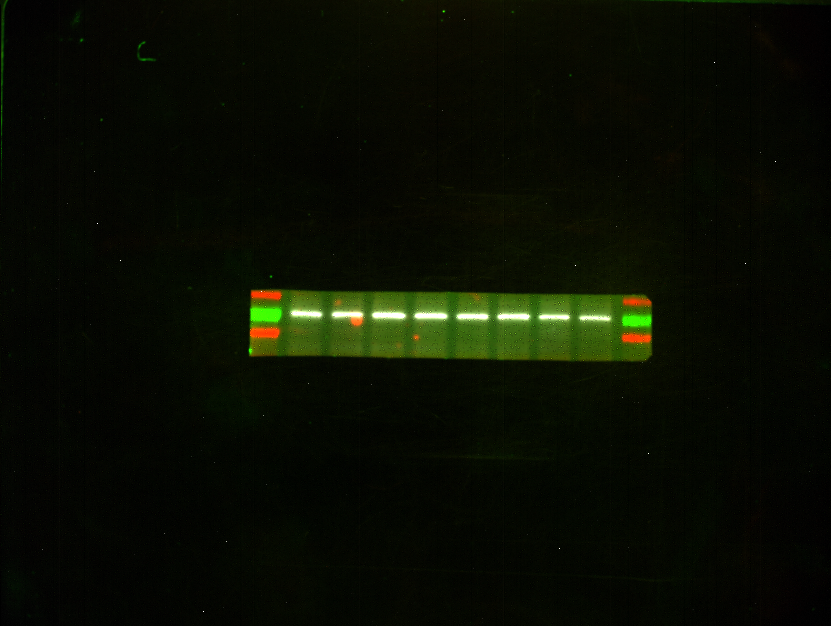

Supplement: Supplementary file 6 [file DataSheet2.zip › Data Sheet 2/Figure 7/Figure 7E/p-p65/p-p65 Marker.png]

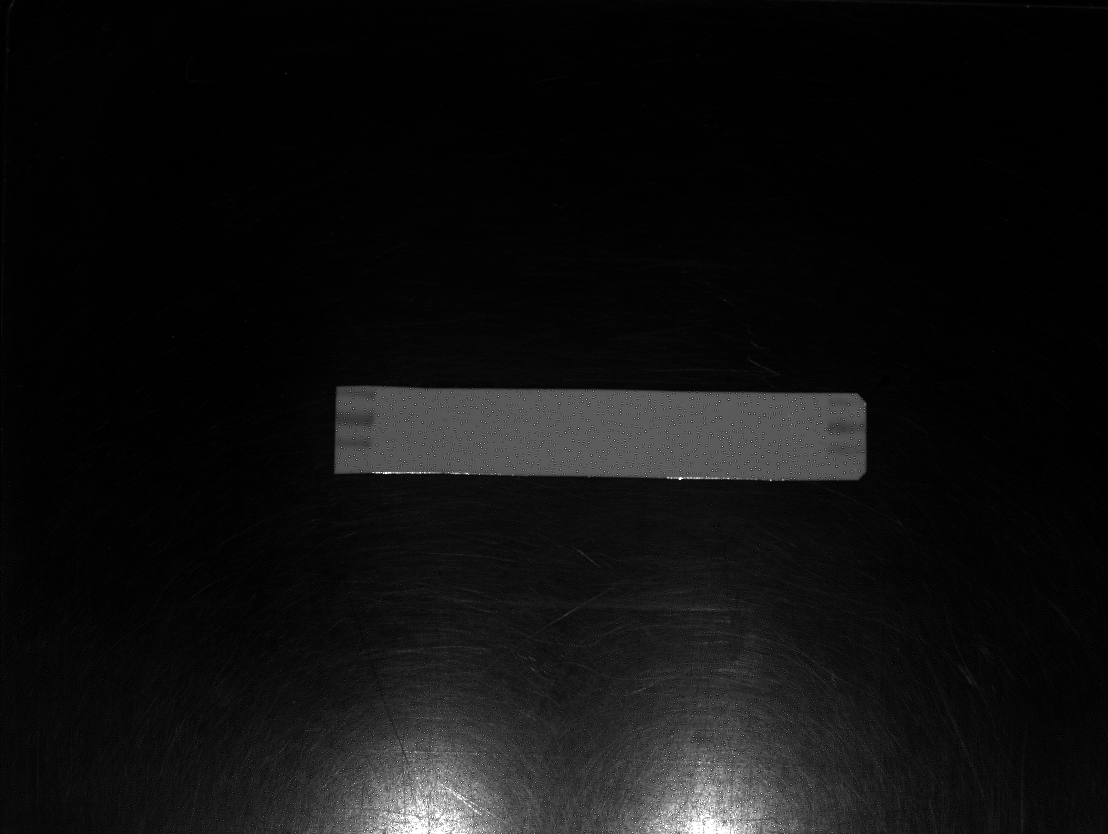

Supplement: Supplementary file 6 [file DataSheet2.zip › Data Sheet 2/Figure 7/Figure 7E/p-p65/p-p65 Membrane.png]

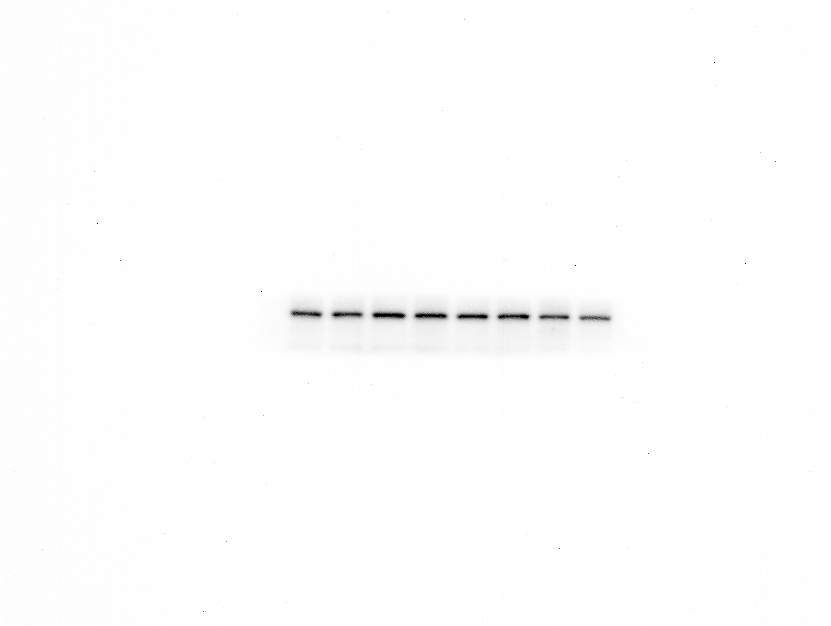

Supplement: Supplementary file 6 [file DataSheet2.zip › Data Sheet 2/Figure 7/Figure 7E/p-p65/p-p65.jpg]

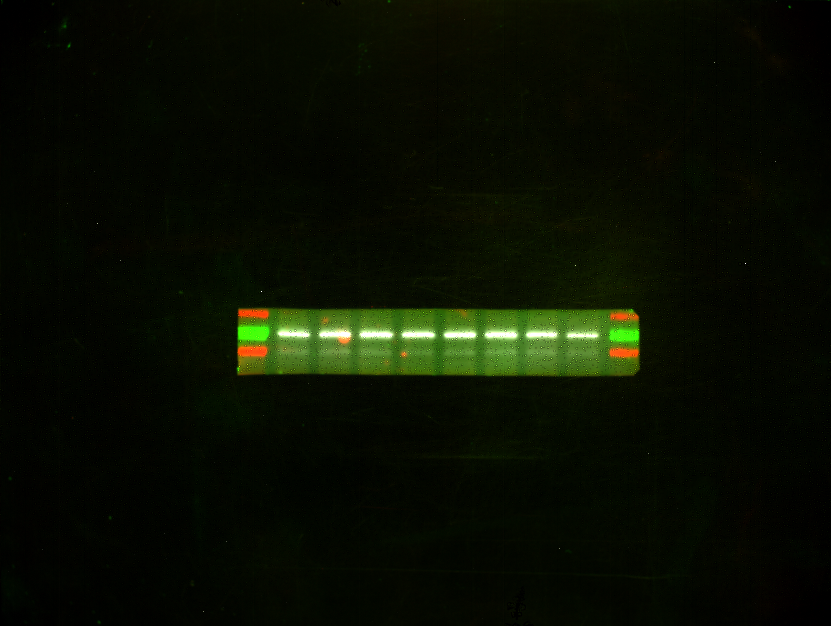

Supplement: Supplementary file 6 [file DataSheet2.zip › Data Sheet 2/Figure 7/Figure 7E/p65/p65 Marker.png]

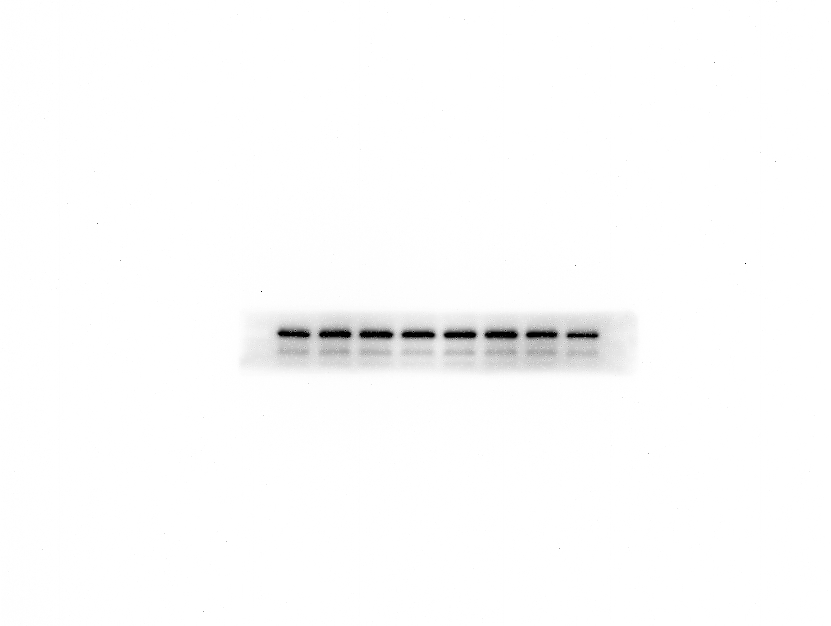

Supplement: Supplementary file 6 [file DataSheet2.zip › Data Sheet 2/Figure 7/Figure 7E/p65/p65.jpg]
