# Supplementary material for: Time Series Transcriptomic Analysis by RNA Sequencing Reveals a Key Role of PI3K in Sepsis-Induced Myocardial Injury in Mice
Source: Front Physiol. 2022 Jun 1;13:903164. doi: 10.3389/fphys.2022.903164 (PMC9198581; doi:10.3389/fphys.2022.903164)
Supplement: Supplementary file 4 [file DataSheet1.zip › Data Sheet 1/Data Sheet Legend.docx]

The raw data are uploaded as ‘Data Sheet’.

The data of gene expression files are available at the Gene Expression Omnibus website under Accession No. GSE171546 (presented in Figure 2, 3, 4, and 6).
