# Supplementary material for: Time Series Transcriptomic Analysis by RNA Sequencing Reveals a Key Role of PI3K in Sepsis-Induced Myocardial Injury in Mice
Source: Front Physiol. 2022 Jun 1;13:903164. doi: 10.3389/fphys.2022.903164 (PMC9198581; doi:10.3389/fphys.2022.903164)
Supplement: Supplementary file 5 [file Table2.DOCX]

**Supplementary Table 2**. Ten genes identified by gene co-expression network with degree, betweenness centrality, and k-core value

| Gene symbol | Gene Title | Degree | Betweenness  Centrality | k-core |
| --- | --- | --- | --- | --- |
| Pik3r1 | phosphoinositide-3-kinase regulatory subunit 1 | 26 | 0.034003726 | 8 |
| Pik3r5 | phosphoinositide-3-kinase regulatory subunit 5 | 26 | 0.029969106 | 8 |
| Pik3cg | phosphatidylinositol-4,5-bisphosphate 3-kinase catalytic subunit gamma | 25 | 0.029669981 | 8 |
| Pik3cd | phosphatidylinositol-4,5-bisphosphate 3-kinase catalytic subunit delta | 25 | 0.032424374 | 8 |
| Plcb1 | phospholipase C beta 1 | 24 | 0.075079978 | 8 |
| Plcg1 | phospholipase C gamma 1 | 21 | 0.088140522 | 8 |
| Prkaca | protein kinase cAMP-activated catalytic subunit alpha | 19 | 0.048988348 | 8 |
| Nras | NRAS proto-oncogene, GTPase | 17 | 0.039932909 | 8 |
| Prkcb | protein kinase C beta | 17 | 0.070327033 | 8 |
| Prkca | protein kinase C alpha | 16 | 0.061078437 | 8 |
